# New Diterpenoids from *Mesona procumbens* with Antiproliferative Activities Modulate Cell Cycle Arrest and Apoptosis in Human Leukemia Cancer Cells

**DOI:** 10.3390/ph14111108

**Published:** 2021-10-29

**Authors:** Hung-Tse Huang, Chia-Ching Liaw, Yu-Chi Lin, Geng-You Liao, Chih-Hua Chao, Chun-Tang Chiou, Yao-Haur Kuo, Kung-Ta Lee

**Affiliations:** 1 Division of Chinese Materia Medica Development, National Research Institute of Chinese Medicine, Ministry of Health and Welfare, Taipei 11201, Taiwan; kk49310953@nricm.edu.tw (H.-T.H.); liawcc@nricm.edu.tw (C.-C.L.); yclin@nricm.edu.tw (Y.-C.L.); ctchiou@nricm.edu.tw (C.-T.C.); 2 Department of Biochemical Science and Technology, National Taiwan University, Taipei 10617, Taiwan; 3 Department of Biochemical Science and Technology, National Chiayi University, Chiayi 60004, Taiwan; 4 Department of Marine Biotechnology and Resources, National Sun Yat-sen University, Kaohsiung 80424, Taiwan; 5 Institute of Physiology, School of Medicine, National Yang Ming Chiao Tung University, Taipei 11201, Taiwan; pottershows@gmail.com; 6 School of Pharmacy, China Medical University, Taichung 40402, Taiwan; chchao@mail.cmu.edu.tw; 7 Graduate Institute of Integrated Medicine, College of Chinese Medicine, China Medical University, Taichung 40402, Taiwan

**Keywords:** *Mesona procumbens*, diterpenoids, mesonol, apoptosis, cell cycle arrest

## Abstract

*Mesona procumbens* is a popular material used in foods and herbal medicines in Asia for clearing heat and resolving toxins. However, phytochemical research on this plant is very rare. In this study, eleven new diterpenoids, mesonols A-K (**1–11**), comprising seven *ent*-kauranes, three *ent*-atisanes, and one sarcopetalane, were isolated from its methanolic extract. Structural elucidation of compounds **1–11** was performed by spectroscopic methods, especially 2D NMR, HRESIMS, and X-ray crystallographic analysis. All isolates were assessed for their antiproliferative activity, and compounds **1–4** showed potential antiproliferative activities against A549, Hep-3B, PC-3, HT29, and U937 cancer cells, with IC_50_ values ranging from 1.97 to 19.86 µM. The most active compounds, **1** and **2**, were selected for further investigation of their effects on cell cycle progression, apoptosis, and ROS generation in U937 human leukemia cancer cells. Interestingly, it was found that compounds **1** and **2** induced antiproliferative effects in U937 cells through different mechanisms. Compound **1** caused cell cycle arrest at the G2/M phase and subsequent cell death in a dose- and time-dependent manner. However, **2**-mediated antiproliferation of U937 cells triggered ROS-mediated mitochondrial-dependent apoptosis. These results provide insight into the molecular mechanism involved in the antiproliferative activities of compounds **1** and **2** in U937 cells. Altogether, the study showed that new diterpenoid compounds **1** and **2** from *M. procumbens* are potent and promising anticancer agents.

## 1. Introduction

Natural products (NPs) and NP-like compounds have played an important role in drug discovery. More than 70% of the anticancer drugs that have been approved worldwide over the past seven decades are NPs or are inspired by NP structures [1,2]. NPs have evolved to target multiple proteins and often possess diverse biological activities, leading to a combination of therapeutic effects and toxicity. NP scaffolds can be regarded as “bioactive” or “privileged” scaffolds in chemical space because they have been naturally selected to specifically interact with diverse biological targets [3]. Due to the toxicity of the therapeutics that are currently used to treat various types of tumors, several NPs and their structural derivatives have been evaluated for their potential as anticancer agents [4,5]. A wide range of NPs, e.g., terpenoids [6], flavonoids [7], alkaloids [8], polyphenols [9], and other secondary metabolites, have shown promising anticancer properties.

**Figure 1:**
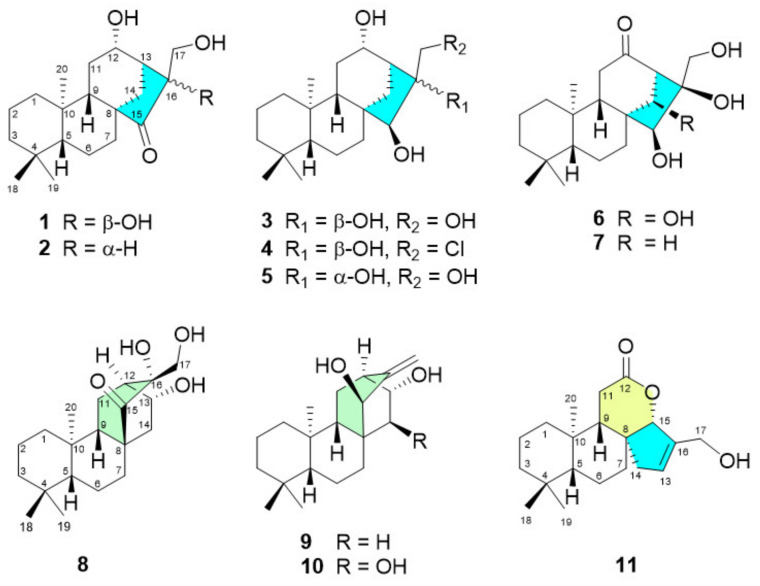
Structures of the isolated diterpenes **1**–**11** from the methanolic extract of *M. procumbens*.

Apoptosis is a mechanism of cell death that involves cell shrinkage and membrane blebbing (morphological changes) and is triggered by various death stimuli, including stress, heat, and UV radiation [10,11]. The two major apoptosis pathways are the extrinsic pathway, which involves the binding of ligands to cell surface “death receptors”, and the intrinsic pathway, which involves the mitochondria [12]. Apoptosis also involves the activation of caspases, a family of cysteine proteases and members of the Bcl-2 family [13]. The caspase family can be divided into two categories: initiator and effector caspases. The activation of initiator caspases, such as caspase-2, -8, -9, and -10, occurs through the cleavage of an aspartate residue that triggers the execution of the actions of effector caspases, such as caspase-3, -6, and -7. Traditional Chinese herbs are considered to have great potential for cancer treatment and are attracting increasing attention. Hsian-tsao (*Mesona procumbens* Hemsl.), a traditional Chinese herb, is used for the treatment of heat shock, hypertension, diabetes, liver disease, and muscle pain. In addition, Hsian-tsao tea and its herbal jelly are popular during the summer, and heated herbal jelly is admired by many Taiwanese individuals, especially in the winter, due to its aroma and taste. Previous research reported that *M. procumbens* extracts possess several bioactivities, such as anti-inflammatory [14], antihypertensive [15], DNA damage protection [16], antimutagenic [17], liver fibrosis prevention [18], and renal protective [19] activities. However, the bioactivity and components of *M. procumbens* in relation to the treatment of cancer remain unknown. This study describes the isolation and structural elucidation of eleven new diterpenoids, comprising seven *ent*-kauranes (mesonols A-G, **1**–**7**), three *ent*-atisanes (mesonols H-J, **8**–**10**), and one sarcopetalane (mesonol K, **11**) (Figure 1). Their absolute stereochemistry was established by NOESY experiments, biosynthesis, and X-ray crystallographic analysis. Previous pharmacological studies have shown that *ent*-kaurane diterpenes from other plants have potential anticancer activity that is mediated mainly through the regulation of cell cycle arrest and apoptosis [20]. Herein, the cytotoxicity of all of the isolated diterpenoids against five human cancer cell lines, A549, Hep-3B, PC-3, HT29, and U937, is reported. Compounds **1** and **2** were further investigated for their cytotoxicity against the U937 cell line. Supplementary materials can be found, in the online version, including ^1^H, ^13^C, 2D NMR, and HRESIMS spectra for compounds **1**–**11**.

## 2. Results

### 2.1. General

The methanolic extract of *M. procumbens* was suspended in H_2_O and then successively partitioned with *n*-hexane and CH_2_Cl_2_ to give two organic layers and an aqueous layer. The CH_2_Cl_2_ extract was chromatographed on a C_18_ gel flash column and then on a silica gel flash column. The subfractions were further subjected to preparative HPLC using a reversed-phase column to yield eleven new diterpenoids (**1**–**11**) (Figure 1). All isolated compounds were screened for their antiproliferative activities against five human cancer cell lines, and their molecular mechanism was investigated in U937 human leukemia cancer cells.

### 2.2. Structure Elucidation of the Isolated Diterpenoids

**Figure 2:**
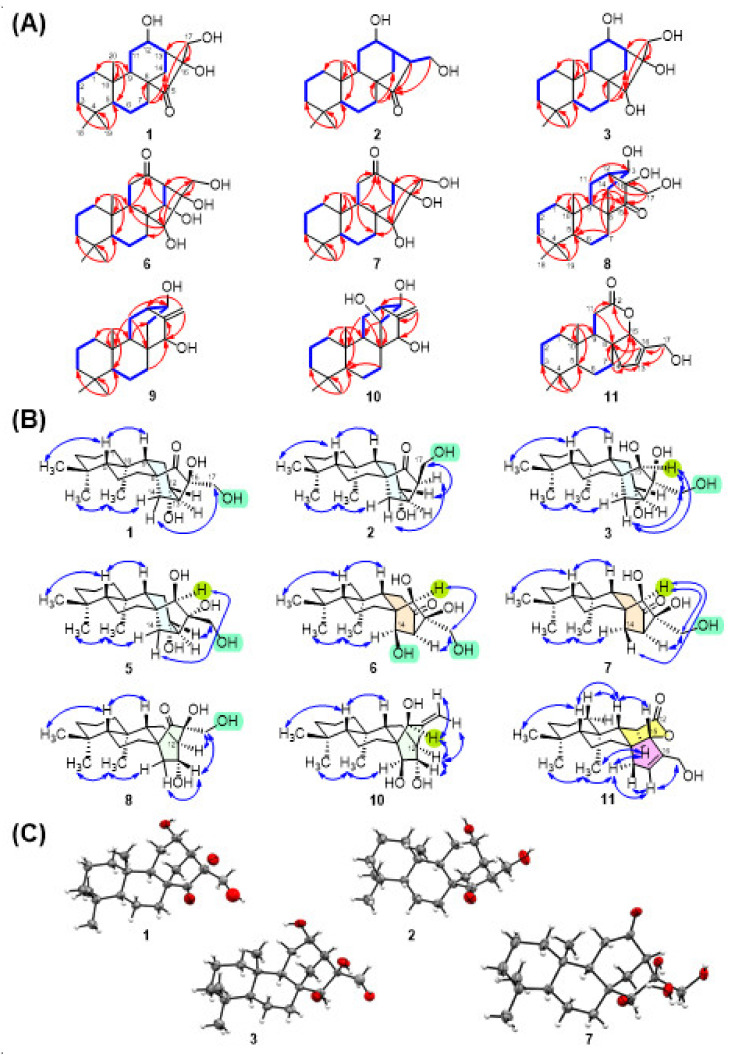
2D-NMR correlations and X-ray ORTEP drawings of the isolates. (**A**) ^1^H-^1^H COSY (─) and key HMBC (→) correlations of **1**–**3** and **6**–**11**. (**B**) Main NOESY correlations of **1**–**3**, **5**–**8** and **10**–**11**. (**C**) X-ray ORTEP drawings of **1**–**3** and **7**.

Compound **1** was isolated as colorless needles (MeOH), whose molecular formula was determined to be C_20_H_32_O_4_ at *m*/*z* 359.2193 [M + Na]^+^ (calcd. for C_20_H_32_O_4_Na_,_ 359.2193) by HRESIMS, requiring five degrees of unsaturation in the molecule. The IR spectrum exhibited absorption bands diagnostic of hydroxy (3468 cm^−1^) and ketone (1716 cm^−1^) functionalities. With the aid of the HSQC spectrum, the ^1^H and ^13^C NMR spectra (Table 1 and Table 2) of **1** displayed 20 carbon signals corresponding to three tertiary methyl groups (*δ*_H_ 0.83; *δ*_C_ 20.4, *δ*_H_ 0.87; *δ*_C_ 32.6, and *δ*_H_ 1.32; *δ*_C_ 15.2), eight methylenes (including one oxygenated group, *δ*_H_ 3.40 and 3.55 (each 1H, d, *J* = 11.4 Hz); *δ*_C_ 64.0), four methines (including one oxygenated, *δ*_H_ 4.12 (m); *δ*_C_ 65.6), and five quaternary carbons (including one oxygenated (*δ*_C_ 81.2) and one carbonyl (*δ*_C_ 223.0)). Analysis of the ^1^H-^1^H COSY and HMBC spectra (Figure 2A) corroborated the planar structure of **1**. The COSY spectrum showed cross-peaks for H-1/H-2/H-3, H-5/H-6/H-7, and H-11/H-12/H-13/H-14, establishing the presence of three fragments. Furthermore, the HMBC correlations from geminal methyl protons CH_3_-18 and CH_3_-19 to C-3, C-4, and C-5; from CH_3_-20 to C-1, C-5, C-9, and C-10; from H_2_-7 to C-8; from H_2_-14 to C-8, C-12, C-15, and C-16; and from the oxygenated methene H_2_-17 to C-13, C-15, and C-16, constructing the kaurane-type diterpene skeletal structure of **1** [15,16]. Additionally, the ketone group at C-15 and three hydroxy groups at C-12, C-16, and C-17 were deduced from their chemical shifts and mass data. Thus, the fully planar structure of **1** was established as a 12,16,17-trihydroxy-kaur-15-one. The NOESY spectrum of **1** exhibited (Figure 2B) correlations between H-5 and H-9 and between CH_3_-20 and one proton of H-14 (δ_H_ 2.73 (brd, *J* = 13.2)), revealing that H-5 and H-9 were β-oriented, while CH_3_-20 was α-oriented. Thus, the stereochemical features of ring junction protons implied that **1** was an *ent*-kaurane diterpene [21]. Furthermore, the protons of H_2_-17 (δ_H_ 3.40, 3.55 (d, *J* = 11.4)), positioned in an equatorial orientation, showed NOE correlations with the other proton H-14 (δ_H_ 1.34), revealing that the hydroxylmethyl group was α-oriented. The absolute configuration of compound **1** was further determined by CuKα X-ray crystallographic analysis (Figure 2C). Finally, the structure of compound **1** was unambiguously elucidated as 12α,16β,17-trihydroxy-*ent*-kaur-15-one and named mesonol A.

Compound **2** had a molecular formula of C_20_H_32_O_3_, as determined by a positive HRESIMS experiment. Comparison of the ^1^H- and ^13^C-NMR spectra of **2** with those of **1** showed that the oxygenated quaternary carbon C-16 (δ_C_ 81.2) in **1** was replaced by a methine carbon (δ_H_ 2.63; δ_C_ 55.2) in **2**. This result was confirmed by H_2_-17 (δ_H_ 3.60, 3.92) and H-13 (δ_H_ 2.67), which both showed a COSY correlation with H-16, and a 2-(hydroxymethyl)cyclopentan-1-one unit was constructed by H-14, H-16, and H_2_-17, showing HMBC correlations with C-15 (δ_C_ 222.7) (Figure 2A). The stereochemistry of kaurane-type diterpene **2** was identical to that in **1** because of the same NOE correlations (H-5/H-9, CH_3_-19/CH_3_-20/H_α_-14 (δ_H_ 2.86)) (Figure 2B). Additionally, the NOESY correlation between H-16 and H_β_-14 (δ_H_ 1.24) revealed that H-16 was α-oriented, while a correlation between H_β_-14 and H_2_-17 was absent. Moreover, the β-orientation of H-12 was deduced from the NOE correlations between H-12 and H_2_-17. The configuration was further supported by the single-crystal X-ray crystallographic data of **2** (Figure 2C). Therefore, the structure of **2** was clearly established as 12α-hydroxy-16β-hydroxymethyl-*ent*-kaur-15-one.

A 15,16-dihydroxy-*ent*-kaurane diterpene skeleton was elucidated for each of the diterpenes **3**–**7** from the NMR spectra, and all showed the presence of three methines (C-5, C-9, and C-13), three quaternary carbons (C-4, C-8, and C-10), an oxygenated methine (C-15), and an oxygenated quaternary carbon (C-16). According to ^1^H-^1^H COSY and HMBC correlations (Figure 2A), compound **3** was elucidated as 12,15,16,17-tetrihydroxy-*ent*-kaurane. Thus, the planar structure of **3** is a reductive product of **1**. The carbonyl carbon (C-15) in **1** is replaced with a hydroxyl group in **3** (δ_C_ 80.3). OH-15 was assigned the β-orientation due to the upfield shift of C-9 (δ_C_ 47.5), which was caused by the *γ*-gauche steric compression effect between OH-15 and H-9. Moreover, H-15 (δ_H_ 3.10) showed NOE correlations (Figure 2B) with H_2_-17, and one proton of H_2_-14 (δ_H_ 0.72) revealed that those protons were on the same side of the cyclopentane ring. From the above findings, the stereochemistry of OH-16 showed an axial-like β-orientation, while the stereochemistry of the 16-hydroxymethyl group showed an equatorial-like α-orientation. Furthermore, the configuration was confirmed by single-crystal X-ray diffraction analysis, and a perspective drawing of **3** is provided in Figure 2C. Finally, the structure of **3** was assigned as 12α,15β,16β,17-tetrahydroxy-*ent*-kaurane and named mesonol C.

Comparison of the ^13^C NMR and ^1^H NMR data (Table 1 and Table 2) of compound **4** with those of **3** revealed that the only difference was the substitution of a hydroxyl group at C-17 in compound **3** with a chlorine in **4**. This finding was confirmed by the chemical shift of C-17 occurring upfield to δ_C_ 53.9 in **4**. In addition, the HRESIMS spectrum of **4** showed a pair of sodiated quasimolecular ion peaks at *m/z* 379.2016 [M + Na]^+^ and 381.1985 [M + Na + 2]^+^ with a ratio of 3:1, suggesting that compound **4** possesses a chlorine atom. The relative configurations of the chiral centers in **4** were found to be the same as those in **3**, as determined from a NOESY experiment. Thus, **4** was elucidated as 12α,15β,16β-trihydroxy-17-chloro-*ent*-kaurane and named mesonol D. 

Compound **5** exhibited the same molecular formula (C_20_H_34_O_4_) and similar NMR data to **3**. These two compounds were determined to be stereoisomers because, after detailed analysis of their 2D NMR spectra, their planar structures were identical. The NOESY experiment (Figure 2B) determined the relative configurations of compound **5**, in which correlations were observed from CH_3_-18 to H-5, from H-5 to H-9, from CH_3_-20 to H_α_-14, from H_2_-7 to H-15, and from H-15 to H_β_-14. These results indicated that compound **5** also possessed an *ent*-kaurane skeleton. Moreover, other NOE correlations from H_2_-17 to H-12 and from H_2_-17 to H_β_-11 were observed in **5**, whereas H_2_-17 showed NOE correlations with H-15 and H_β_-14 in **5**. Thus, compound **5** was characterized as 12α,15β,16α,17-tetrahydroxy-*ent*-kaurane and named mesonol E. 

The molecular formula of **6** was inferred by HRESIMS to be C_20_H_32_O_5_ (*m/z* 375.2144 [M + Na]^+^). The ^1^H-^1^H COSY spectrum (Figure 2A) of **6** displayed two fragments of H-1 (δ_H_ 1.94)/H-2 (δ_H_ 1.64)/H-3 (δ_H_ 1.41) and H-5 (δ_H_ 0.95)/H-6 (δ_H_ 1.64)/H-7 (δ_H_ 2.28). The COSY fragments together with the HMBC correlations (Figure 2A) of H_3_-20 (δ_H_ 0.84)/C-1 (δ_C_ 39.7), C-5 (δ_C_ 55.4), C-9 (δ_C_ 45.4), C-10 (δ_C_ 38.8), H_3_-18 (δ_H_ 0.85)/C-3 (δ_C_ 41.5), C-4 (δ_C_ 32.8), C-5, C-19 (δ_C_ 32.5), and H-9 (δ_H_ 1.94)/C-8 (δ_C_ 51.9) established rings A and B in **6**. The protons of H-9, H-11 (δ_H_ 2.23 and 2.71), H-14 (δ_H_ 4.24), and H_2_-17 (δ_H_ 3.55 and 3.60) showed HMBC correlations with C-12 (δ_C_ 212.5), and the two COSY fragments of H-9/H-11 and H-13/H-14 supported the connectivity of ring C in **6**. Additionally, four hydroxyl groups were attached at C-14, C-15, C-16, and C-17, as demonstrated by the HMBC correlations from H-14 to C-15, H-15 to C-16 and C-17, and H_2_-17 to C-13 and C-16. Furthermore, the chemical shift of C-7 in **6** occurred upfield to 30.5 ppm compared with that in the other *ent*-kaurane isolates because of the *γ*-gauche steric compression effects of the 14-OH group. Thus, the fully planar structure of **6** was constructed from the above COSY and HMBC correlations. In the NOESY spectrum (Figure 2B), the cross peaks of CH_3_-18/H-5/H-9 (β-orientation) and H_3_-19/H_3_-20 (α-orientation) allowed the *ent*-kaurane skeleton in **6**. The key NOESY correlation between H-14 and H_3_-20 revealed that 14-OH was β-oriented. Additionally, H-15 showed NOESY correlations with quasi-equatorial protons H_2_-17 rather than H-9, implying that 15-OH and 16-OH were both β-oriented. Accordingly, **6** was determined to be 14β,15β,16β,17-tetrahydroxy-*ent*-kaur-12-one and named mesonol F. 

Compound **7** exhibited the same molecular formula (C_20_H_32_O_4_) and similar NMR data (Table 1 and Table 2) to **1**. These two compounds were determined to be isomers, differing from the other two isomers only in the oxygenation functional groups substituted at C-12 and C-15. The ^1^H-^1^H COSY and HMBC correlations (Figure 2A) revealed that the substituents at C-12 and C-15 were carbonyl and hydroxyl groups in **7**, respectively, instead of hydroxyl and carbonyl groups in **1**. Moreover, the NOE correlations (Figure 2B) of H-5/H-9, CH_3_-19/CH_3_-20/H_α_-14 (δ_H_ 2.28), and H-15/H_β_-14 (δ_H_ 1.33)/H_2_-17/H-13 revealed that 15-OH and 16-OH were both β-oriented. The stereochemistry of **7** was further supported by the results of single-crystal X-ray crystallographic analysis (Figure 2C). Compound **7** was therefore characterized as 15β,16β,17-trihydroxy-*ent*-kaur-12-one and named mesonol G. 

**Figure 3:**
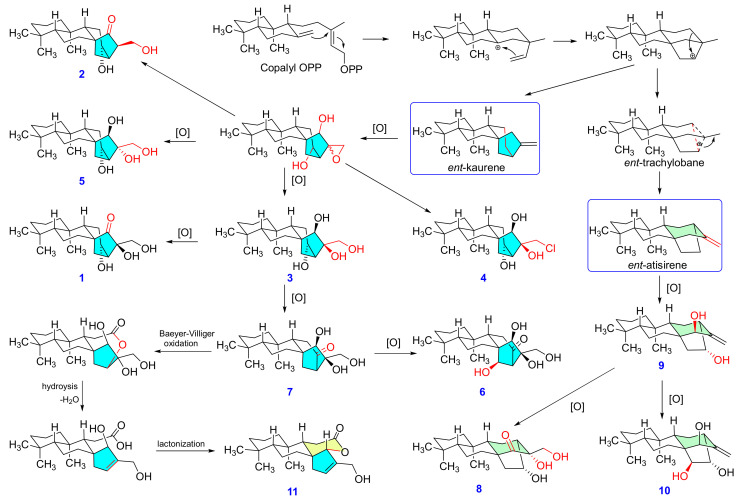
The biogenic pathway of the isolates from *M. procumbens*.

Compound **8** was isolated as a white powder and was assigned the formula C_20_H_32_O_4_ by HRESIMS. The NMR spectroscopic data (Table 1 and Table 2) of **8**, together with the molecular formula, indicated the presence of a tetracyclic diterpene with one CH_2_OH (δ_H_ 3.55, d, *J* = 12.0 Hz, 3.62, d, *J* = 12.0 Hz, δ_C_ 64.0), two hydroxyls, and one ketone (δ_C_ 216.0) functional group. From the ^1^H-^1^H COSY and HMBC correlations (Figure 2A), the structure of **8** was elucidated to have similar A, B, and C rings and most likely a structure similar to that of **1**–**7**. In detailed analysis of the HMBC spectrum, a correction from H_2_-17 and H-9 to C-12 was observed, as different from H_2_-17 to C-13 as **1**–**7**, suggesting a migrated C-12/C-16 bond rather than a C-13/C-16 bond in **8**. Therefore, compound **8** was proposed to possess a rearranged 16(13→12)-*abeo*-*ent-*kaurane skeleton (*ent*-atisane skeleton) [22]. Additionally, the position of three hydroxyl groups was assigned at C-13, C-16, and C-17, as deduced from their chemical shifts and HMBC correction. The relative configuration of rings A and B in **8** was deduced from similar NMR chemical shifts with *ent*-atisane-16α-ol [23] and *ent*-(3β,13S)-3,13-dihydroxyatis-16-en-14-one [24], along with NOESY correlations (Figure 2B) found in **8**. Consideration of the biosynthetic pathway (Figure 3) of **8** from related diterpenes isolated from the plant suggests an α-orientation of CH_3_-20 and a β-orientation of H-5. The NOESY correlation between CH_3_-20 and a proton of H-14 (δ_H_ 2.14) revealed that the C-13 and C-14 moieties had a downward orientation of bicyclo[2.2.2]octane, while the C-15/C-16/C-17 moiety had an upward orientation. Moreover, NOESY cross peaks with H-13 (δ_H_ 4.31) with H-14 (δ_H_ 1.80) and H_2_-17 (δ_H_ 3.55, 3.62) revealed that these protons were on the same side of the 2,4-dihydroxy-2-(hydroxymethyl)cyclohexan-1-one ring. Thus, the structure of **8** was completely elucidated as 13α,16α,17-trihydroxy-*ent*-atis-15-one and named mesonol H. 

Compounds **9** (mesonol I) and **10** (mesonol J) were isolated as white amorphous powders. A molecular ion peak at *m/z* 303.2324 [M − H]^−^ was found in the negative HRESIMS of **9**, corresponding to a molecular formula of C_20_H_32_O_2_. In the positive HRESIMS of **10**, *m/z* 319.2275 [M − H]^−^ indicated a molecular formula of C_20_H_32_O_3._ Comparison of the NMR data of **8** with those of **9** and **10** (Table 1 and Table 2) suggests that both structures of **9** and **10** have an *ent*-atisene skeleton. The major changes of these compounds indicated that the signals for a ketone and an oxygenated methylene in **8** were replaced by two vinyl protons with quaternary carbons in **9** and **10**. In addition to the ^1^H-^1^H COSY and HMBC correlations (Figure 2A) from H_2_-17 to C-12 and C-15, from H-15 to C-16 and C-8, from H-9 to C-14, and from H-11 to C-8 and C-13, both **9** and **10** likely processed the *ent*-atisene skeleton. In addition, an *exo*-methylene group at C-16 and C-17; two hydroxyl groups at C-13 and C-15; and three hydroxyl groups at C-13, C-14, and C-15 were found in **9** and **10**, respectively. Accordingly, **9** and **10** were determined to be 13α,15β-trihydroxy-*ent*-atis-16(17)-ene and 13α,14β,15β-trihydroxyl-*ent*-atis-16-ene, respectively. 

Compound **11** was isolated as a white amorphous powder with a specific rotation of -14.3 (*c* 0.4, MeOH). The HRESIMS data showed a sodiated quasimolecular ion peak at [M + Na]^+^ *m/z* 341.2098 (calcd. for C_20_H_30_O_3_Na, 341.2087), which indicated a molecular formula of C_20_H_30_O_3_ with six degrees of unsaturation. The ^1^H- and ^13^C-NMR spectra of **11** (Table 1 and Table 2) exhibited the presence of three tertiary methyls (δ_H_ 0.87 (s, 6H), CH_3_-19 and CH_3_-20; δ_H_ 0.89, CH_3_-18), a trisubstituted double band (δ_H_ 0.87 (s), H-13; δ_C_ 131.8, C-13; δ_C_ 142.2, C-16]) an oxygenated methylene (δ_H_ 4.14 (d, *J* = 14.4 Hz), 4.18 (d, *J* = 14.4 Hz), H_2_-17; δ_C_ 58.2), and a carbonyl (δ_C_ 173.9, C-12). In addition to the above findings, the remaining three degrees of unsaturation suggested that compound **11** contained four rings, including a lactone ring. The fully planar structure of **11** was further determined on the basis of ^1^H-^1^H COSY and HMBC correlations (Figure 2A). The frame skeleton of **11** was very similar to sarcopetalolide isolated from *Croton sarcopetalus* [25]. However, the HMBC correlations of H-15 (δ_H_ 4.77 (d, *J* = 1.8 Hz); δ_C_ 95.3) with the carbonyl (C-12) indicated that the γ-lactone was cyclized at C-15. The NOESY spectrum of **11** (Figure 2B) showed correlations between H-5/CH_3_-18 and H-9 and H-9/H-15, suggesting that H-5, H-9, H-15, and CH_3_-18 are β-oriented. According to the plausible biogenetic pathway (Figure 3), compound **11** might be derived from *ent*-kaurane diterpene mesonol G (**7**) by Baeyer–Villiger oxidation, hydrolysis, dehydration, and esterification. Finally, compound **11** was ambiguously identified as sarcopetal-13(16)-en-17-ol-12,15-olide and named mesonol K.

### 2.3. Biological Studies

Studies have shown that many isolated diterpenoid compounds exert antitumor activity against a range of cancer cell types [20]. To evaluate the effects of the isolated diterpene compounds on the growth of human cancer cells, the antiproliferative activities of compounds **1**–**11** were tested against six different cell lines, including five cancer cell lines, A549, Hep-3B, PC-3, HT29, and U937, and one normal mouse macrophage cell line, RAW 264.7. The IC_50_ values are summarized in Table 3. Among the tested compounds, mesonols A–D (**1**–**4**) showed potent antiproliferative activities against these cancer cell lines, especially against U937 cells. Of note, mesonols A (**1**) and B (**2**) exhibited remarkable antiproliferative activities against U937 cells with IC_50_ values of 2.66 and 1.97 µM, respectively, which were more active than the standard drug CPT-11 with an IC_50_ value of 4.95 µM. Moreover, mesonols A–D (**1**–**4**) were less toxic against normal mouse RAW 264.7 macrophages, implicating the selectivity of mesonols toward cancer cells. Together, these results indicated that the new diterpenoid compounds mesonols A (**1**) and B (**2**) from *M. procumbens* have potency as anticancer agents.

**Figure 4:**
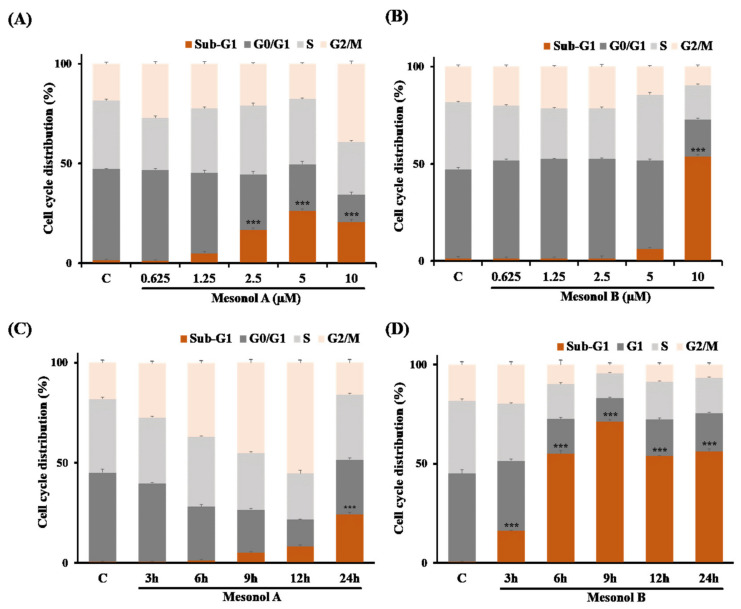
Effects of mesonols A (**1**) and B (**2**) on cell cycle progression and apoptosis in U937 cells. U937 cells were treated with various concentrations of (**A**) mesonol A or (**B**) mesonol B for 24 h and then harvested for cell cycle and sub-G1 analysis by flow cytometry, as described in Materials and Methods. For time course analysis, U937 cells were treated with (**C**) 5 µM mesonol A or (**D**) 10 µM mesonol B for 3–24 h, and cells were then subjected to cell cycle analysis at the indicated times. Values with different letters indicate significant differences, as determined by Dunnett’s method. *** *p* < 0.001 relative to the control group.

To further investigate the antiproliferative effects of mesonol A or B on U937 cancer cells, the cell cycle profiles in cells treated with various doses of mesonol A (**1**) or B (**2**) (0.625, 1.25, 2.5, 5, and 10 μM) for 24 h using flow cytometric analysis were first analyzed. Surprisingly, mesonols A (**1**) (Figure 4A) and B (**2**) (Figure 4B) showed differential effects on the cell cycle distribution in U937 cells. Mesonol A (**1**) dose-dependently increased the percentage of the sub-G_1_ population (corresponding to apoptotic cells) and reached a plateau at 5 μM. In addition, 10 μM mesonol A (**1**) not only induced the accumulation of cells at sub-G1 phase but also resulted in a significant increase in the cell population at G2/M phase, implicating that G2/M arrest may be associated with mesonol A (**1**)-mediated antiproliferation. On the other hand, mesonol B (**2**) significantly increased the sub-G1 population to 53.86% at 10 μM, compared to 6.33% at 5 μM and 1.58% for the control group. Furthermore, a time course cell cycle analysis to explore the underlying mechanism was also performed. U937 cells were treated with 5 μM mesonol A (**1**) or 10 μM mesonol B (**2**) for 3, 6, 9, 12, or 24 h. Mesonol A (**1**) led to a time-dependent accumulation of cells arrested in the G2/M phase with a concomitant gradual increase cell population of sub-G1 phase after 3 h to 12 h treatment, followed by decreased populations of G2/M and increased population of sub-G1 phase, indicating the correlation between mesonol A (**1**)-induced G2/M arrest and induction of apoptosis (Figure 4C). On the other hand, mesonol B (**2**) did not cause an obvious accumulation of cells in any phase but significantly increased apoptotic cells in the sub-G1 population (Figure 4D). These results suggested that mesonols A (**1**) and B (**2**) may exert their antiproliferative activities against U937 cells through cell cycle arrest at G2/M and induction of apoptosis, respectively.

**Figure 5:**
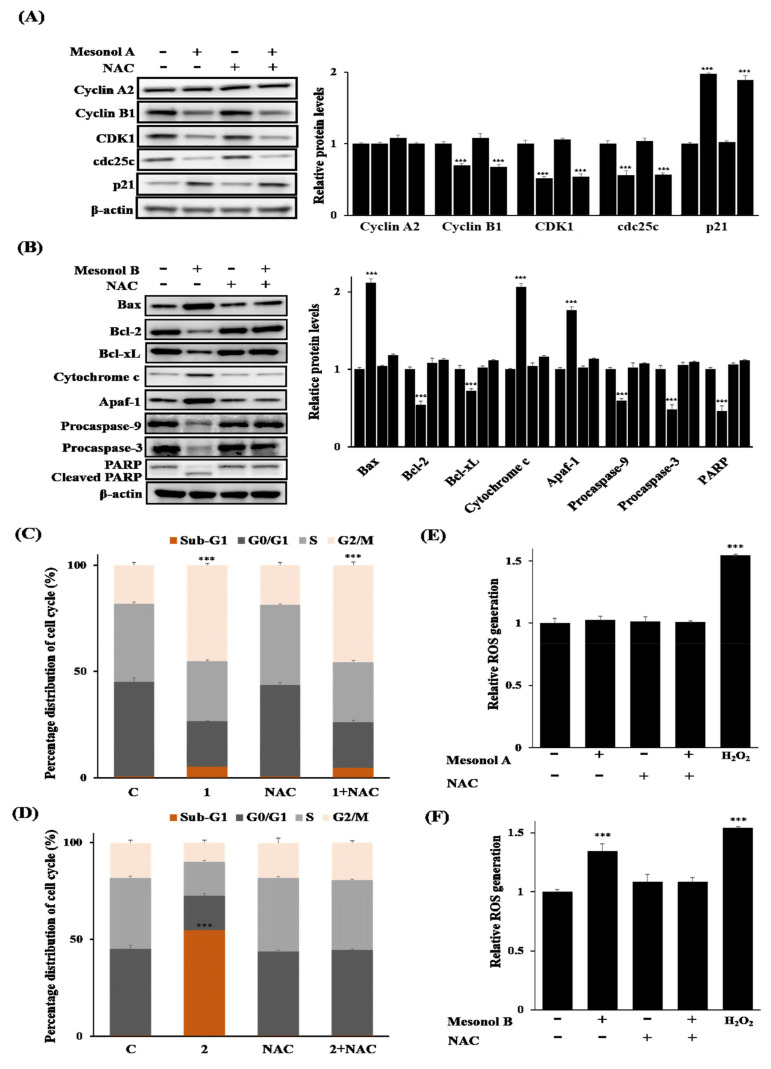
Differential effects of mesonols A (**1**) and B (**2**) on cell cycle arrest and apoptosis and the involvement of ROS. (**A**) Western blot analysis of cyclin A2, cyclin B1, CDK1, cdc25c, and p21 in U937 cells treated with 5 µM mesonol A for 12 h with or without pretreatment with 10 mM NAC for 1 h. Relative protein levels were quantified by normalization to the β-actin levels. (**B**) Western blot analysis of Bax, Bcl-2, Bcl-xL, cytochrome c, Apaf-1, procaspase-9, procaspase-3, and PARP in U937 cells treated with 10 µM mesonol B for 6 h with or without pretreatment with 10 mM NAC for 1 h. Relative protein levels were quantified by normalization to the β-actin levels. U937 cells were pretreated with or without 10 mM NAC for 1 h, followed by (**C**) 5 µM mesonol A for 12 h or (**D**) 10 µM mesonol B for 6 h, and the cell cycle distribution was analyzed by flow cytometry after PI staining. (**E**,**F**) U937 cells were treated with 5 µM mesonol A or 10 µM mesonol B for 30 min with or without pretreatment with 10 mM NAC for 1 h. H_2_O_2_ (100 µM) was used as a positive control. Intracellular ROS levels were measured by DCFH-DA, and DCF fluorescence was examined with a fluorescence plate reader as described in the Materials and methods. The values presented are the means ± SD (*n* = 3). Values with different letters indicating significant differences, as determined by Dunnett’s method. *** *p* < 0.001 relative to the control group.

To further confirm the effects of mesonols A (**1**) and B (**2**) on cell cycle arrest and apoptosis in U937 cells, the effects of mesonols A (**1**) and B (**2**) on critical cell cycle regulators and apoptosis-related proteins, respectively, were next examined. U937 cells were treated with 5 µM mesonol A (**1**) for 12 h, and the G2/M-associated cell cycle regulators p21, cyclin B1, CDK1, and cdc25c [26] were analyzed by Western blot. As shown in Figure 5A, mesonol A (**1**) caused a significant increase in p21 protein, which has been implicated in the G2/M checkpoint [27], and decreases in the G2/M progression proteins cdc25c, cyclin B1, and CDK1, confirming that mesonol A induces G2/M arrest in U937 cells. On the other hand, for mesonol B (**2**), the effects of mesonol B (**2**) on the status of apoptosis-associated proteins, including Bax, Bcl-2, Bcl-xL, cytochrome c, Apaf-1, caspase-9, caspase-3, and poly(ADP-ribose) polymerase (PARP), in U937 cells were examined (Figure 5B). Mesonol B (**2**) induced the activation of caspase-9 and -3, as indicated by decreased pro-caspase-9 and -3 protein levels. PARP, a downstream target of caspase-3, was also significantly cleaved and activated in mesonol B (**2**)-treated U937 cells. Furthermore, mesonol B (**2**) induced increases in proapoptosis-related proteins, including Bax, cytochrome c and apaf-1, and decreases in antiapoptotic proteins, including Bcl-2 and Bcl-xL. These results suggest that mesonol B (**2**) induced intrinsic mitochondrion-dependent apoptosis in U937 cells. Altogether, these results indicated that mesonol A (**1**) exerts its antiproliferative activities against U937 cells through cell cycle arrest at G2/M, whereas mesonol B (**2**) exerts its antiproliferative activities through induction of intrinsic apoptosis.

Reactive oxygen species (ROS) play a critical role in mitochondrion-mediated apoptosis. Growing evidence suggests that chemotherapy or radiotherapy could induce apoptosis in cancer cells by increasing intracellular oxidative stress [24,25]. Therefore, whether ROS are involved or not in mesonol B (**2**)-induced apoptosis and in mesonol A (**1**)-induced G2/M arrest was further investigated. To this end, whether or not the ROS scavenger *N*-acetylcysteine (NAC), which is also known to block ROS-mediated apoptosis [28], could inhibit mesonol B (**2**)-induced apoptosis in U937 cells was examined. As shown in Figure 5C, pretreatment with NAC significantly suppressed mesonol B (**2**)-induced sub-G1 accumulation compared to mesonol B (**2**) alone. Western blot analysis also showed that NAC blocked mesonol B (**2**)-induced apoptosis. Mesonol B (**2**)-induced activation of cytochrome c, Apaf-1, caspase-9, caspase-3, and PARP was significantly inhibited by NAC (Figure 5B), suggesting that mesonol B (**2**) induced apoptosis in a ROS-dependent manner. In contrast, NAC showed little to no effect on the attenuation of mesonol A (**1**)-induced G2/M arrest (Figure 5D). Consistently, NAC could not suppress mesonol A (**1**)-induced activation of p21 or inhibition of cdc25c, cyclin B1, and CDK1 proteins (Figure 5A), indicating that ROS may not be involved in mesonol A (**1**)-induced G2/M phase arrest in U937 cells.

Endogenous ROS levels were also measured by DCF-DA, an oxidation-sensitive fluorescent dye, to investigate whether ROS play differential roles in mesonol A (**1**)- and B (**2**)-mediated effects. Mesonol A (**1**) did not induce an increase in intracellular ROS levels compared to those in the control group (Figure 5E). In contrast, the intracellular ROS levels in mesonol B (**2**)-treated cells were significantly higher than those in control cells and could be blocked by pretreatment with NAC (Figure 5F). Together, these results suggest that mesonol B (**2**)-induced ROS-mediated mitochondrial-dependent apoptosis.

## 3. Discussion

In the present study, eleven new diterpenoid compounds were isolated from the CH_2_Cl_2_ layer of a methanolic extract of *M. procumbens*, including seven *ent*-kauranes, mesonols A-G (**1**–**7**), three *ent*-atisanes, mesonols H–J (**8**–**10**), and a sarcopetalane, mesonol K (**11**). Diterpenoid compounds have been reported to exert potent antitumor activity against a range of cancer cell types [20]. For example, the natural compound *ent*-16β,17α-dihydroxykaurane has been shown to exhibit cytotoxicity and apoptotic effects in the human breast cancer cell line MCF-7 [29]. Here, four diterpenoid compounds, mesonols A-B (**1–4**), were also found, and they showed significant antiproliferative activity against human cancer cell lines, including A549, Hep-3B, PC-3, HT29, and U937. Moreover, the most active mesonols A (**1**) and B (**2**) displayed remarkable inhibitory activity against U937 cell lines with IC_50_ values of 2.66 and 1.97 µM, respectively, and were even better than the standard drug CPT-11 with an IC_50_ value of 4.95 µM. Moreover, both mesonols A (**1**) and B (**2**) were less toxic against normal mouse RAW 264.7 macrophages, implicating that these compounds may act selectively against cancer cells and normal cells. These results indicated that mesonols A (**1**) and B (**2**) have high potential as anticancer agents, especially against U937 cells. Furthermore, the differential inhibitory effects of mesonols A (**1**) and B (**2**) on cell cycle arrest and/or apoptosis in U937 cancer cells were also demonstrated. An earlier study reported that glaucocalyxin A, an *ent*-kauranoid diterpenoid isolated from *Rabdosia japonica* var., induced apoptosis in HL-60 cells, as characterized by cell morphology, DNA fragmentation, activations of caspase-3 and -9, and an increased expression ratio of Bax/Bcl-2. The mitochondrial membrane potential loss and cytochrome c release were observed during the induction, and pretreatment of antioxidant NAC could block glaucocalyxin A-induced ROS generation and apoptosis [30]. Another *ent*-kaurene diterpenoid xerophilusin B has been reported to induce apoptosis and G2/M phase cell cycle arrest in KYSE-150 and KYSE-450 cells. Treatment with xerophilusin B increased the cytochrome c release from mitochondrial to cytosol and activation of caspase-9 and -3, while it downregulated caspase-7 and PARP levels. Moreover, the ratio of Bcl-2/BAX decreased after xerophilusin B treatment [31]. Similarly, our results showed that mesonol B (**2**) induced the ROS-dependent intrinsic apoptosis pathway and NAC could also block the mesonol B (**2**)-induced activation of apoptosis in U937 cells; mesonol A (**1**) caused cell cycle arrest at the G2/M phase and subsequent cell death.

The *ent*-kaurane diterpene 4α-hydroxy-17,19-*dinor*-*ent*-kaurane-16-one displayed poor anti-hepatoma potencies with IC_50_ values exceeding 100 µM [32]. Moreover *ent*-kaurene diterpenoids hebeirubescensin K (16-OH group) was 2–3 times less potent than oridonin (15-ketone) in HCT-116 cancer cells [33]. In this research, the preliminary structure-activity relationship (SAR) study revealed that the 15-ketone and/or 12-OH groups in *ent*-kaurane diterpenes are essential for the antiproliferative effects in the U937 cell lines because **3** and **4** (C-15, C12-hydroxyl groups) were 2–3 times less potent than **1** and **2** (C15-ketone, C12-hydroxyl groups), but **6** and **7** (C15-hydroxyl groups, C12-ketone) possessed slightly weaker activity than **1** and **2**. 

Thus, our bioactive natural product studies on *M. procumbens* implying mesonols A (**1**) and B (**2**) may provide drug candidates for the treatment of cancer.

## 4. Materials and Methods

### 4.1. General

Optical rotations were determined using a JASCO P-2000 polarimeter. Infrared (IR) spectra were recorded on a Mattson Genesis II spectrometer (Thermo). High-resolution electrospray ionization mass spectrometry (HRESIMS) data were acquired on a Thermo Scientific Q Exactive Focus Orbitrap liquid chromatography-tandem mass spectrometry (LC-MS/MS) instrument equipped with an Ultimate 3000 UHPLC system. Nuclear magnetic resonance (NMR) spectra were recorded on Bruker Avance 400 MHz, Varian Unity Inova 500 MHz, and Varian VNMRS 600 MHz spectrometers. Silica gel 60 (70–230 and 230–400 mesh, Merck) and Sephadex LH-20 (GE) were used for column chromatography. Precoated silica gel plates (Merck 60 F-254) were used for thin-layer chromatography (TLC), and the spots were detected by spraying with an anisaldehyde-sulfuric acid solution (5% H_2_SO_4_) followed by heating at 110 °C. HPLC was performed on a system with a Shimadzu LC-8A pump and a UV SPD-20A detector equipped with a 250 × 20 mm i.d. preparative Cosmosil 5C_18_ AR-II column (Nacalai Tesque). Single-crystal X-ray diffraction measurements were acquired with a Bruker D8 VENTURE single-crystal XRD diffractometer equipped with dual radiation sources.

### 4.2. Plant Material

Whole plants of *M. procumbens* Hemsl. (8.0 kg dry weight) were purchased from Starsci Biotech Co. Ltd., Taoyuan, and identified by Dr. Syh-Yuan Hwang of the Endemic Species Research Institute, Council of Agriculture, Taiwan. A voucher specimen (no. NRICM20190901) was deposited in the National Research Institute of Chinese Medicine, Ministry of Health and Welfare, Taipei, Taiwan. 

### 4.3. Extraction and Isolation

Air-dried *M. procumbens* (8.0 kg) was extracted three times with 100% methanol (80 L each time) at 50 °C, and the combined extracts were concentrated under reduced pressure. The methanol extract (approximately 796.5 g) was suspended in H_2_O, and the suspension was successively partitioned with n-hexane and CH_2_Cl_2_ and then concentrated under reduced pressure to obtain the hexane and CH_2_Cl_2_ extracts. The CH_2_Cl_2_ extract (approximately 47.6 g) was separated into 6 fractions (fractions I~VI) on a C_18_ gel flash column (60–230 mesh, 15 × 25 cm) and eluted with a solvent system of H_2_O/MeOH (0 to 100%). Fraction IV was further subjected to silica gel flash column chromatography (60–230 mesh, 15 × 20 cm) and eluted with a solvent system of CH_2_Cl_2_/acetone (5 to 100%) to afford eight subfractions (IVA-IVH). Fraction IVF was fractionated by preparative HPLC eluted with 60% acetonitrile (ACN) in H_2_O (flow rate: 10.0 mL/min) to give seven fractions (IVF1~IVF7). Fraction IVF2 was separated by HPLC and eluted with 35% ACN (flow rate: 10.0 mL/min) to give **6** (2.3 mg, retention time (Rt): 32.8 min, 0.00002875%). Fraction IVF3 was separated by HPLC eluted with 40% ACN (flow rate: 10.0 mL/min) to give **9** (3.7 mg, Rt: 29.7 min, 0.00004625%). Fraction IVF4 was separated by HPLC eluted with 40% ACN (flow rate: 10.0 mL/min) to give **1** (28.1 mg, Rt: 36.3 min, 0.00035125%). Fraction IVE was separated by HPLC eluted with 55% ACN (flow rate: 10.0 mL/min) to afford nine fractions (IVE1~IVE9). Fraction IVE3 was purified by preparative HPLC eluted with 45% ACN (flow rate: 10.0 mL/min) to give **8** (0.6 mg, Rt: 21.4 min, 0.0000075%). Fraction IVE4 was purified by preparative HPLC eluted with 45% ACN (flow rate: 10.0 mL/min) to give **3** (23.1 mg, Rt: 29.5 min, 0.00028875%) and **5** (15.6 mg, Rt: 32.7 min, 0.000195%). Compound **7** (5.0 mg, Rt: 39.9 min, 0.0000625%) was purified from fraction IVE5 by HPLC eluted with 45% ACN (flow rate: 10.0 mL/min). Fraction IVD was separated by HPLC eluted with 65% ACN (flow rate: 10.0 mL/min) to afford seven fractions (IVD1~IVD7). Fraction IVD5 was further purified by HPLC with repeated elution with 50% ACN (flow rate: 10.0 mL/min) to give compound **11** (2.9 mg, Rt: 42.9 min, 0.00003625%). Compounds **4** (4.1 mg, Rt: 51.1 min, 0.00005125%) and **10** (1.1 mg, Rt: 50.3 min, 0.00001375%) were purified from fraction IVD6 by HPLC eluted with 50% ACN (flow rate: 10.0 mL/min). By elution with 70% ACN (flow rate: 10.0 mL/min), fraction IVC was separated by HPLC to afford eight fractions (IVC1~ IVC8). Fraction IVC7 was further purified by HPLC eluted with 55% ACN (flow rate: 10.0 mL/min) to give **2** (9.0 mg, Rt: 23.4 min, 0.0001125%). 

### 4.4. Spectral Measurements

Mesonol A (**1**): White amorphous powder; [α${]}_{D}^{25}$ +8.1 (*c* 0.4, MeOH); IR (KBr) *ν*_max_ 3468, 2935, 2870, 1716, 1460, 1435, 1373, 1321, 1165, 1068 cm^−1^; ^1^H- and ^13^C-NMR spectroscopic data (methenol-*d*_4_) are shown in Table 1 and Table 2, respectively; HRESIMS *m/z* 359.2193 [M + Na]^+^ (calcd. for C_20_H_32_O_4_Na, 359.2193). 

Mesonol B (**2**): White amorphous powder; [α${]}_{D}^{25}$ -71.5 (*c* 0.4, MeOH); IR (KBr) *ν*_max_ 3478, 2935, 2870, 1730, 1716, 1460 cm^−1^; ^1^H- and ^13^C-NMR spectroscopic data (methenol-*d*_4_) are shown in Table 1 and Table 2, respectively; HRESIMS *m/z* 343.2252 [M + Na]^+^ (calcd. for C_20_H_32_O_4_Na, 343.2244). 

Mesonol C (**3**): White amorphous powder; [α${]}_{D}^{25}$ -44.3 (*c* 0.4, MeOH); IR (KBr) *ν*_max_ 3437, 2922, 2868, 1461, 1440, 1381, 1294, 1217, 1098 cm^−1^; ^1^H- and ^13^C-NMR spectroscopic data (methanol-*d*_4_) are shown in Table 1 and Table 2, respectively; HRESIMS *m/z* 361.2356 [M + Na]^+^ (calcd. for C_20_H_34_O_4_Na, 361.2349). 

Mesonol D (**4**): White amorphous powder; [α${]}_{D}^{25}$ -42.7 (*c* 0.4, MeOH); IR (KBr) *ν*_max_ 3362, 2925, 2870, 1460, 1440, 1371, 1294, 1098 cm^−1^; ^1^H- and ^13^C-NMR spectroscopic data (methenol-*d*_4_) are shown in Table 1 and Table 2, respectively; HRESIMS *m/z* 379.2016 [M + Na]^+^ (calcd. for C_20_H_33_O_3_Cl^35^, 379.2016), *m/z* 381.1985 [M + Na + 2]^+^ (calcd. for C_20_H_33_O_3_Cl^37^, 381.1986). 

Mesonol E (**5**): White amorphous powder; [α${]}_{D}^{25}$ -14.1 (*c* 0.4, MeOH); IR (KBr) *ν*_max_ 3355, 2930, 2868, 1457, 1438, 1368, 1291, 1101 cm^−1^; ^1^H- and ^13^C-NMR spectroscopic data (methenol-*d*_4_) are shown in Table 1 and Table 2, respectively; HRESIMS *m/z* 337.2392 [M − H]^-^ (calcd. for C_20_H_33_O_4_, 337.2373). 

Mesonol F (**6**): White amorphous powder; [α${]}_{D}^{25}$ +4.2 (*c* 0.4, MeOH); IR (KBr) *ν*_max_ 3332, 2925, 2868, 1689 cm^−1^; ^1^H- and ^13^C-NMR spectroscopic data (methenol-*d*_4_) are shown in Table 1 and Table 2, respectively; HRESIMS *m/z* 375.2144 [M + Na]^+^ (calcd. for C_20_H_32_O_5_Na, 375.2142). 

Mesonol G (**7**): White amorphous powder; [α${]}_{D}^{25}$ +41.8 (*c* 0.4, MeOH); IR (KBr) *ν*_max_ 3336, 2929, 2867, 1693, 1457, 1386, 1363, 1091, 1056 cm^−1^; ^1^H- and ^13^C-NMR spectroscopic data (methenol-*d*_4_) are shown in Table 1 and Table 2, respectively; HRESIMS *m/z* 359.2196 [M + Na]^+^ (calcd. for C_20_H_32_O_4_Na, 359.2193). 

Mesonol H (**8**): White amorphous powder; [α${]}_{D}^{25}$ +34.7 (*c* 0.4, MeOH); IR (KBr) *ν*_max_ 3447, 2929, 2872, 1718, 1457, 1073 cm^−1^; ^1^H- and ^13^C-NMR spectroscopic data (methenol-*d*_4_) are shown in Table 1 and Table 2, respectively; HRESIMS *m/z* 359.2197 [M + Na]^+^ (calcd. for C_20_H_32_O_4_Na, 359.2193). 

Mesonol I (**9**): White amorphous powder; [α${]}_{D}^{25}$ -14.9 (*c* 0.4, MeOH); IR (KBr) *ν*_max_ 3372, 2922, 2868, 2361, 2339 cm^−1^; ^1^H- and ^13^C-NMR spectroscopic data (methenol-*d*_4_) are shown in Table 1 and Table 2, respectively; HRESIMS *m/z* 303.2324 [M − H]^-^ (calcd. for C_20_H_31_O_2_, 303.2319). 

Mesonol J (**10**): White amorphous powder; [α${]}_{D}^{25}$ -17.3 (*c* 0.2, MeOH); IR (KBr) *ν*_max_ 3378, 2924, 2870, 1653, 1443, 1391, 1363, 1309, 1205, 1059, 1019 cm^−1^; ^1^H- and ^13^C-NMR spectroscopic data (methenol-*d*_4_) are shown in Table 1 and Table 2, respectively; HRESIMS *m/z* 319.2275 [M − H]^-^ (calcd. for C_20_H_31_O_3_, 319.2268). 

Mesonol K (**11**): White amorphous powder; [α${]}_{D}^{25}$ -14.3 (*c* 0.4, MeOH); IR (KBr) *ν*_max_ 3386, 2924, 2850, 1696, 1450, 1373, 1244, 1200, 1093, 1019 cm^−1^; ^1^H- and ^13^C-NMR spectroscopic data (methenol-*d*_4_) are shown in Table 1 and Table 2, respectively; HREIMS *m/z* 341.2098 [M + Na]^+^ (calcd. for C_20_H_30_O_3_Na, 341.2087). 

### 4.5. X-ray Crystallographic Data for 1–3, and 7

#### 4.5.1. Mesonol A (1)

The colorless crystal of **1** was obtained by natural evaporation from MeOH solution. Crystal data were measured by a Brucker D8 VENTURE single-crystal X-ray diffractometer equipped with a dual microfocus X-ray source on Cu radiation (Table S1 in the Supporting Information). Monoclinic crystal system (crystal size: 0.38 × 0.24 × 0.02 mm^3^), space group C2; unit cell dimensions, *a* = 12.9593 (6) Å, *b* = 6.5034 (3) Å, *c* = 21.1991 (10) Å, volume = 1842.79 (7) Å^3^, *Z* = 4, D_calcd_ = 1.251 Mg/m^3^, wavelength (λ) = 1.54178 Å, absorption coefficient = 0.680 mm^−1^, F(000) = 736, T = 200 K. A total of 13838 reflections were collected, of which 3092 independent reflections [R_int_ = 0.0680] with *I* > 2*σ (I)* were used for the analysis. The final indices were *R*1 = 0.08222 and *wR*2 = 0.2003 with goodness of fit = 1.075. The absolute structure parameter was 0.1(4). The crystallographic data on **1** were deposited in the Cambridge Crystallographic Data Centre (CCDC) under deposition number 2088866. 

#### 4.5.2. Mesonol B (2)

The colorless crystal of **2** was obtained by natural evaporation from MeOH solution. Crystal data were measured by a Brucker D8 VENTURE single-crystal X-ray diffractometer equipped with a dual microfocus X-ray source on Mo radiation (Table S2 in the Supporting Information). Monoclinic crystal system (crystal size: 0.70 × 0.02 × 0.01 mm^3^), space group C2; unit cell dimensions, *a* = 42.054 (9) Å, *b* =6.9024 (13) Å, *c* = 12.7220 (3) Å, volume = 3560.7 (12) Å^3^, *Z* = 8, D_calcd_ = 1.196 Mg/m^3^, wavelength (λ) = 0.71073 Å, absorption coefficient = 0.078 mm^−1^, F(000) = 1408, T = 200 K. A total of 34474 reflections were collected, of which 6269 independent reflections [R_int_ = 0.1615] with *I* > 2*σ (I)* were used for the analysis. The final indices were *R*1 = 0.0635 and *wR*2 = 0.116 with goodness of fit = 1.050. The absolute structure parameter was –1.6(19). The crystallographic data on **2** were deposited in the Cambridge Crystallographic Data Centre (CCDC) under deposition number 2088867. 

#### 4.5.3. Mesonol C (3)

The colorless crystal of **3** was obtained by natural evaporation from MeOH solution. Crystal data were measured by a Brucker D8 VENTURE single-crystal X-ray diffractometer equipped with a dual microfocus X-ray source on Mo radiation (Table S3 in the Supporting Information). Monoclinic crystal system (crystal size: 0.11 × 0.10 × 0.01 mm^3^), space group C2; unit cell dimensions, *a* = 11.599(11) Å, *b* =7.391(7) Å, *c* = 21.590(2) Å, volume = 1812(3) Å^3^, *Z* = 4, D_calcd_ = 1.241 Mg/m^3^, wavelength (λ) = 0.71073 Å, absorption coefficient = 0.084 mm^−1^, F(000) = 744, T = 200 K. A total of 10913 reflections were collected, of which 3171 independent reflections [R_int_ = 0.0599] with *I* > 2*σ (I)* were used for the analysis. The final indices were *R*1 = 0.0975 and *wR*2 = 0.2424 with goodness of fit = 1.073. The absolute structure parameter was –1(4). The crystallographic data on **3** were deposited in the Cambridge Crystallographic Data Centre (CCDC) under deposition number 2088868. 

#### 4.5.4. Mesonol G (7)

The colorless crystal of **7** was obtained by natural evaporation from MeOH solution. Crystal data were measured by a Brucker D8 VENTURE single-crystal X-ray diffractometer equipped with a dual microfocus X-ray source on Mo radiation (Table S4 in the Supporting Information). Monoclinic crystal system (crystal size: 0.74 × 0.39 × 0.02 mm^3^), space group P21; unit cell dimensions, *a* = 7.3577(8) Å, *b* =11.1296(9) Å, *c* = 22.9190(2) Å, volume = 1867.4(3) Å^3^, *Z* = 2, D_calcd_ = 1.231 Mg/m^3^, wavelength (λ) = 0.71073 Å, absorption coefficient = 0.085 mm^−1^, F(000) = 758, T = 200 K. A total of 27861 reflections were collected, of which 6563 independent reflections [R_int_ = 0.0968] with *I* > 2*σ (I)* were used for the analysis. The final indices were *R*1 = 0.0732 and *wR*2 = 0.1893 with goodness of fit = 1.025. The absolute structure parameter was 0.1(13). The crystallographic data on **7** were deposited in the Cambridge Crystallographic Data Centre (CCDC) under deposition number 2088869. 

### 4.6. Cytotoxicity Assay

A549 (human lung carcinoma), Hep-3B (human liver carcinoma), PC-3 (human prostate carcinoma), HT29 (human colon carcinoma), U937 (human monoblastic leukemia), and RAW 264.7 cells were purchased from the Food Industry Research and Development Institute (Hsinchu, Taiwan) and cultured in 10% heat-inactivated fetal bovine serum (FBS) in DMEM or RPMI-1640. Cell viability was determined by using Cell Counting Kit-8 (CCK-8; Dojindo, Rockville, MD, USA) according to the manufacturer’s instructions. All experiments were performed in triplicate.

### 4.7. Western Blot Analysis

Cells were lysed in RIPA buffer with phenylmethylsulfonyl fluoride (PMSF; Beyotime Biotechnology, Jiangsu, China). The protein concentration was determined using a Bio-Rad protein assay system (Bio-Rad, Hercules, CA, USA). Equivalent amounts of proteins were separated by SDS–PAGE and then transferred to polyvinylidene difluoride membranes (Bio-Rad). After blocking in TBS containing 5% nonfat milk, the membranes were incubated with primary antibodies (1:1000 dilution) at 4 °C for 12 h and then incubated with a horseradish peroxidase-conjugated secondary antibody (1:5000 dilution). Signals were detected on X-ray film using an ECL detection system (Pierce, Rockford, IL, USA). The relative protein levels were calculated based on β-actin as the loading control. 

### 4.8. Cell Cycle Analysis

U937 cells were treated with mesonols A (**1**) or B (**2**), dosage range: 0.625–10 µM, for the specified time period, fixed in ice-cold 70% ethanol in PBS, suspended in Krishan’s reagent (0.05 mg/mL propidium iodide (PI), 0.1% sodium citrate, 0.02 mg/mL ribonuclease A and 0.3% NP-40), and incubated on ice for 30 min. Data acquisition for 10,000 events was performed with an FACScan system (Becton Dickinson). The distribution of cells in the different phases of the cell cycle was analyzed from the DNA histograms using CELL Quest software. All experiments were performed in triplicate. 

### 4.9. Measurement of ROS Generation

U937 cells were seeded onto 96-well microplates at a final concentration of 1 × 10^5^ cells per well and treated with mesonol A or B (5 or 10 µM) for 30 min with or without pretreatment with *N*-acetylcysteine (NAC) (10 mM) for 1 h. The cells were stained with 2′,7′-dichlorofluorescin diacetate (DCFDA) Cellular Reactive Oxygen Species Detection Assay Kit (Abcam, Cambridge, UK) solution (20 µM) and then incubated at 37 °C for 30 min in the dark. Fluorescence measurements were performed with a fluorescence plate reader (Bio-Rad, Hercules, CA, USA) at Ex/Em = 485/535 nm in end point mode in the presence of compounds, medium, or buffer. All experiments were performed in triplicate. 

### 4.10. Statistical Analysis

SPSS (SPSS, Chicago, IL, USA) was used to perform statistical data analysis. All data are presented as the mean ± standard deviation. Groups were compared using one-way analysis of variance (ANOVA) followed by Tukey’s test of multiple comparisons; *p*-values ≤ 0.05 were considered statistically significant.

## 5. Conclusions

The results of the present study suggest that new diterpenoid mesonols A (**1**) and B (**2**) from *M. procumbens* have potency as anticancer agents and are more active than the standard drug CPT-11. Mesonols A and B were less toxic against normal mouse RAW 264.7 macrophages, implicating the selectivity of mesonols toward cancer cells. The anticancer mechanism of mesonols A and B was also explored to provide a theoretical basis for the development of targeted anticancer agents.

## Figures and Tables

**Table 1:** ^1^H NMR Spectroscopic Data (*δ*_H_ in ppm, mult, *J* in Hz) of **1**–**11** in Methanol-*d*_4_.

No	1 ^a^	2 ^b^	3 ^b^	4 ^b^	5 ^b^	6 ^b^	7 ^a^	8 ^a^	9 ^b^	10 ^b^	11 ^a^
1	0.71 td (3.6, 13.2) 1.74 m	0.74 td (3.5, 13.0) 1.75 m	0.82 m 1.83 m	0.82 m 1.83 m	0.82 m 1.81 m	0.88 m 1.76 brd (12.5)	0.83 m 1.76 m	0.82 m 1.65 m	0.88 m 1.61 m	0.90 m 1.56 m	0.98 td (4.2, 13.2) 1.62 m
2	1.37 m 1.60 m	1.42 m 1.70 m	1.40 m 1.68 m	1.40 m 1.68 m	1.40 m 1.69 m	1.45 m 1.66 m	1.42 m 1.65 m	1.37 m 1.64 m	1.38 m 1.64 m	1.38 m 1.64 m	1.46 m 1.64 m
3	1.16 m 1.38 m	1.18 m 1.41 m	1.17 td (4.5, 14.0) 1.39 m	1.18 td (4.5, 14.0) 1.40 m	1.17 td (4.5, 13.0) 1.40 m	1.21 td (4.5, 13.5) 1.42 m	1.21 td (4.2, 12.0) 1.39 m	1.15 m 1.38 m	1.19 m 1.40 m	1.19 td (4.0, 13.0) 1.41 m	1.22 td (3.6, 13.2) 1.42 m
5	0.88 m	0.90 m	0.83 m	0.82 m	0.83 dd (2.0, 12.5)	0.95 dd (2.0, 11.5)	0.85 dd (2.4, 11.4)	0.72 dd (1.8, 12.0)	0.82 dd (2.0, 12.0)	0.89 m	1.06 dd (2.4, 10.5)
6	1.37 m 1.66 m	1.40 m 1.65 m	1.39 m 1.57 m	1.41 m 1.58 m	1.40 m 1.57 m	1.42 m 1.63 m	1.36 m 1.64 m	1.35 m 1.58 m	1.40 m 1.55 m	1.41 m 1.56 m	1.50 m 1.69 m
7	1.34 m 1.79 td (6.0, 14.4)	1.37 m 1.83 td (6.0, 14.5)	1.40 m 1.71 m	1.43 m 1.70 m	1.49 m 1.67 m	1.44 m 2.27 m	1.55 td (6.6, 11.4) 1.77 m	1.23 m 1.85 td (4.8, 11.2)	1.11 m 1.85 td (5.0, 13.5)	1.61 m 1.88 td (3.0, 13.5)	1.37 m 1.77 m
9	1.19 m	1.17 d (9.5)	1.54 brd (10.0)	1.54 brd (9.5)	1.51 m	1.94 d (11.0)	1.88 brd (10.2)	1.35 m	1.51 m	1.57 m	1.74 dd (3.6, 13.2)
11	1.70 m (2H)	1.48 m 1.78 m	1.58 m 1.85 m	1.58 m 1.84 m	1.63 m 1.78 m	2.23 brd (18.0) 2.71 dd (11.5, 18.0)	2.22 brd (17.4) 2.63 dd (10.2, 17.4)	1.80 m 1.96 ddd (2.4, 7.2, 13.8)	1.36 m 1.97 ddd (2.0, 7.5, 13.0)	1.35 m 1.83 ddd (2.0, 8.0, 13.0)	2.32 dd (13.8, 17.4) 2.47 dd (3.6, 17.4)
12	4.12 m	4.05 m	4.07 m	4.07 m	3.96 m	-	-	2.27 brq (3.0)	2.30 brs	2.36 m	-
13	2.48 brt (3.6)	2.67 m	2.04 brt (4.5)	2.11 brt (4.0)	2.10 brs	2.77 brs	2.60 brd (4.8)	4.31 brd (9.0)	3.89 ddt (1.5, 3.5, 10.0)	3.60 m	5.91 brs
14	1.34 m 2.73 brd (13.2)	1.24 dd (4.0, 12.5) 2.86 brd (12.5)	0.71 dd (4.5, 12.5) 2.32 brd (12.5)	0.77 dd (4.5, 13.0) 2.32 brd (13.0)	1.27 dd (4.5, 12.0) 2.32 d (12.0)	4.24 brs	1.34 m 2.28 brd (13.2)	1.52 ddd (1.8, 10.2, 15.0) 2.14 brdd (3.0, 15.0)	1.31 m 1.98 dd (3.5, 15.0)	3.99 m	2.16 brd (17.4) 2.66 brd (17.4)
15	-	-	3.10 brs	3.18 brs	3.43 brs	3.65 brs	3.31 brs	-	3.47 brt (2.0)	3.98 m	4.77 d (1.8)
16	-	2.63 m	-	-		-	-	-	-	-	-
17	3.40 d (11.4) 3.55 d (11.4)	3.60 dd (8.0, 11.0) 3.92 dd (5.0, 11.0)	3.29 d (11.0) 3.45 d (11.0)	3.48 d (11.0) 3.62 d (11.0)	3.67 d (11.0) 3.72 d (11.0)	3.55 d (11.0) 3.60 d (11.0)	3.33 d (11.4) 3.42 d (11.4)	3.55 d (12.0) 3.62 d (12.0)	5.08 t (1.5) 5.17 t (1.5)	5.06 t (2.5) 5.13 t (2.5)	4.14 d (14.4) 4.18 d (14.4)
18	0.83 s	0.87 s	0.86 s	0.86 s	0.86 s	0.85 s	0.81 s	0.85 s	0.87 s	0.87 s	0.87 s
19	0.87 s	0.91 s	0.89 s	0.87 s	0.88 s	0.92 s	0.88 s	0.86 s	0.90 s	0.91 s	0.89 s
20	1.29 s	1.31 s	1.25 s	1.25 s	1.27 s	0.84 s	0.88 s	1.21 s	1.13 s	1.05 s	0.87 s

^a^ Recorded at 600 MHz. ^b^ Recorded at 500 MHz.

**Table 2:** ^13^C NMR Spectroscopic Data (*δ*_C_ in ppm, mult) of **1**–**11** in Methanol-*d*_4_.

No	1 ^a^	2 ^b^	3 ^b^	4 ^b^	5 ^b^	6 ^b^	7 ^a^	8 ^a^	9 ^b^	10 ^b^	11 ^a^
1	39.2, CH_2_	39.0, CH_2_	40.1, CH_2_	40.1, CH_2_	40.0, CH_2_	39.7, CH_2_	39.5, CH_2_	39.4, CH_2_	39.6, CH_2_	39.6, CH_2_	37.9, CH_2_
2	18.0, CH_2_	17.8, CH_2_	18.2, CH_2_	18.1, CH_2_	18.1, CH_2_	18.0, CH_2_	17.9, CH_2_	17.7, CH_2_	17.9, CH_2_	17.9, CH_2_	17.9, CH_2_
3	41.7, CH_2_	41.7, CH_2_	41.9, CH_2_	41.9, CH_2_	41.9, CH_2_	41.5, CH_2_	41.6, CH_2_	41.7, CH_2_	41.9, CH_2_	41.8, CH_2_	41.6, CH_2_
4	32.7, qC	32.7, qC	32.8, qC	32.8, qC	32.7, qC	32.8, qC	32.7, qC	32.5, qC	32.5, qC	32.5, qC	32.7, qC
5	55.2, CH	55.4, CH	55.1, CH	55.0, CH	55.2, CH	55.4, CH	55.1, CH	55.2, CH	55.2, CH	55.4, CH	55.8, CH
6	17.8, CH_2_	18.3, CH_2_	19.7, CH_2_	19.7, CH_2_	19.9, CH_2_	18.9, CH_2_	19.4, CH_2_	17.6, CH_2_	18.2, CH_2_	17.7, CH_2_	18.9, CH_2_
7	33.1, CH_2_	33.5, CH_2_	39.8, CH_2_	39.7, CH_2_	39.8, CH_2_	77.1, CH	37.8, CH_2_	29.8, CH_2_	39.6, CH_2_	37.4, CH_2_	39.2, CH_2_
8	51.5, qC	52.6, qC	45.2, qC	45.7, qC	46.9, qC	51.9, qC	46.3, qC	45.4, qC	38.9, qC	44.9, qC	43.1, qC
9	55.4, CH	54.1, CH	47.5, CH	47.4, CH	47.6, CH	45.1, CH	47.1, CH	47.7, CH	42.7, CH	43.0, CH	47.9, CH
10	38.3, qC	38.1, qC	37.6, qC	37.5, qC	37.5, qC	38.8, qC	38.7, qC	38.8, qC	37.4, qC	37.4, qC	36.6, qC
11	26.4, CH_2_	26.2, CH_2_	26.4, CH_2_	26.2, CH_2_	26.4, CH_2_	38.4, CH_2_	37.9, CH_2_	14.5, CH_2_	20.9, CH_2_	21.4, CH	26.8, CH_2_
12	65.6, CH	64.8, CH	66.3, CH	66.1, CH	65.7, CH	212.5, qC	214.3, qC	41.0, CH_2_	44.3, CH	44.8, CH	173.9, qC
13	43.9, CH	39.5, CH	46.6, CH	48.1, CH	51.1, CH	65.6, CH	57.4, CH	63.2, CH	67.4, CH	77.2, CH	131.8, CH
14	27.0, CH_2_	30.9, CH_2_	28.8, CH_2_	28.7, CH_2_	28.7, CH_2_	30.5, CH_2_	33.4, CH_2_	34.1, CH_2_	37.8, CH_2_	73.2, CH_2_	37.0, CH_2_
15	223.0, qC	222.7, qC	80.3, CH	81.3, CH	89.2, CH	70.9, CH	79.0, CH	216.0, qC	74.8, CH	67.9, CH	95.3, CH
16	81.2, qC	55.2, CH	75.8, qC	75.2, qC	81.1, qC	77.5, qC	76.9, qC	75.2, qC	155.1, qC	153.1, qC	142.2, qC
17	64.0, CH_2_	58.1, CH_2_	69.1, CH_2_	53.9, CH_2_	63.2, CH_2_	69.4, CH_2_	68.1, CH_2_	64.0, CH_2_	110.0, CH_2_	109.0, CH_2_	58.2, CH_2_
18	20.4, CH_3_	20.4, CH_3_	20.7, CH_3_	20.6, CH_3_	20.6, CH_3_	20.5, CH_3_	20.5, CH_3_	20.5, CH_3_	20.9, CH_3_	20.8, CH_3_	20.5, CH_3_
19	32.6, CH_3_	32.6, CH_3_	32.7, CH_3_	32.7, CH_3_	32.7, CH_3_	32.5, CH_3_	32.4, CH_3_	32.4, CH_3_	32.6, CH_3_	32.5, CH_3_	32.4, CH_3_
20	15.2, CH_3_	15.2, CH_3_	15.4, CH_3_	15.4, CH_3_	15.5, CH_3_	15.5, CH_3_	15.4, CH_3_	15.4, CH_3_	15.0, CH_3_	15.0, CH_3_	13.7, CH_3_

^a^ Recorded at 150 MHz. ^b^ Recorded at 125 MHz.

**Table 3:** Cytotoxic Activities of Compounds **1–11**.

Compounds	IC_50_ Values * (µM)					
	A549	Hep-3B	PC-3	HT29	U937	RAW 264.7
**1**	17.36 ± 0.28	12.08 ± 0.55	12.47 ± 0.24	9.10 ± 0.38	2.66 ± 0.14	>50
**2**	7.39 ± 0.42	7.06 ± 0.38	4.19 ± 0.31	2.78 ± 0.03	1.97 ± 0.09	>50
**3**	17.12 ± 0.36	17.08 ± 0.73	16.49 ± 0.55	14.18 ± 0.31	6.73 ± 0.36	>50
**4**	19.39 ± 0.38	19.86 ± 0.73	15.96 ± 0.21	12.64 ± 0.55	8.25 ± 0.24	>50
**5**	>20	>20	>20	>20	>20	>50
**6**	>20	>20	>20	>20	>20	>50
**7**	>20	>20	>20	>20	>20	>50
**8**	(-) ^a^	(-) ^a^	(-) ^a^	(-) ^a^	(-) ^a^	(-) ^a^
**9**	>20	>20	>20	>20	>20	>50
**10**	>20	>20	>20	>20	>20	>50
**11**	>20	>20	>20	>20	>20	>50
**CPT-11**	15.26 ± 0.42	23.21 ± 0.38	31.03 ± 0.28	15.11 ± 0.48	4.95 ± 0.43	(-) ^a^

* The 50% inhibitory concentrations after 72 h of drug treatment are represented as the means ± SD of 3 independent experiments. The IC_50_ value was measured using Cell Counting Kit-8. ^a^ The IC_50_ value was not determined.

## Data Availability

Not applicable.

## Supplementary Materials

The following are available online at https://www.mdpi.com/article/10.3390/ph14111108/s1.
