# Supplementary material for: New Diterpenoids from Mesona procumbens with Antiproliferative Activities Modulate Cell Cycle Arrest and Apoptosis in Human Leukemia Cancer Cells
Source: Pharmaceuticals (Basel). 2021 Oct 29;14(11):1108. doi: 10.3390/ph14111108 (PMC8622523; doi:10.3390/ph14111108)
Supplement: Supplementary file 1 [file pharmaceuticals-14-01108-s001.zip › pharmaceuticals-1421092-supplementary.pdf]

Supplementary data

Content

Figure S1. ^{1}H-NMR spectrum of mesonol A (**1**) in MeOD-*d*4 (600 MHz)

Figure S2. ^{13}C-NMR spectrum of mesonol A (**1**) in MeOD-*d*4 (150 MHz)

Figure S3. COSY spectrum of mesonol A (**1**)

Figure S4. HSQC spectrum of mesonol A (**1**)

Figure S5. HMBC spectrum of mesonol A (**1**)

Figure S6. NOESY spectrum of mesonol A (**1**)

Figure S7. (+)-HRESIMS spectrum of mesonol A (**1**)

Figure S8. ^{1}H-NMR spectrum of mesonol B (**2**) in MeOD-*d*4 (500 MHz)

Figure S9. ^{13}C-NMR spectrum of mesonol B (**2**) in MeOD-*d*4 (125 MHz)

Figure S10. COSY spectrum of mesonol B (**2**)

Figure S11. HSQC spectrum of mesonol B (**2**)

Figure S12. HMBC spectrum of mesonol B (**2**)

Figure S13. NOESY spectrum of mesonol B (**2**)

Figure S14. (+)-HRESIMS spectrum of mesonol B (**2**)

Figure S15. ^{1}H-NMR spectrum of mesonol C (**3**) in MeOD-*d*4 (500 MHz)

Figure S16. ^{13}C-NMR spectrum of mesonol C (**3**) in MeOD-*d*4 (125 MHz)

Figure S17. COSY spectrum of mesonol C (**3**)

Figure S18. HSQC spectrum of mesonol C (**3**)

Figure S19. HMBC spectrum of mesonol C (**3**)

Figure S20. NOESY spectrum of mesonol C (**3**)

Figure S21. (+)-HRESIMS spectrum of mesonol C (**3**)

Figure S22. ^{1}H-NMR spectrum of mesonol D (**4**) in MeOD-*d*4 (500 MHz)

Figure S23. ^{13}C-NMR spectrum of mesonol D (**4**) in MeOD-*d*4 (125 MHz)

Figure S24. COSY spectrum of mesonol D (**4**)

Figure S25. HSQC spectrum of mesonol D (**4**)

Figure S26. HMBC spectrum of mesonol D (**4**)

Figure S27. NOESY spectrum of mesonol D (**4**)

Figure S28. (+)-HRESIMS spectrum of mesonol D (**4**)

Figure S29. ^{1}H-NMR spectrum of mesonol E (**5**) in MeOD-*d*4 (500 MHz)

Figure S30. ^{13}C-NMR spectrum of mesonol E (**5**) in MeOD-*d*4 (125 MHz)

Figure S31. COSY spectrum of mesonol E (**5**)

Figure S32. HSQC spectrum of mesonol E (**5**)

Figure S33. HMBC spectrum of mesonol E (**5**)

Figure S34. NOESY spectrum of mesonol E (**5**)

Figure S35. (−)-HRESIMS spectrum of mesonol E (**5**)

Figure S36. ^{1}H-NMR spectrum of mesonol F (**6**) in MeOD-*d*4 (500 MHz)

Figure S37. ^{13}C-NMR spectrum of mesonol F (**6**) in MeOD-*d*4 (125 MHz)

Figure S38. COSY spectrum of mesonol F (**6**)

Figure S39. HSQC spectrum of mesonol F (**6**)

Figure S40. HMBC spectrum of mesonol F (**6**)

Figure S41. NOESY spectrum of mesonol F (**6**)

Figure S42. (+)-HRESIMS spectrum of mesonol F (**6**)

Figure S43. ^{1}H-NMR spectrum of mesonol G (**7**) in MeOD-*d*4 (600 MHz)

Figure S44. ^{13}C-NMR spectrum of mesonol G (**7**) in MeOD-*d*4 (150 MHz)

Figure S45. COSY spectrum of mesonol G (**7**)

Figure S46. HSQC spectrum of mesonol G (**7**)

Figure S47. HMBC spectrum of mesonol G (**7**)

Figure S48. NOESY spectrum of mesonol G (**7**)

Figure S49. (+)-HRESIMS spectrum of mesonol G (**7**)

Figure S50. ^{1}H-NMR spectrum of mesonol H (**8**) in MeOD-*d*4 (600 MHz)

Figure S51. ^{13}C-NMR spectrum of mesonol H (**8**) in MeOD-*d*4 (150 MHz)

Figure S52. COSY spectrum of mesonol H (**8**)

Figure S53. HSQC spectrum of mesonol H (**8**)

Figure S54. HMBC spectrum of mesonol H (**8**)

Figure S55. NOESY spectrum of mesonol H (**8**)

Figure S56. (+)-HRESIMS spectrum of mesonol H (**8**)

Figure S57. ^{1}H-NMR spectrum of mesonol I (**9**) in MeOD-*d*4 (500 MHz)

Figure S58. ^{13}C-NMR spectrum of mesonol I (**9**) in MeOD-*d*4 (125 MHz)

Figure S59. COSY spectrum of mesonol I (**9**)

Figure S60. HSQC spectrum of mesonol I (**9**)

Figure S61. HMBC spectrum of mesonol I (**9**)

Figure S62. (–)-HRESIMS spectrum of mesonol I (**9**)

Figure S63. ^{1}H-NMR spectrum of mesonol J (**10**) in MeOD-*d*4 (500 MHz)

Figure S64. ^{13}C-NMR spectrum of mesonol J (**10**) in MeOD-*d*4 (125 MHz)

Figure S65. COSY spectrum of mesonol J (**10**)

Figure S66. HSQC spectrum of mesonol J (**10**)

Figure S67. HMBC spectrum of mesonol J (**10**)

Figure S68. NOESY spectrum of mesonol J (**10**)

Figure S69. (−)-HRESIMS spectrum of mesonol J (**10**)

Figure S70. ^{1}H-NMR spectrum of mesonol K (**11**) in MeOD-*d*4 (600 MHz)

Figure S71. ^{13}C-NMR spectrum of mesonol K (**11**) in MeOD-*d*4 (150 MHz)

Figure S72. COSY spectrum of mesonol K (**11**)

Figure S73. HSQC spectrum of mesonol K (**11**)

Figure S74. HMBC spectrum of mesonol K (**11**)

Figure S75. NOESY spectrum of mesonol K (**11**)

Figure S76. (+)-HRESIMS spectrum of mesonol K (**11**)

Table S1. Crystal data and structure refinement for **1** (d23335a, CCDC number: 2088866).

Table S2. Crystal data and structure refinement for **2** (d22559, CCDC number: 2088867).

Table S3. Crystal data and structure refinement for **3** (d22583, CCDC number: 2088868).

Table S4. Crystal data and structure refinement for **3** (d22561, CCDC number: 2088869).

Supplementary figures

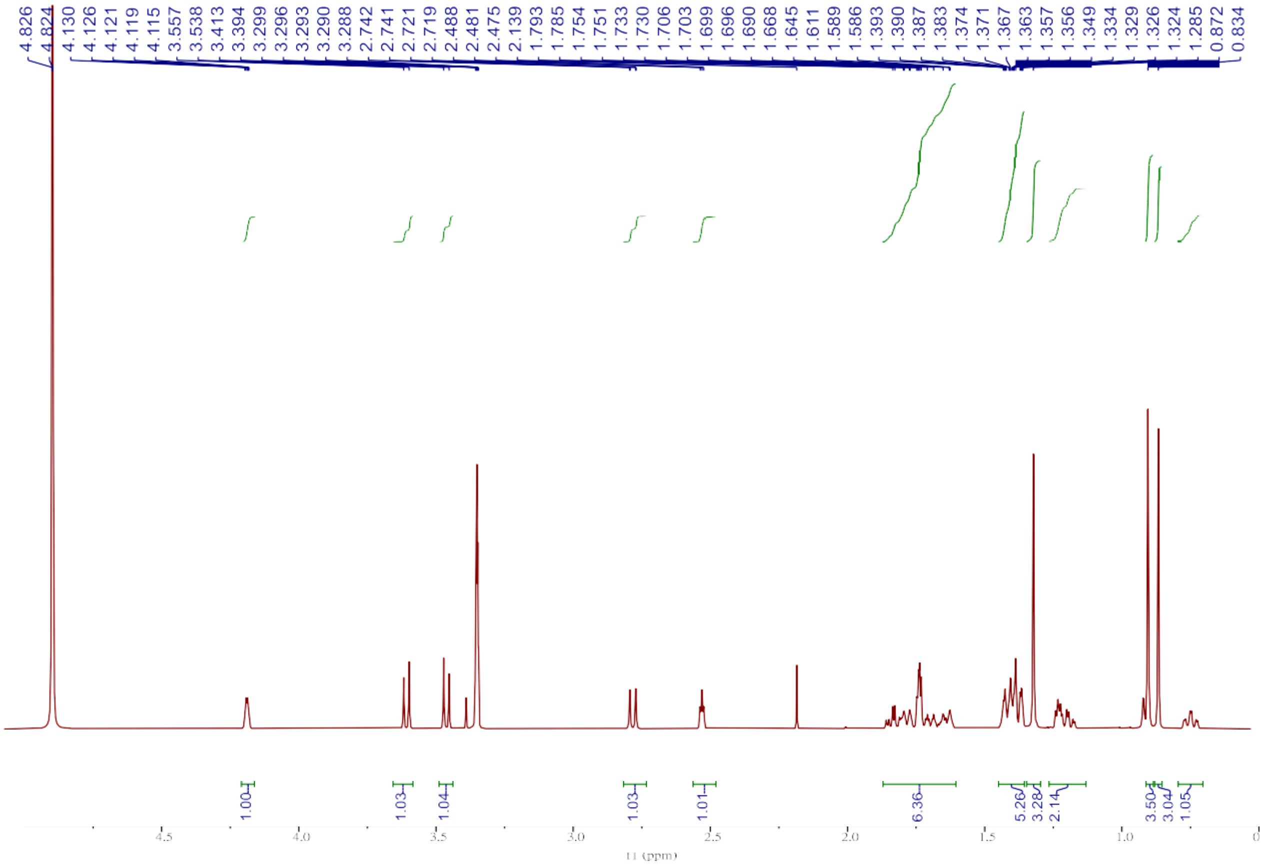

Figure S1. ^{1}H-NMR spectrum of mesonol A (**1**) in MeOD-*d*4 (600 MHz)

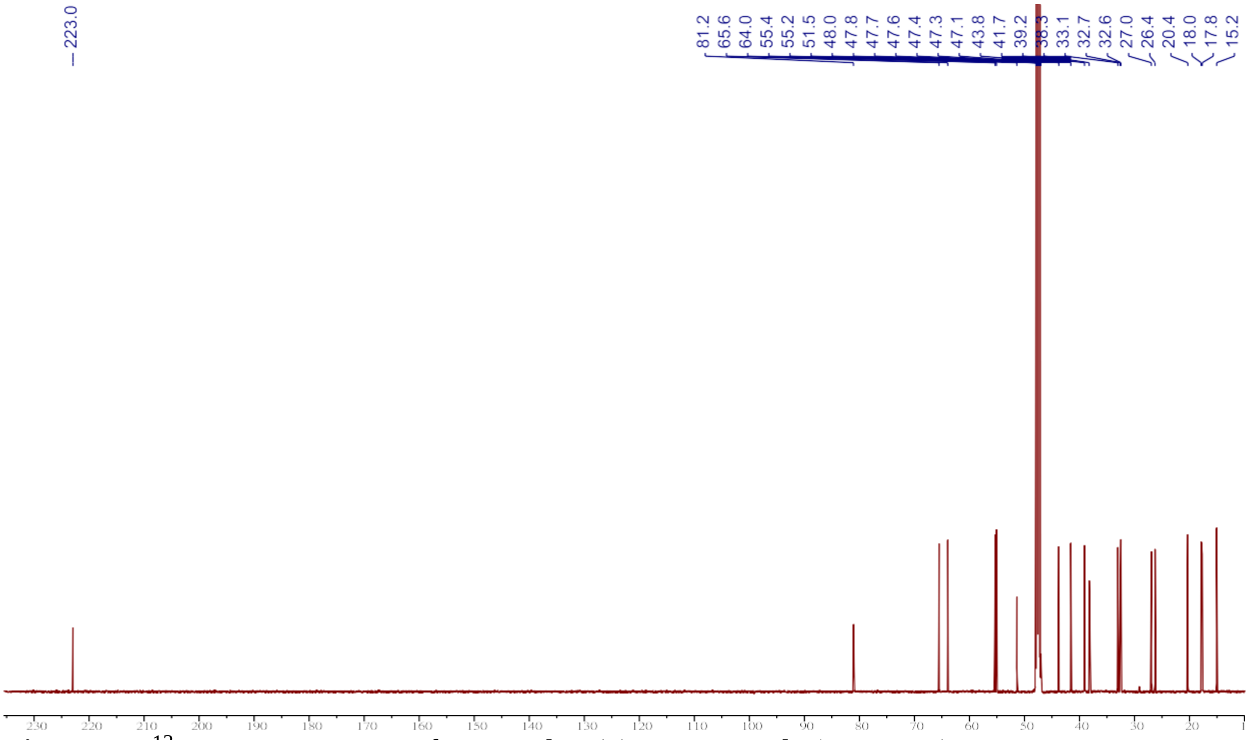

Figure S2. ^{13}C-NMR spectrum of mesonol A (**1**) in MeOD-*d*4 (150 MHz)

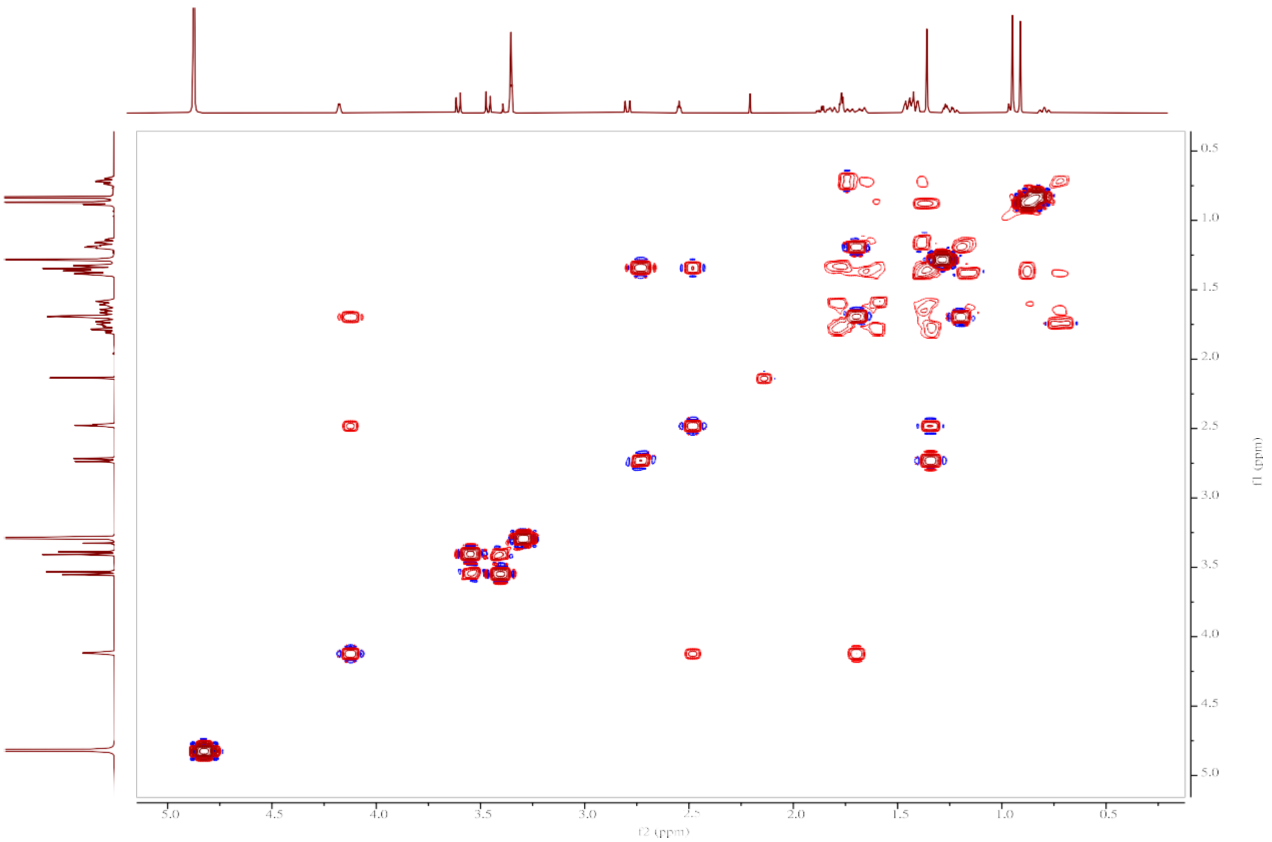

Figure S3. COSY spectrum of mesonol A (**1**)

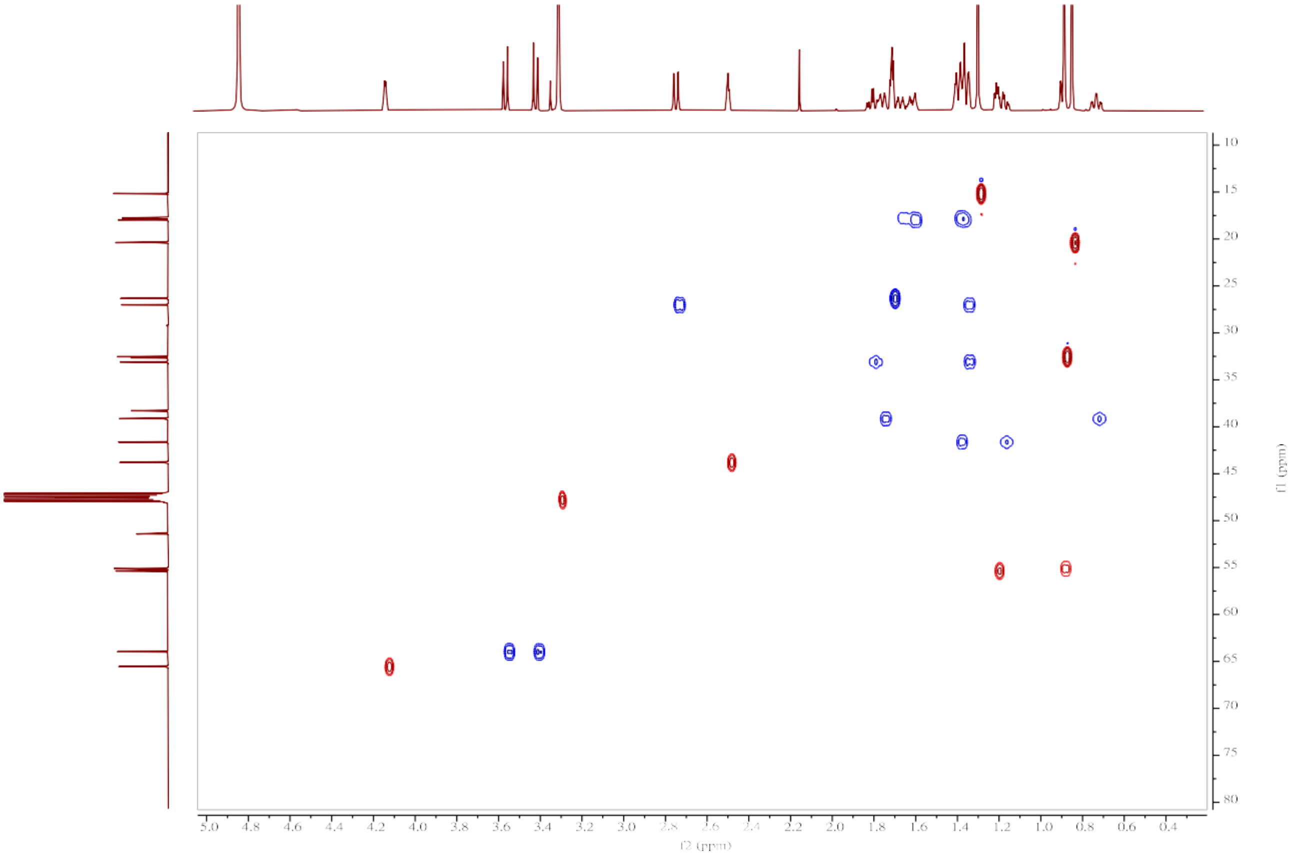

Figure S4. HSQC spectrum of mesonol A (**1**)

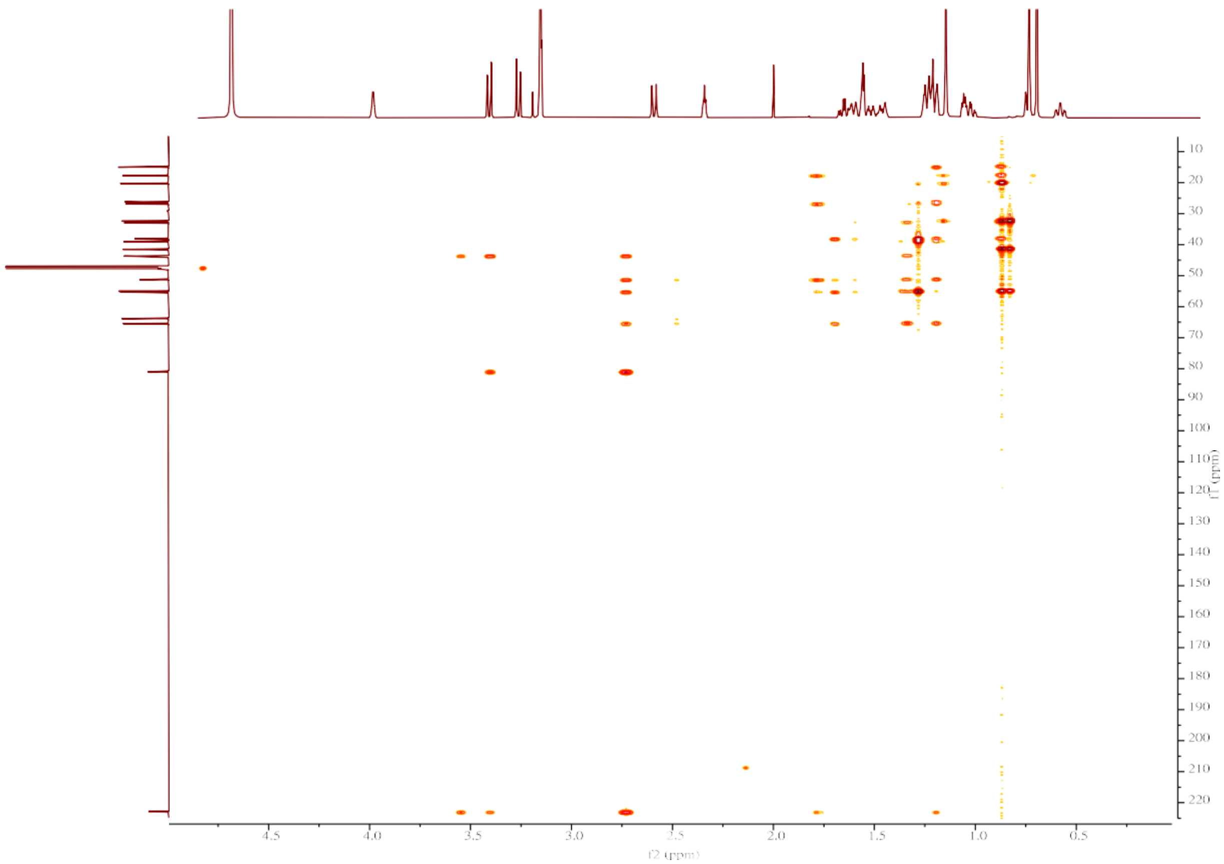

Figure S5. HMBC spectrum of mesonol A (**1**)

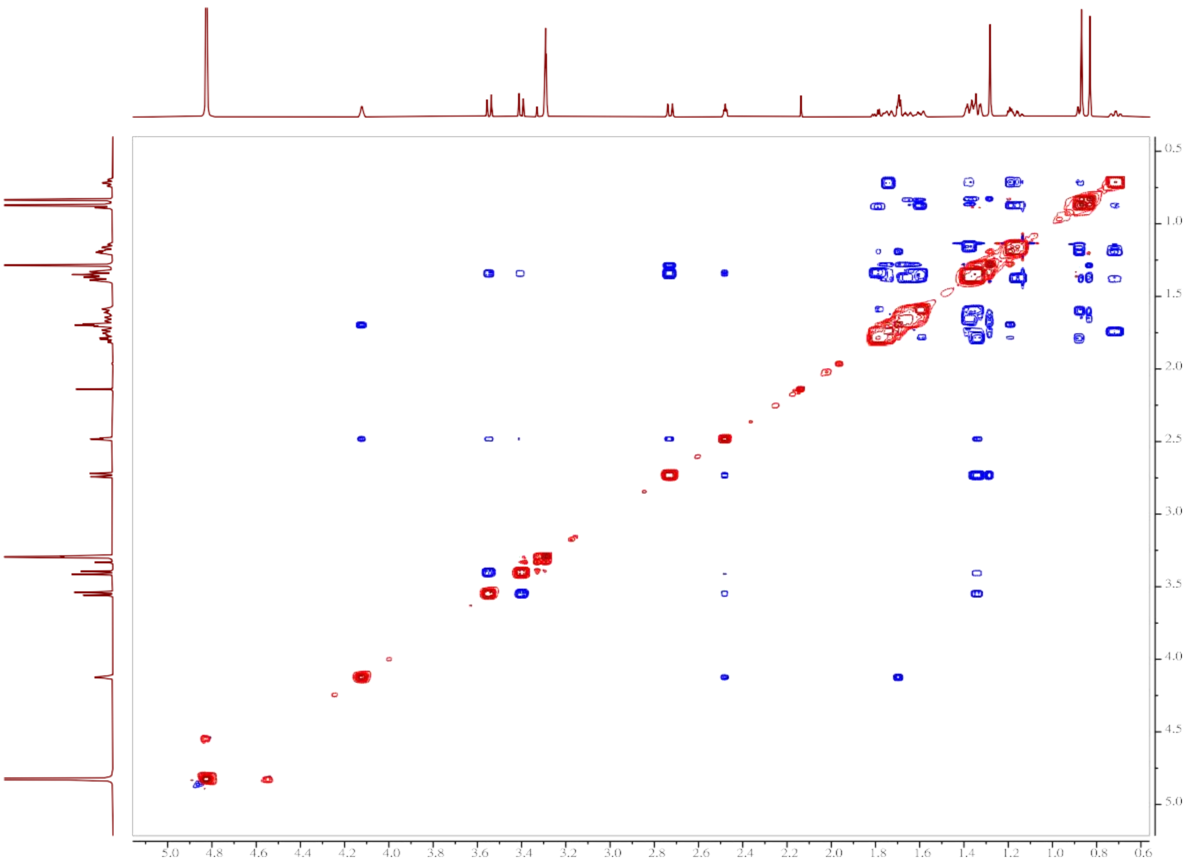

Figure S6. NOESY spectrum of mesonol A (**1**)

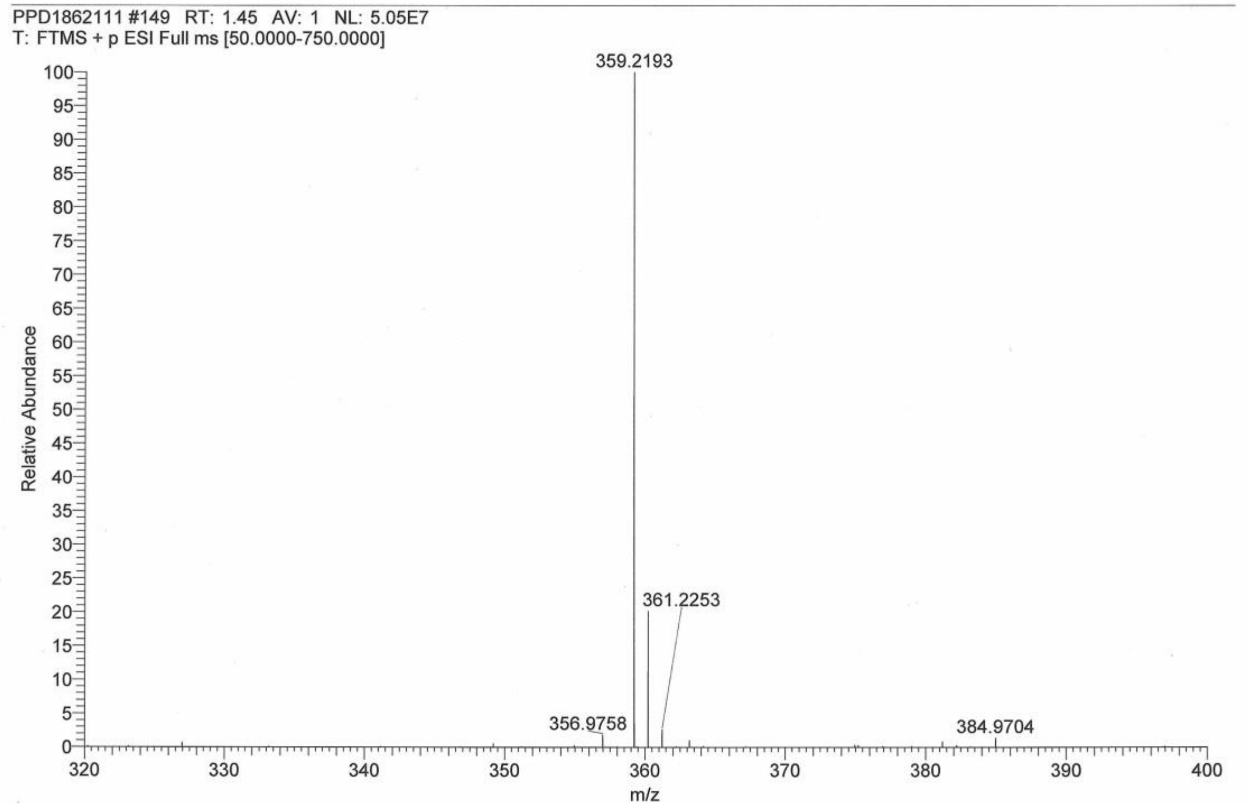

Figure S7. (+)-HRESIMS spectrum of mesonol A (**1**)

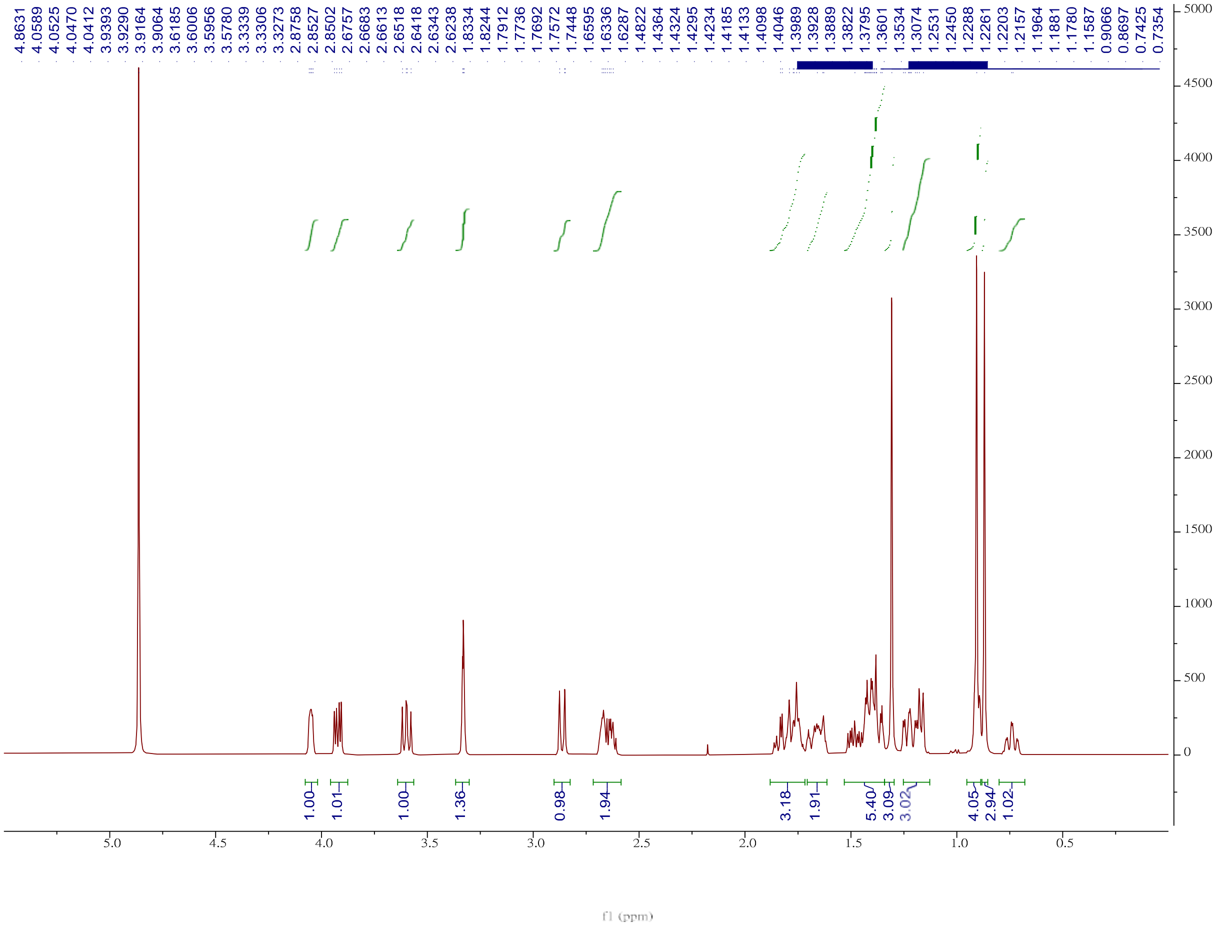

Figure S8. ^{1}H-NMR spectrum of mesonol B (**2**) in MeOD-*d*4 (500 MHz)

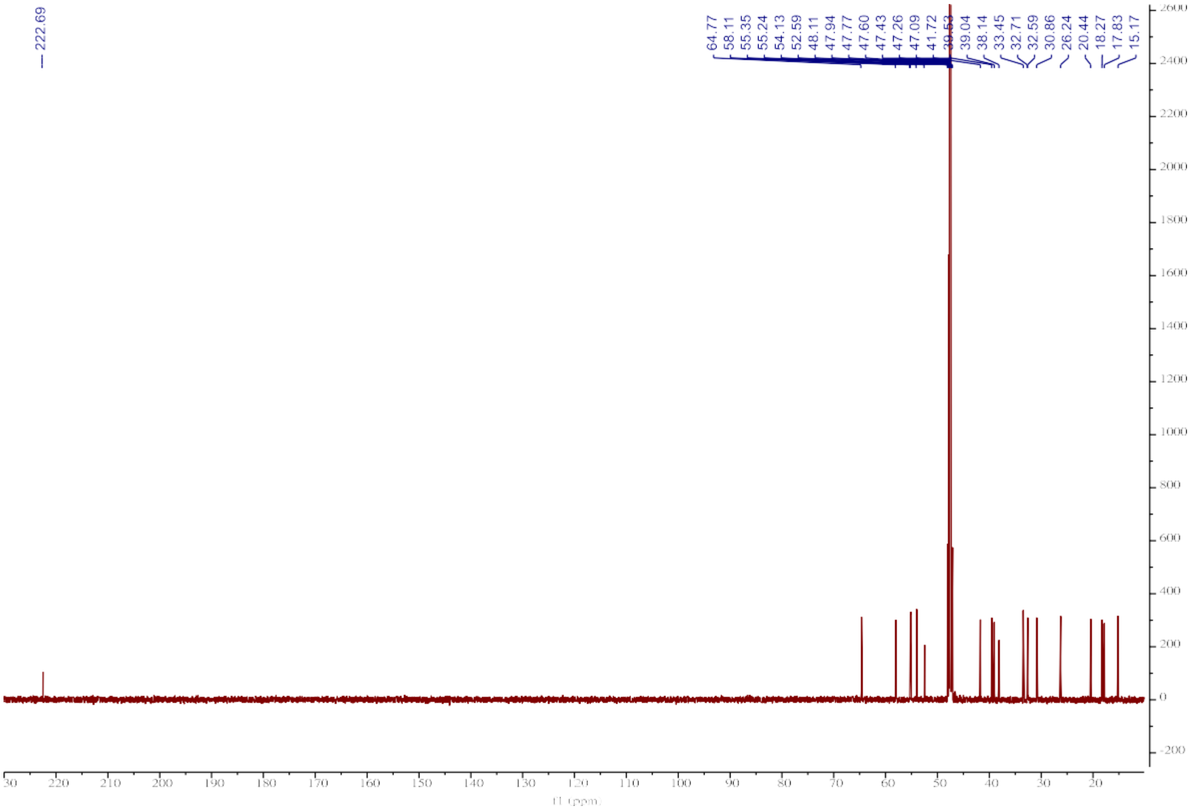

Figure S9. ^{13}C-NMR spectrum of mesonol B (**2**) in MeOD-*d*4 (125 MHz)

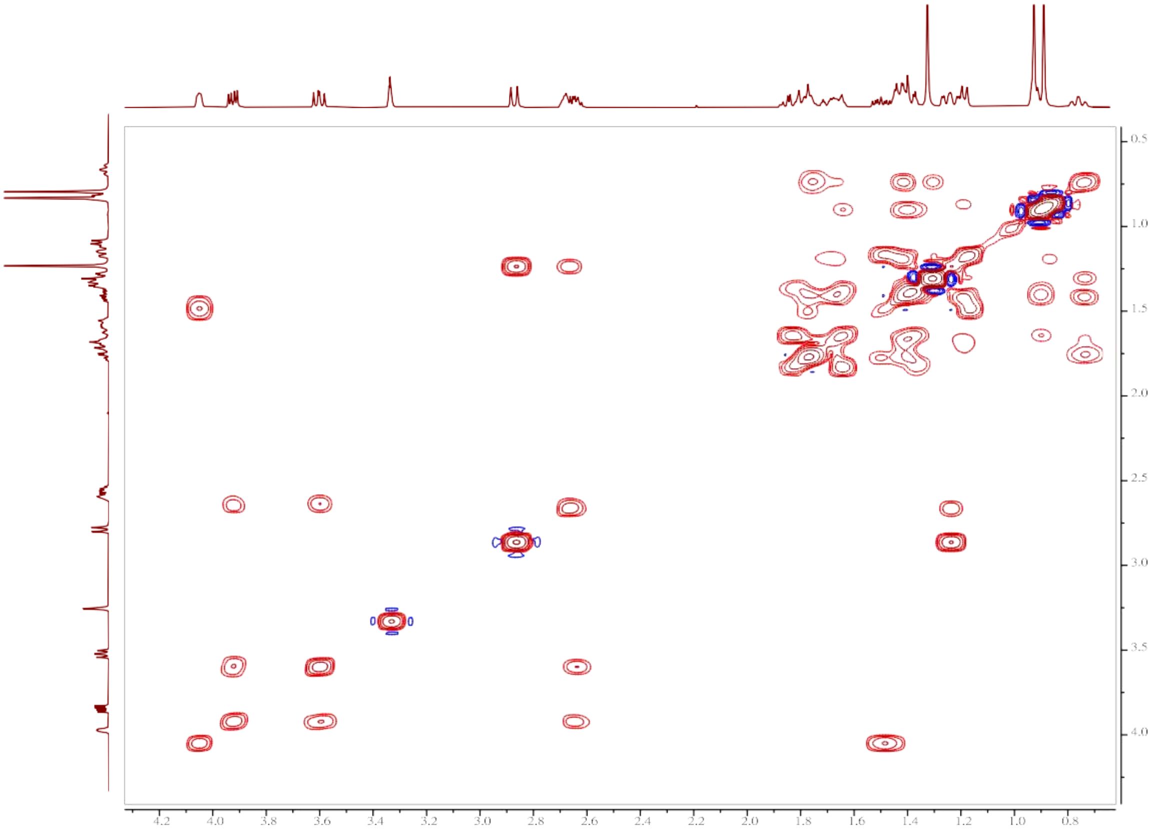

Figure S10. COSY spectrum of mesonol B (**2**)

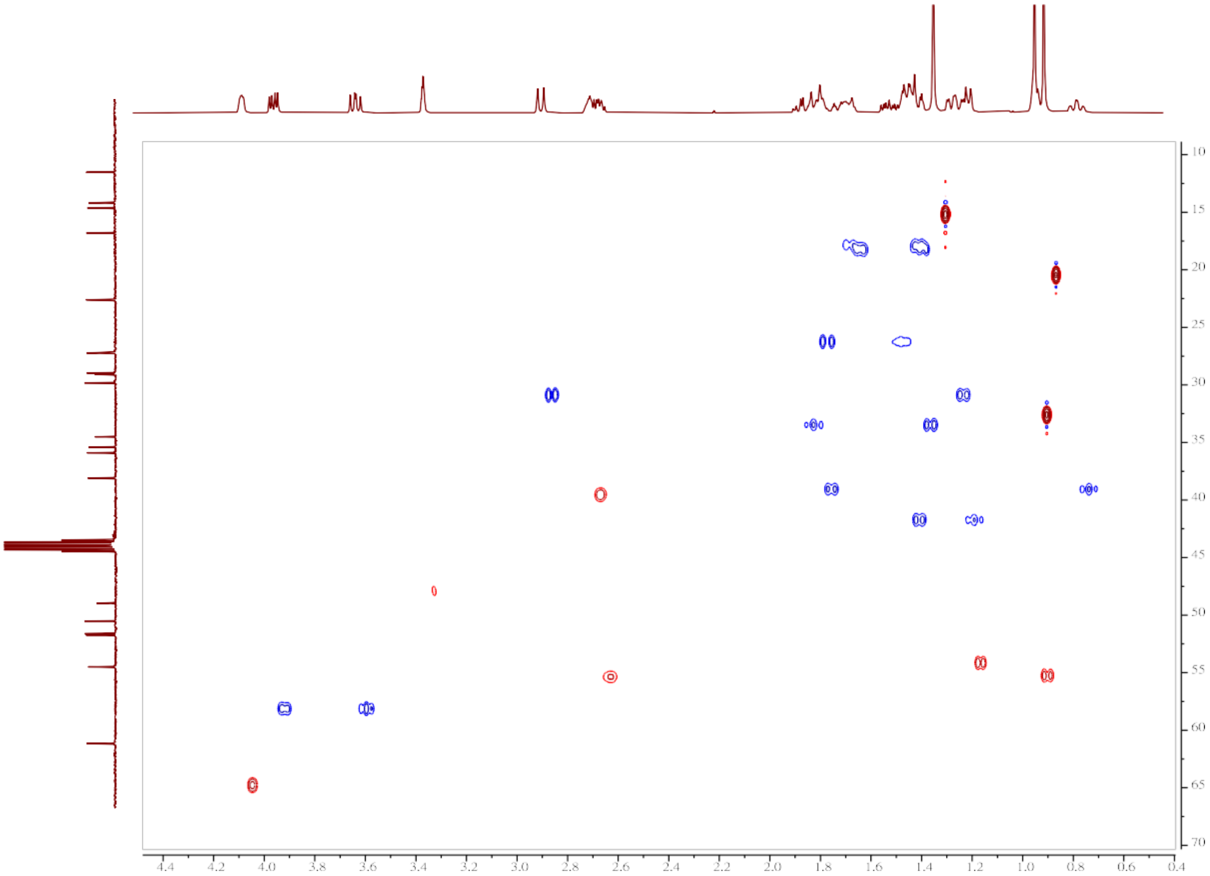

Figure S11. HSQC spectrum of mesonol B (**2**)

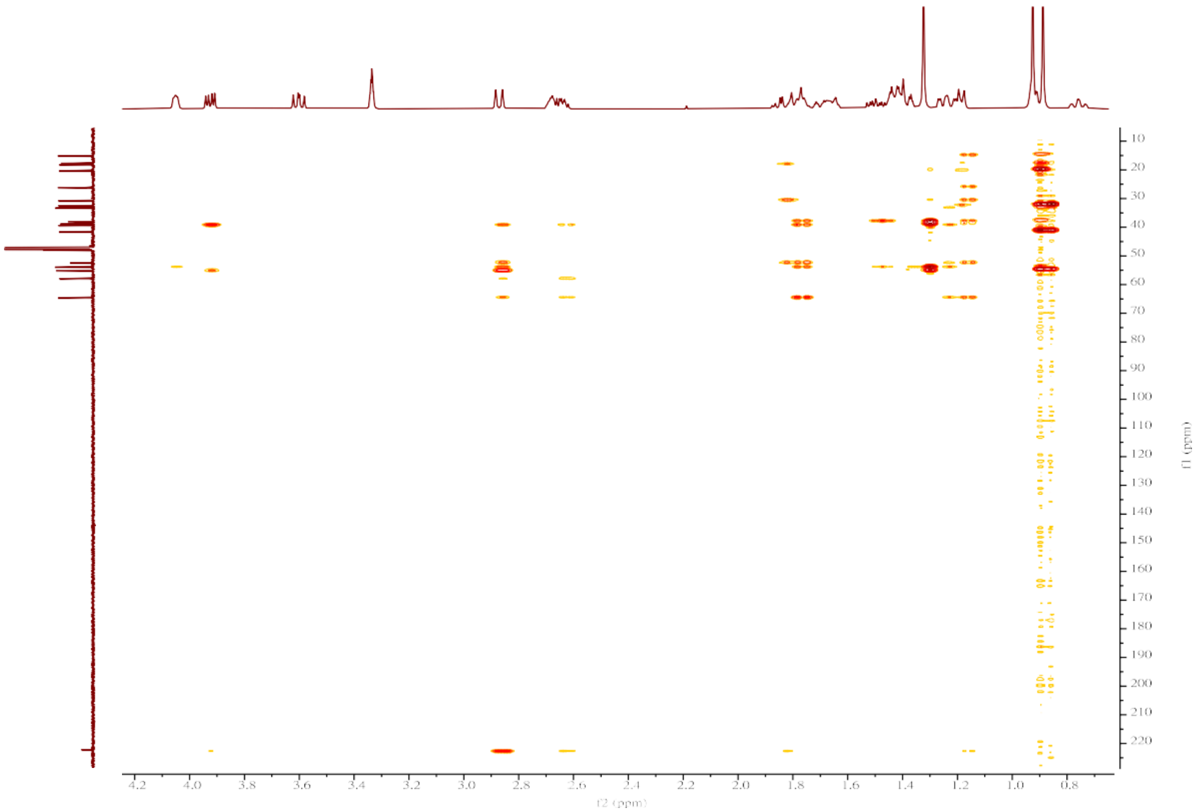

Figure S12. HMBC spectrum of mesonol B (**2**)

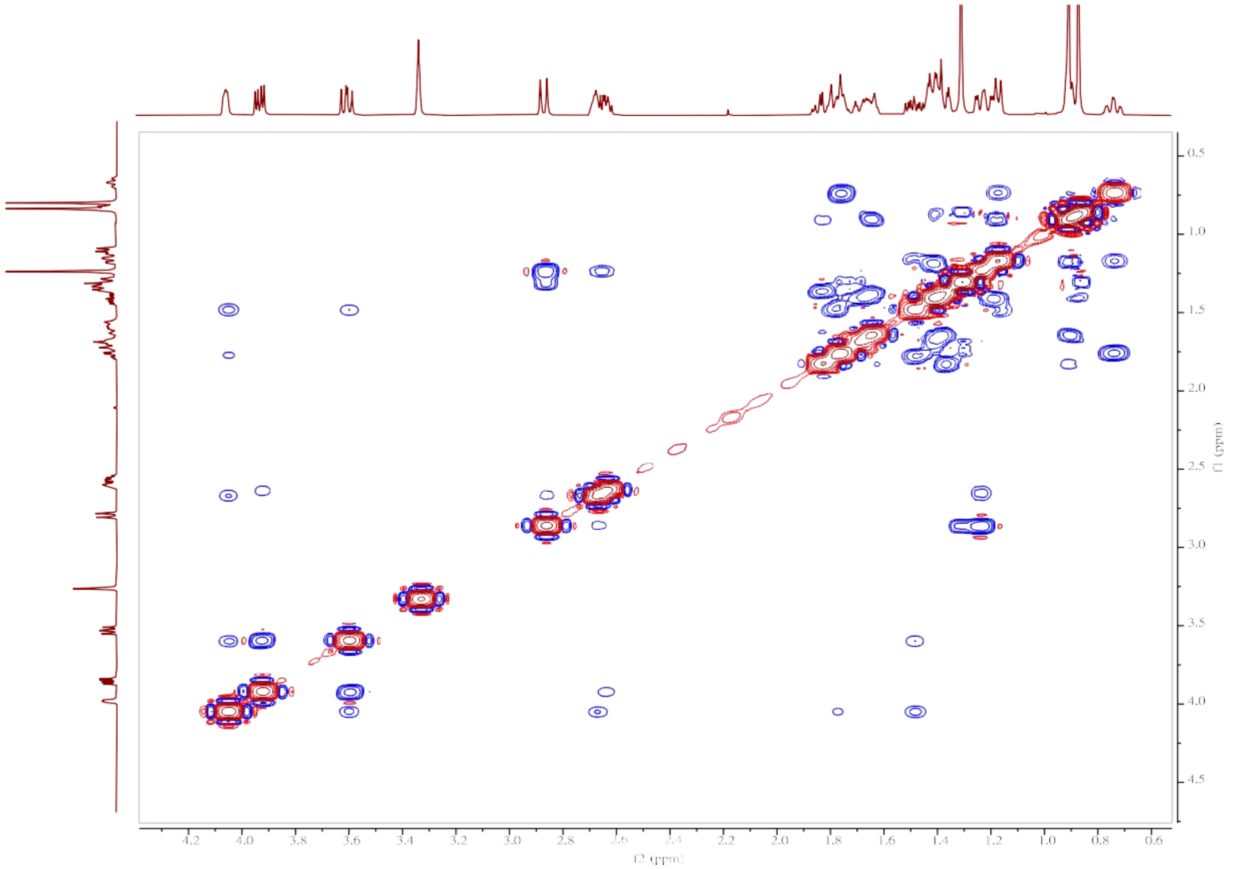

Figure S13. NOESY spectrum of mesonol B (**2**)

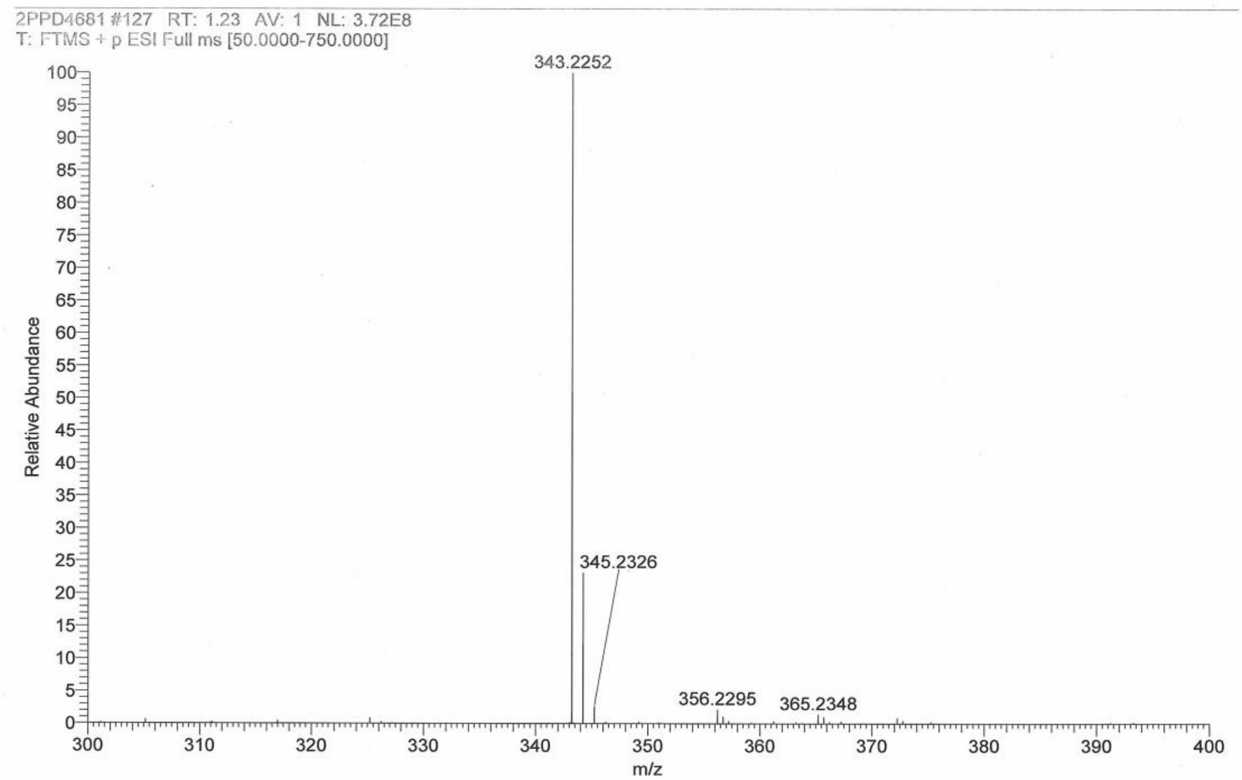

Figure S14. (+)-HRESIMS spectrum of mesonol B (**2**)

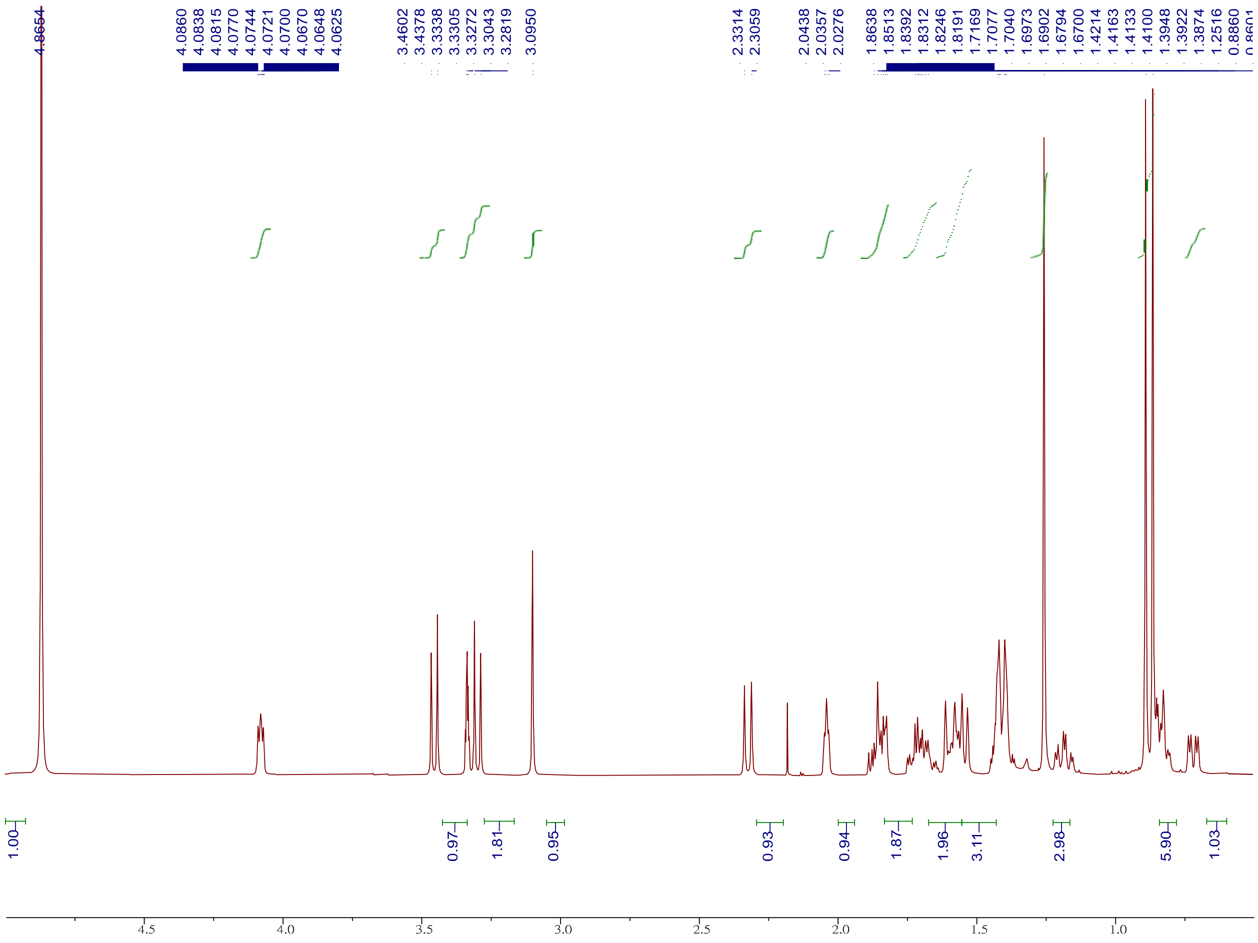

Figure S15. ^{1}H-NMR spectrum of mesonol C (**3**) in MeOD-*d*4 (500 MHz)

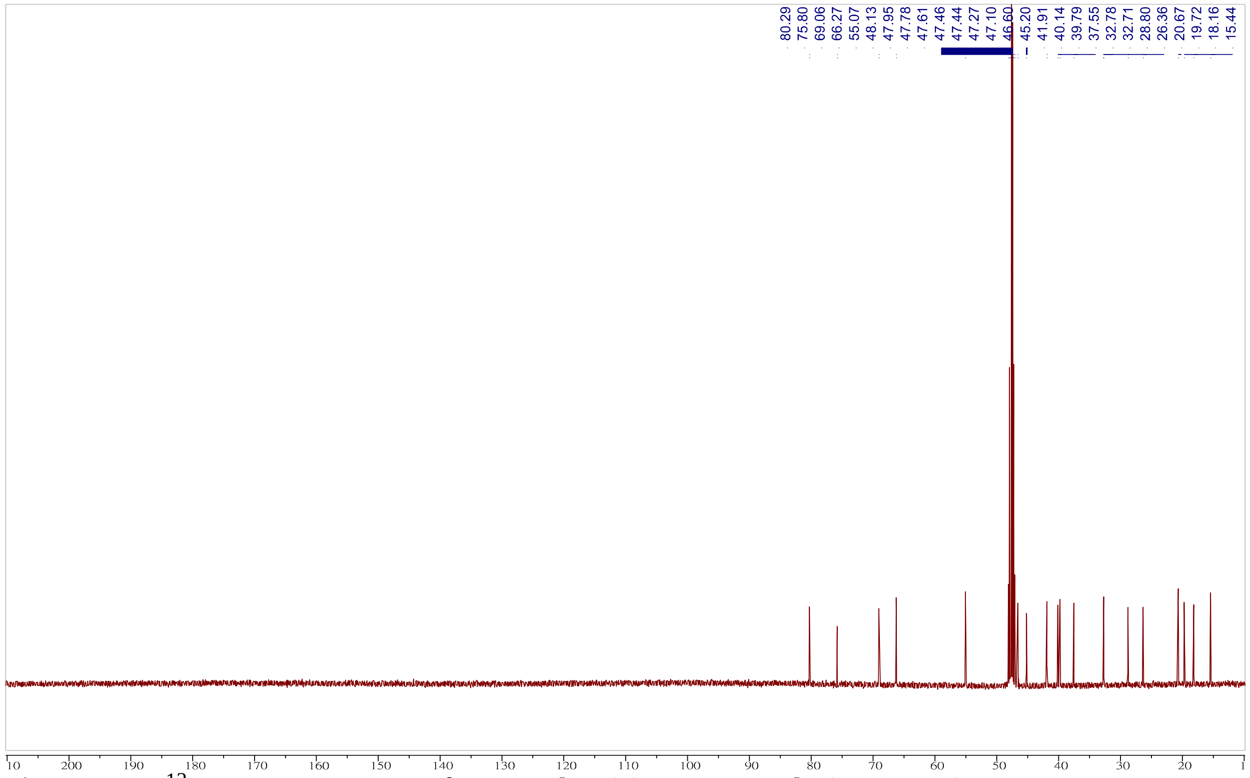

Figure S16. ^{13}C-NMR spectrum of mesonol C (**3**) in MeOD-*d*4 (125 MHz)

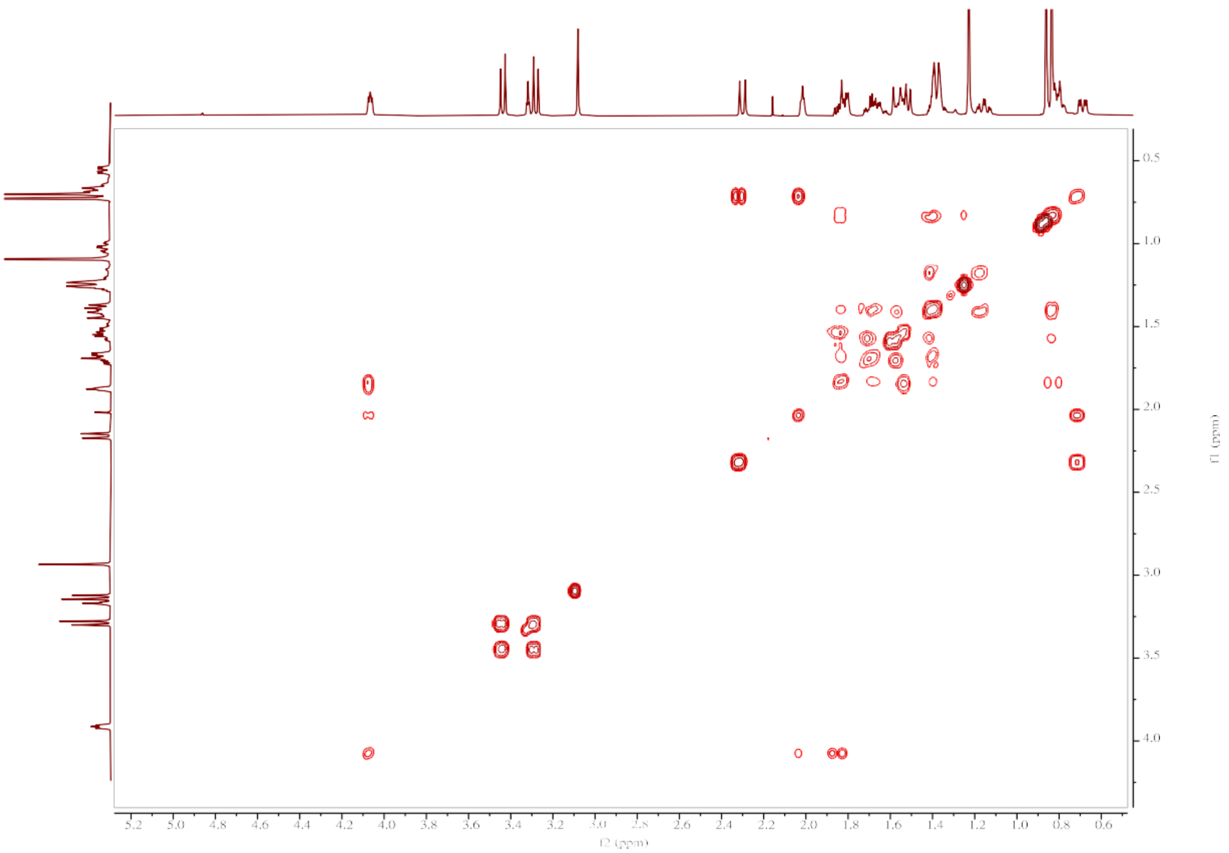

Figure S17. COSY spectrum of mesonol C (**3**)

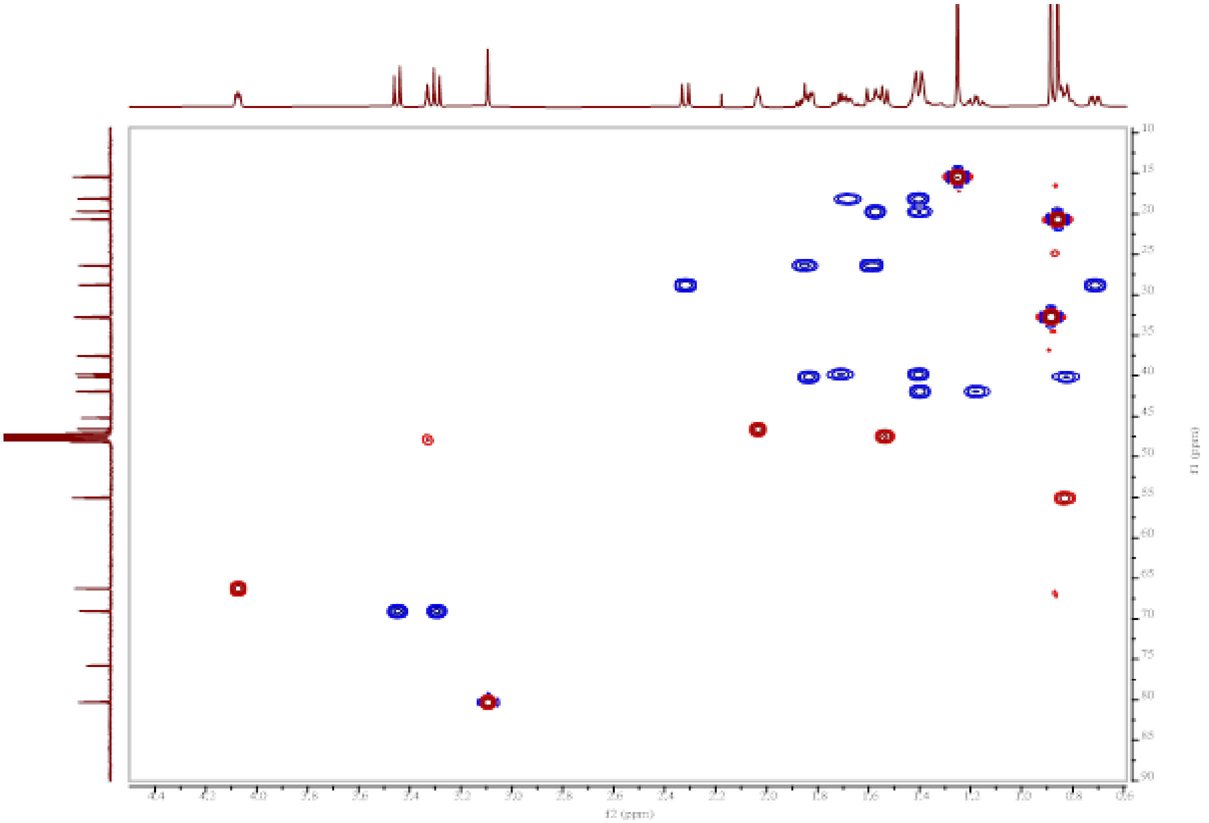

Figure S18. HSQC spectrum of mesonol C (**3**)

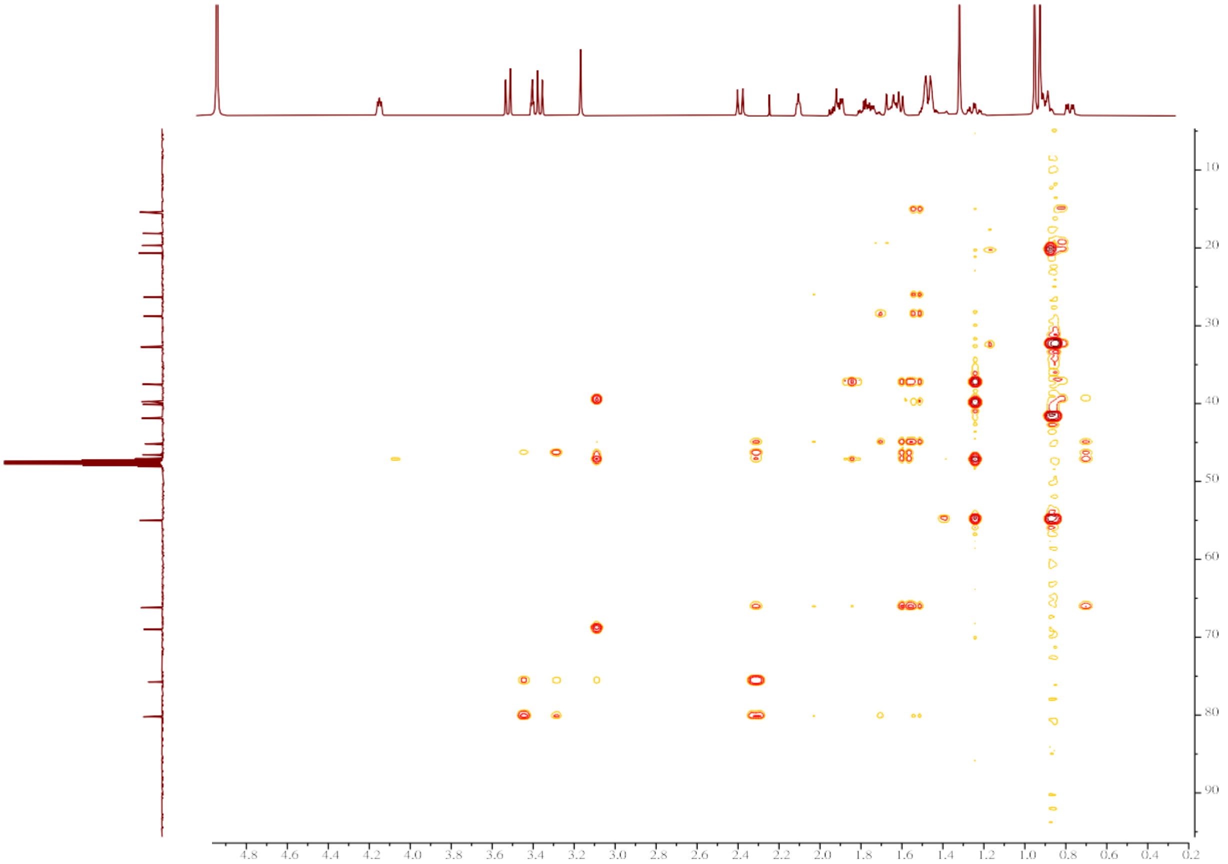

Figure S19. HMBC spectrum of mesonol C (**3**)

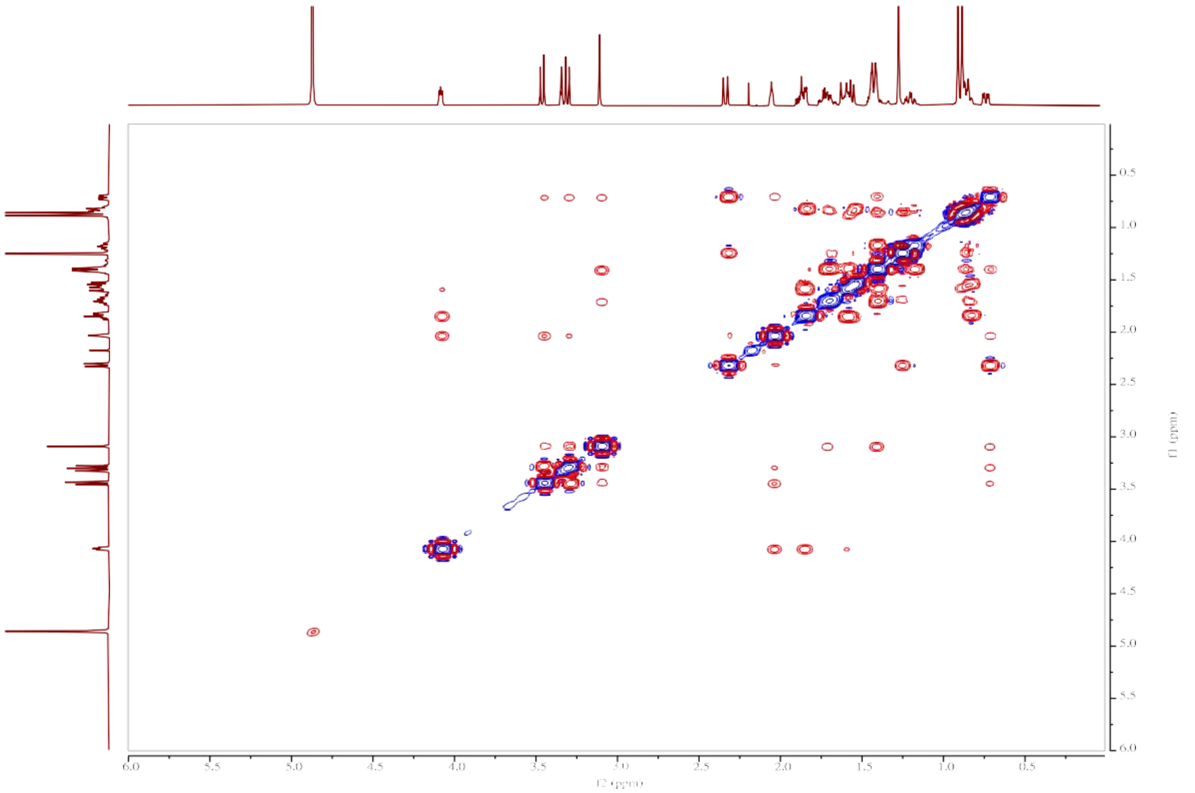

Figure S20. NOESY spectrum of mesonol C (**3**)

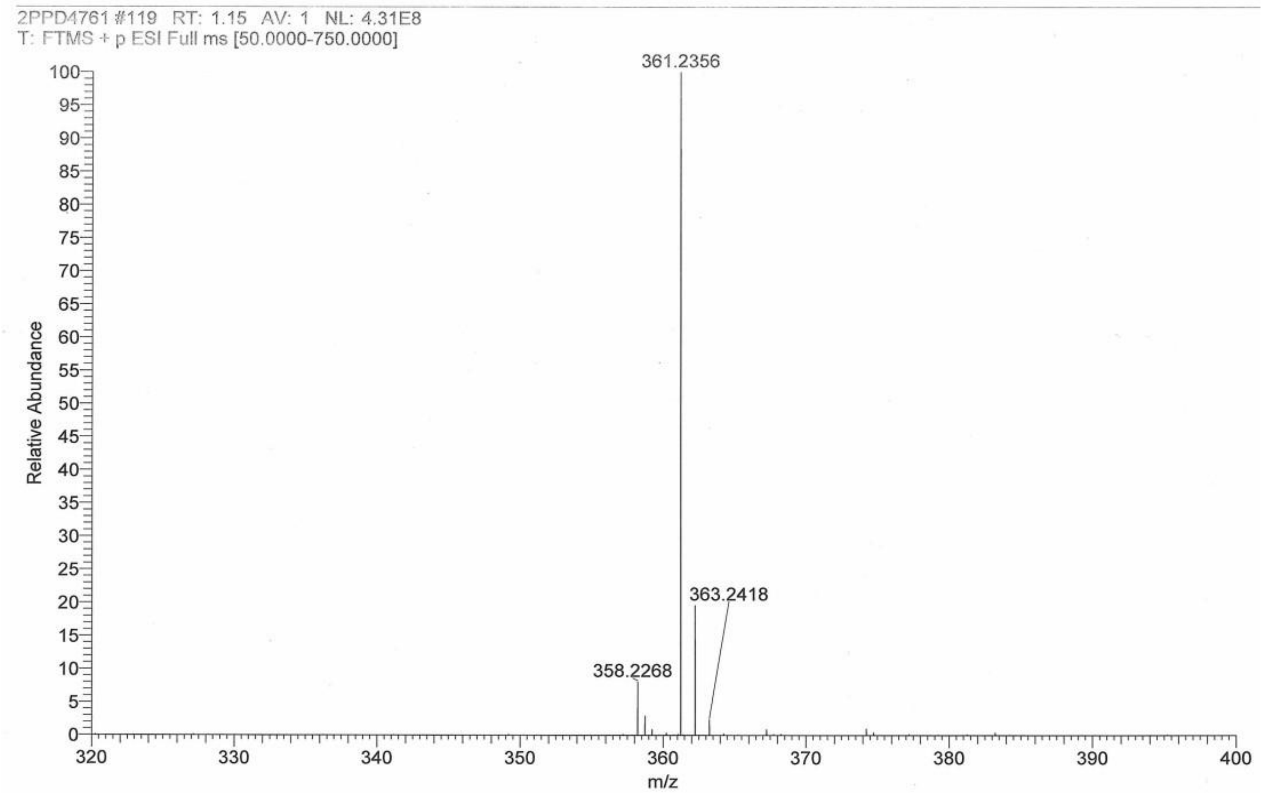

Figure S21. (+)-HRESIMS spectrum of mesonol C (**3**)

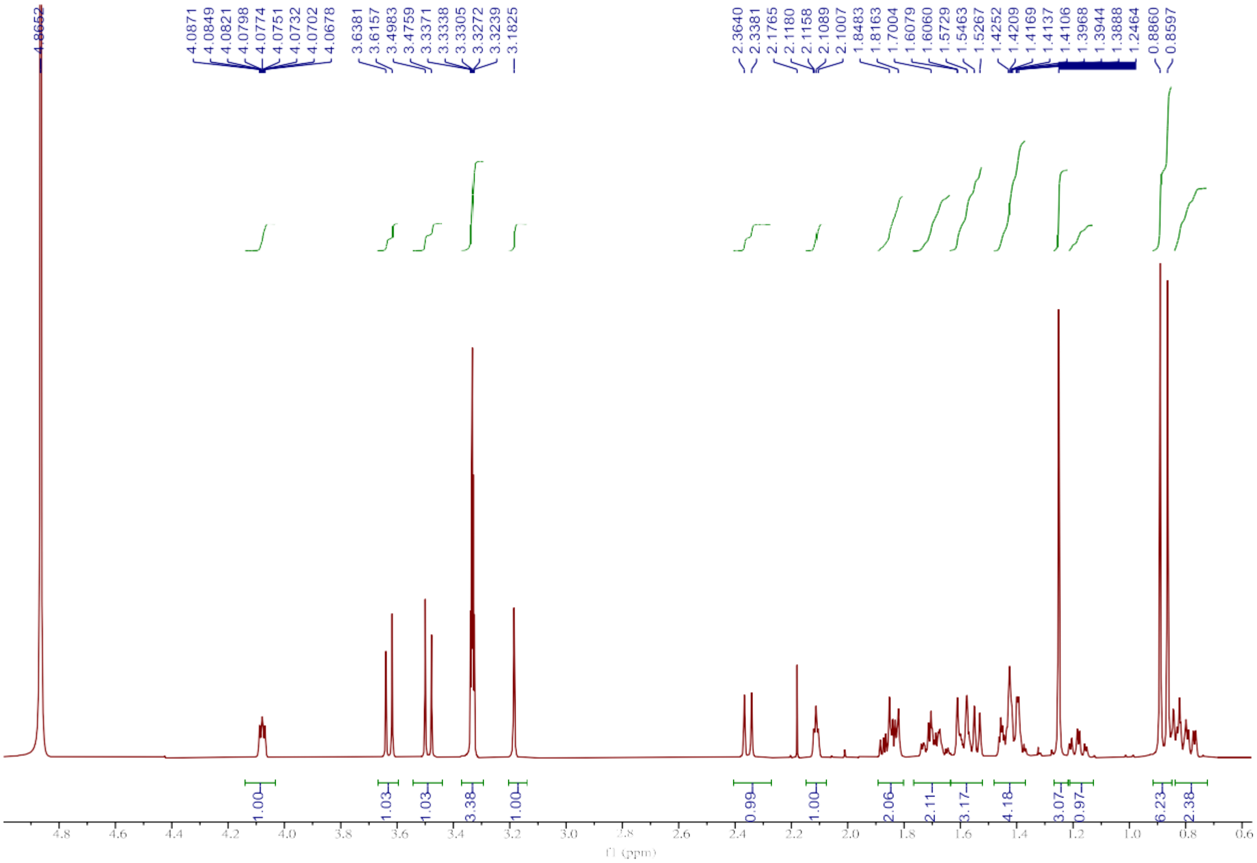

Figure S22. ^{1}H-NMR spectrum of mesonol D (**4**) in MeOD-*d*4 (500 MHz)

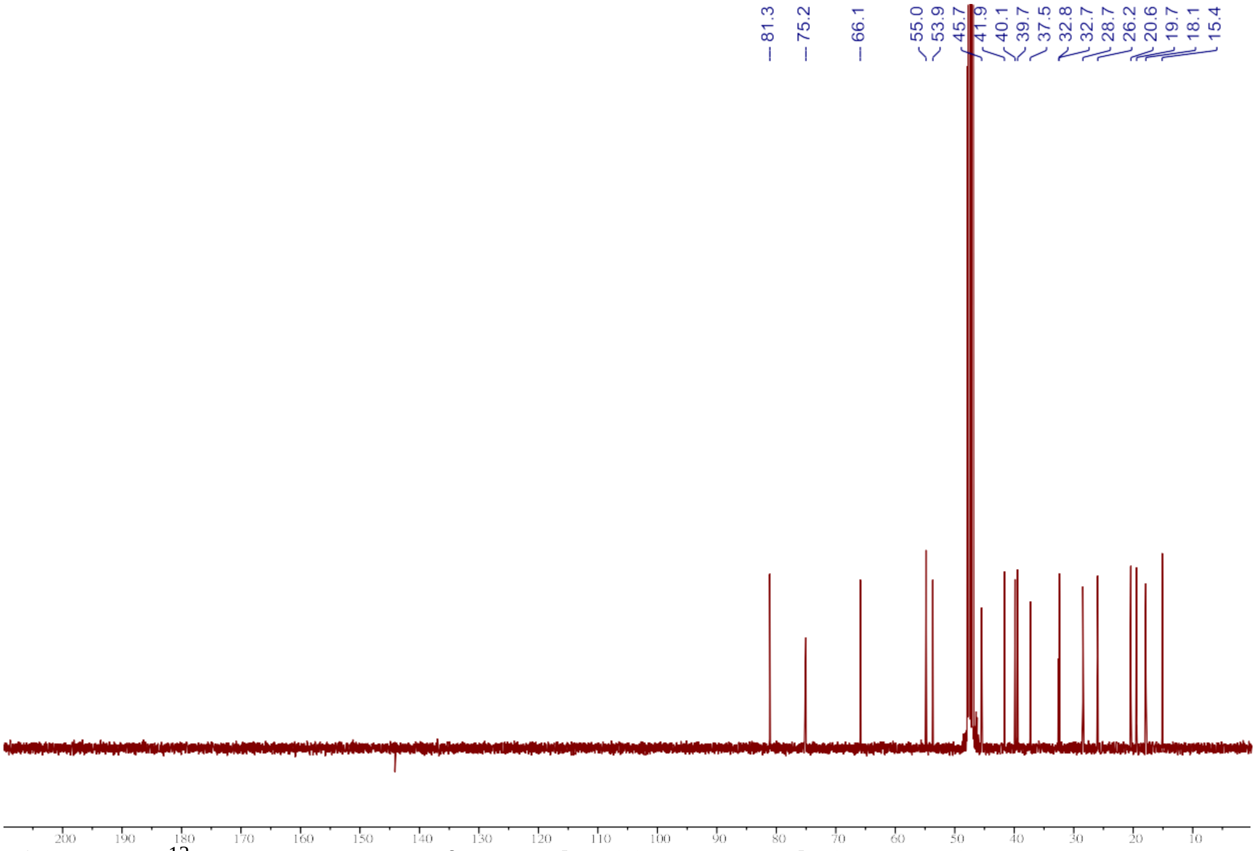

Figure S23. ^{13}C-NMR spectrum of mesonol D (**4**) in MeOD-*d*4 (125 MHz)

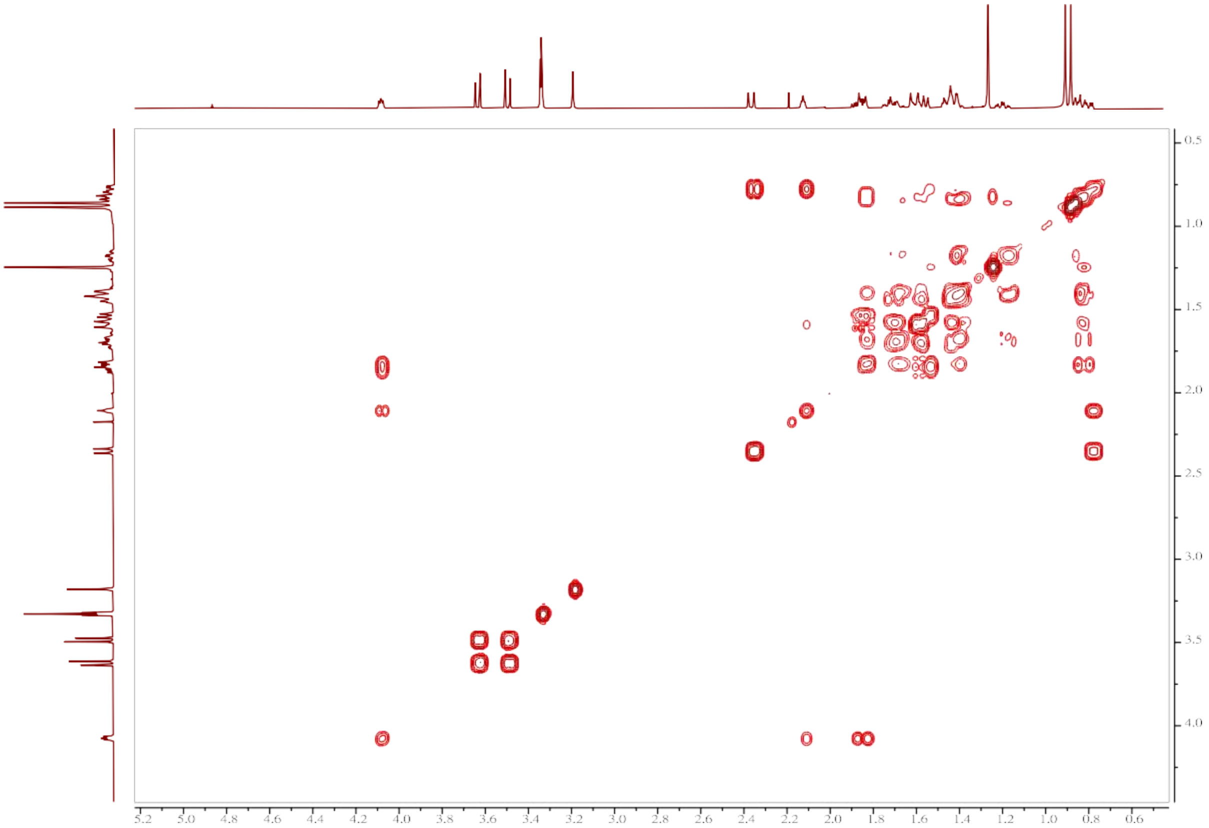

Figure S24. COSY spectrum of mesonol D (**4**)

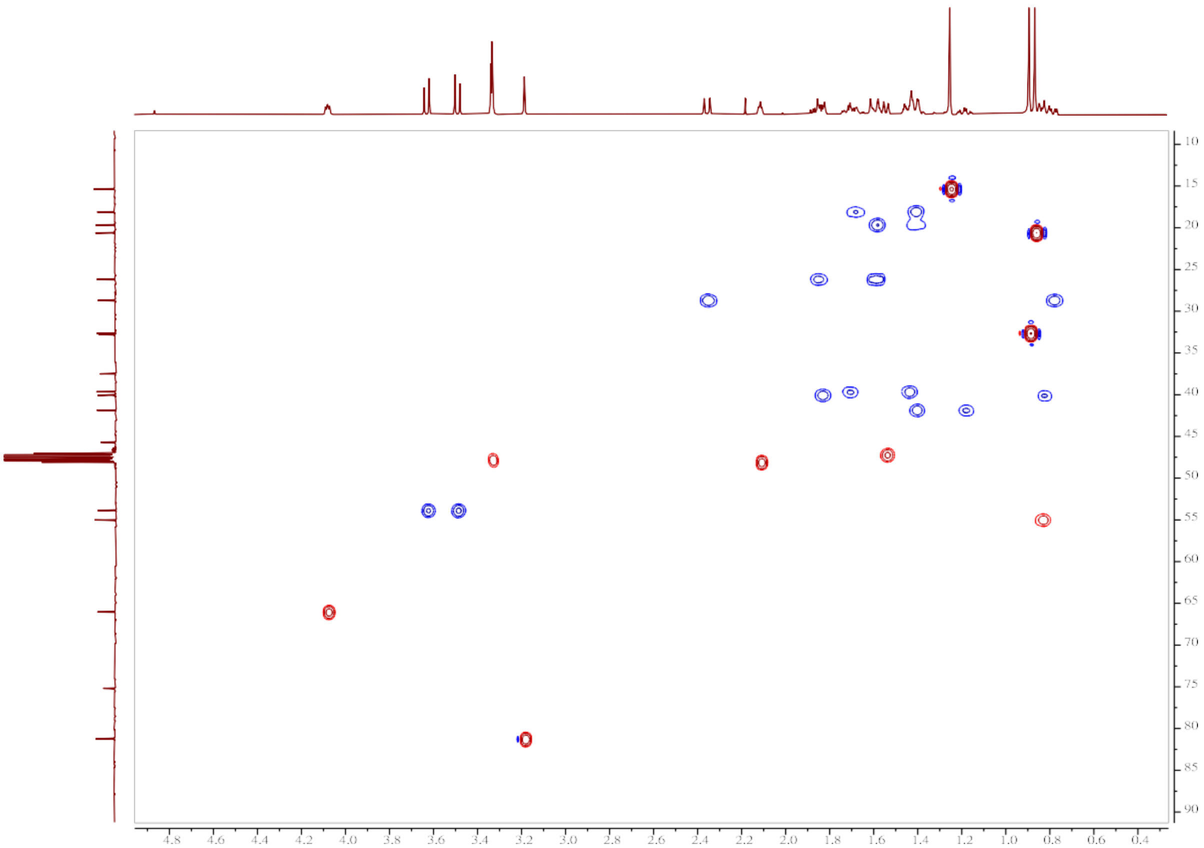

Figure S25. HSQC spectrum of mesonol D (**4**)

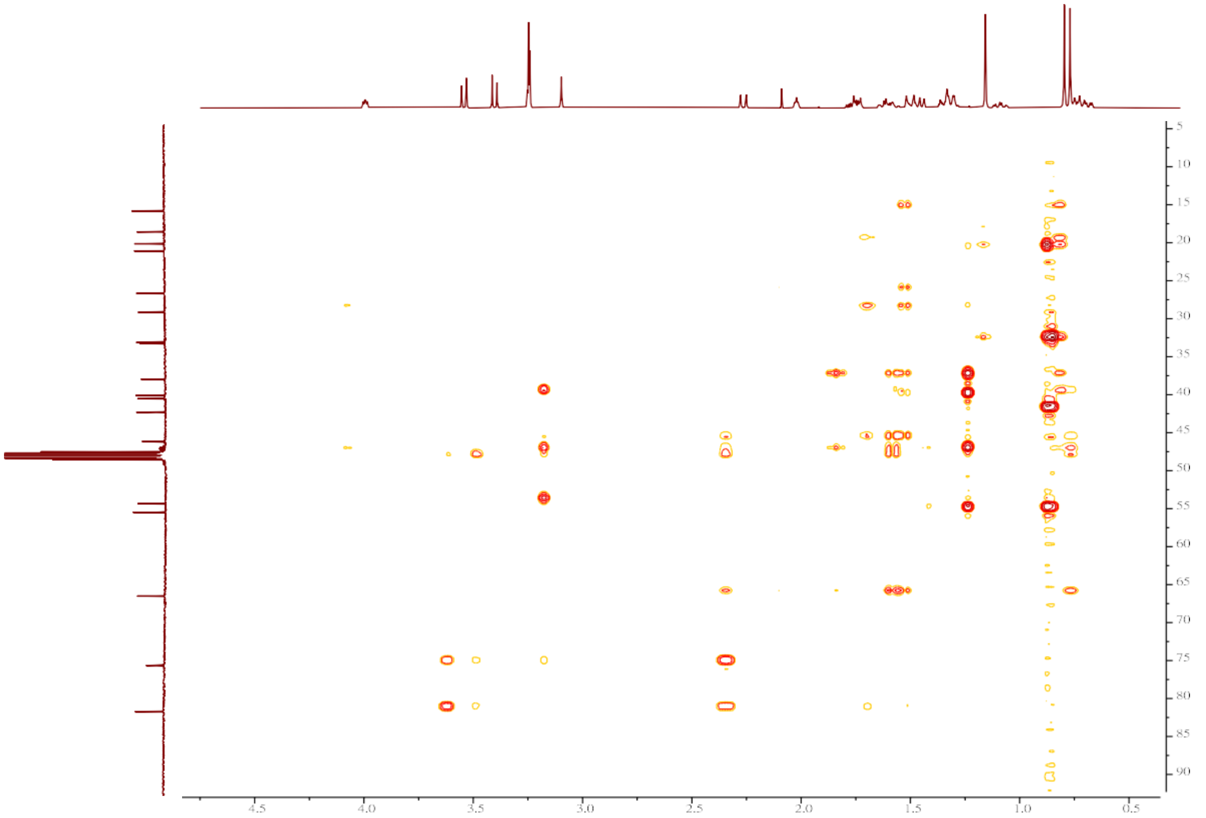

Figure S26. HMBC spectrum of mesonol D (**4**)

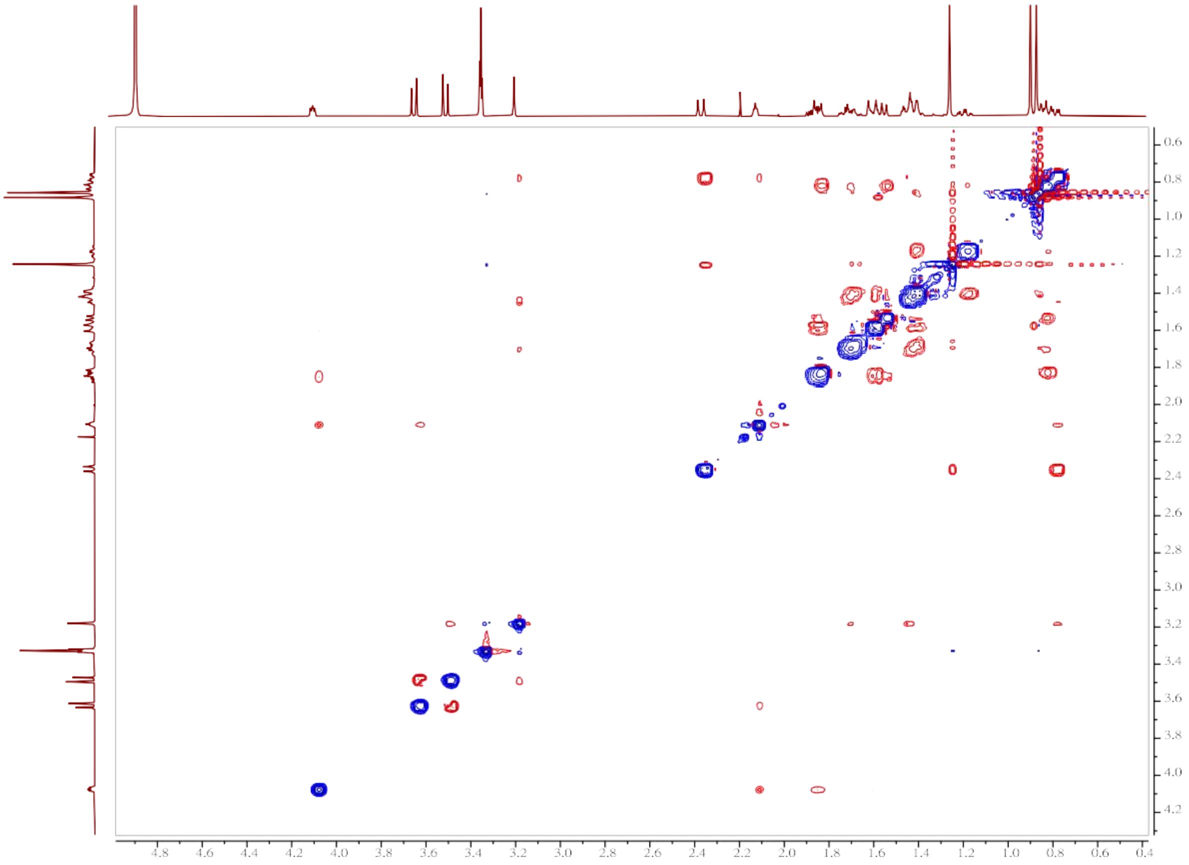

Figure S27. NOESY spectrum of mesonol D (**4**)

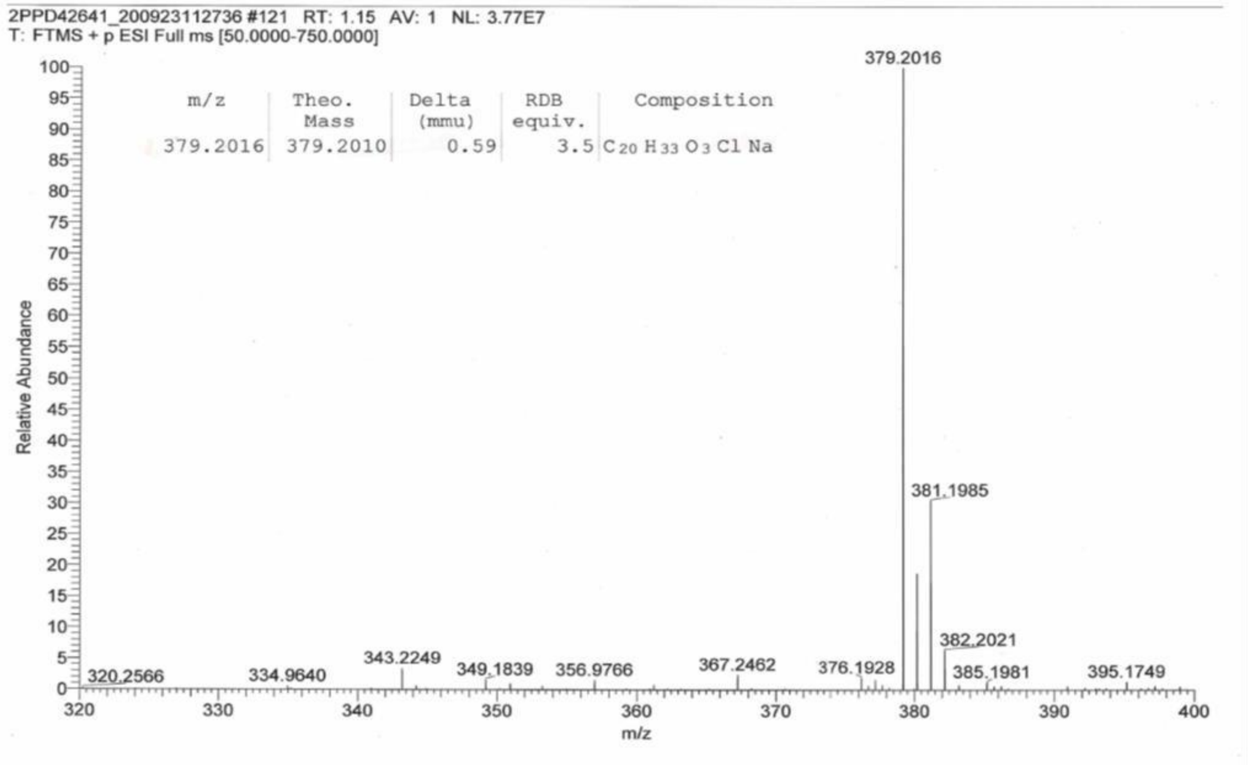

Figure S28. (+)-HRESIMS spectrum of mesonol D (**4**)

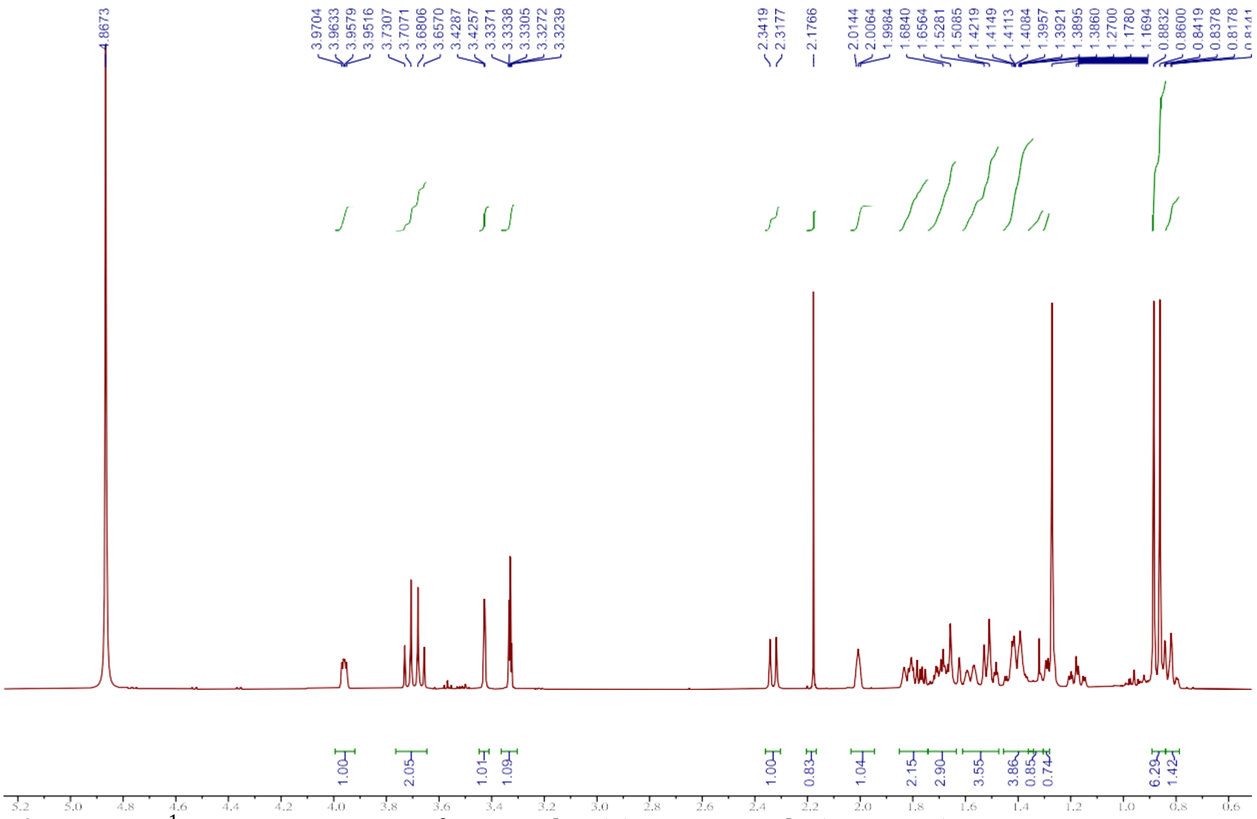

Figure S29. ^{1}H-NMR spectrum of mesonol E (**5**) in MeOD-*d*4 (500 MHz)

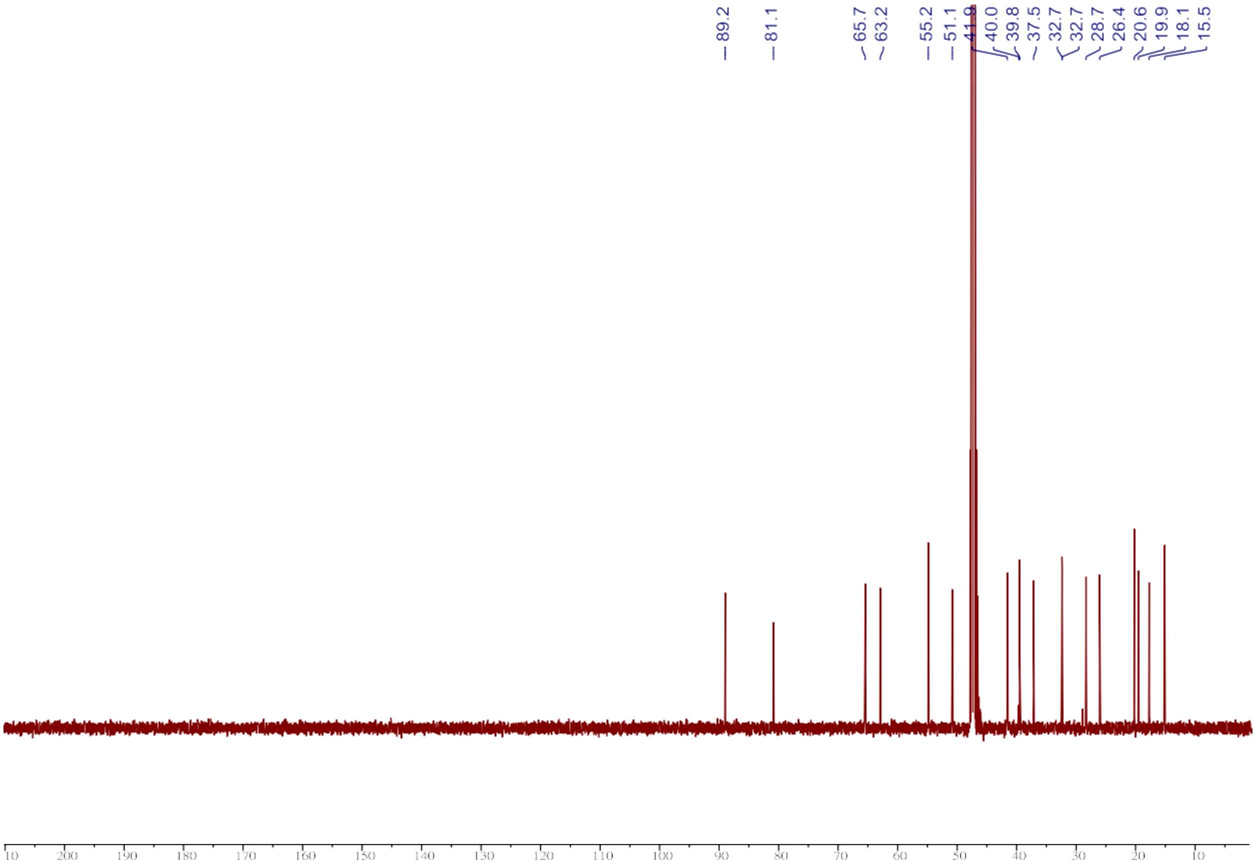

Figure S30. ^{13}C-NMR spectrum of mesonol E (**5**) in MeOD-*d*4 (125 MHz)

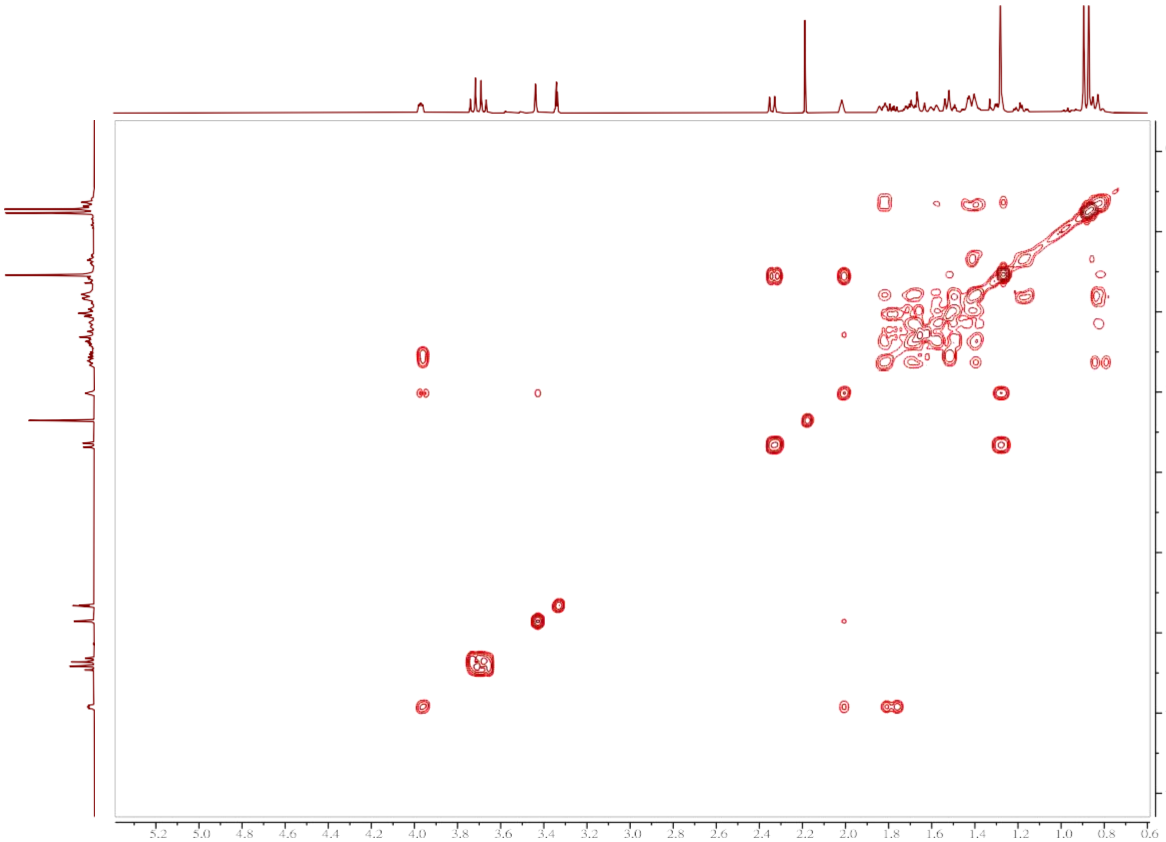

Figure S31. COSY spectrum of mesonol E (**5**)

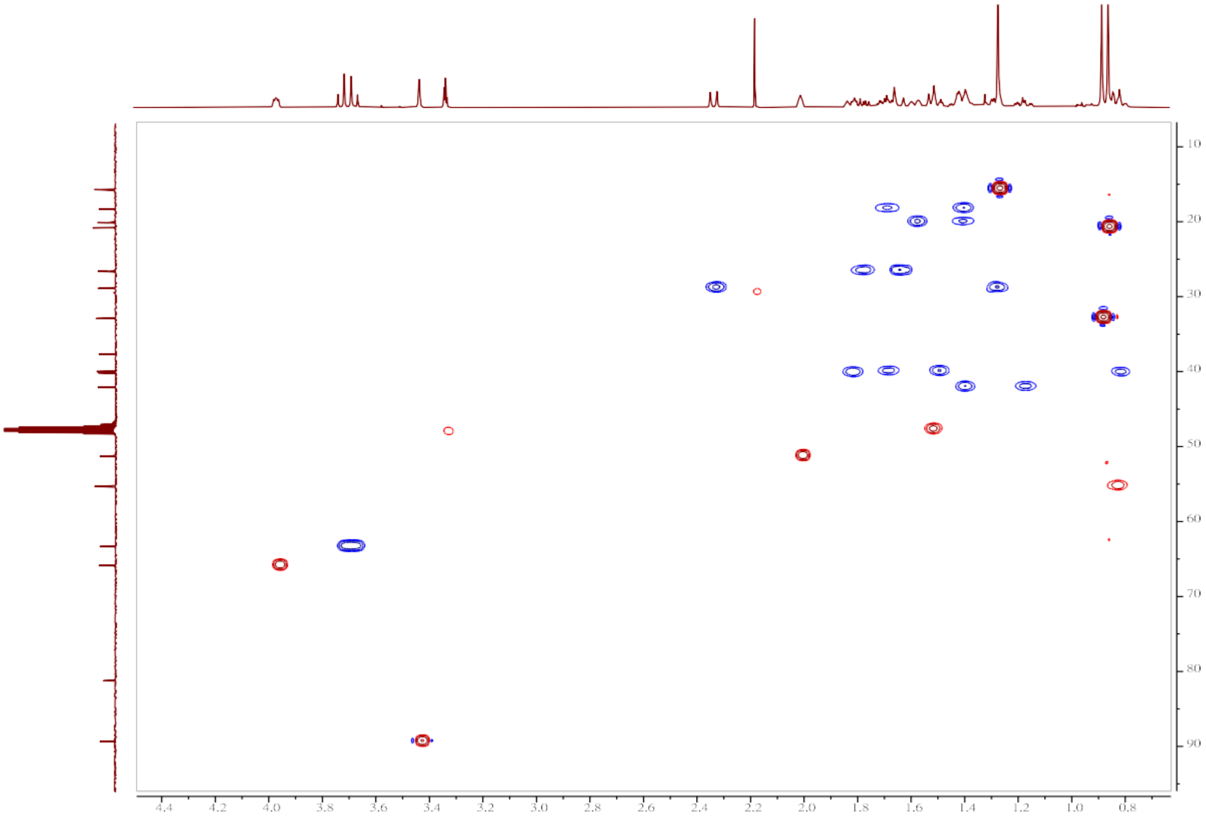

Figure S32. HSQC spectrum of mesonol E (**5**)

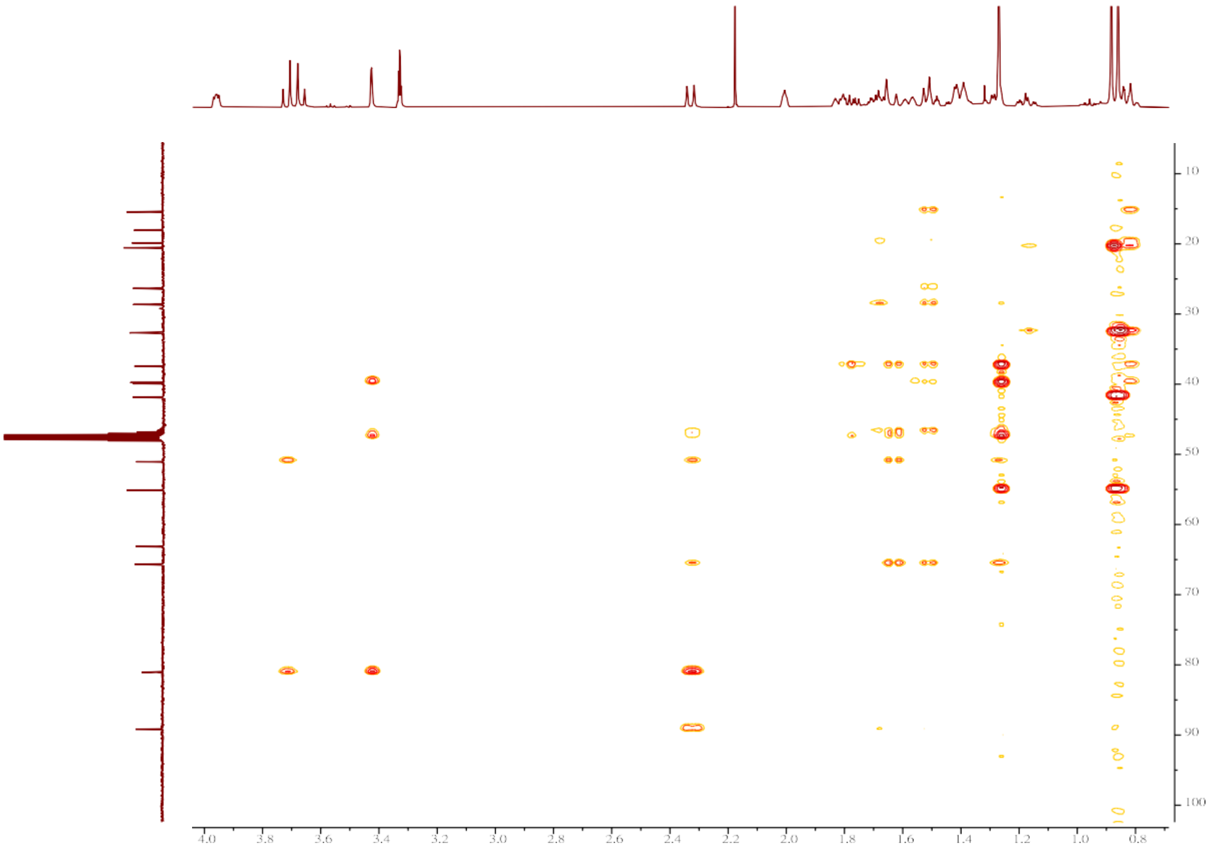

Figure S33. HMBC spectrum of mesonol E (**5**)

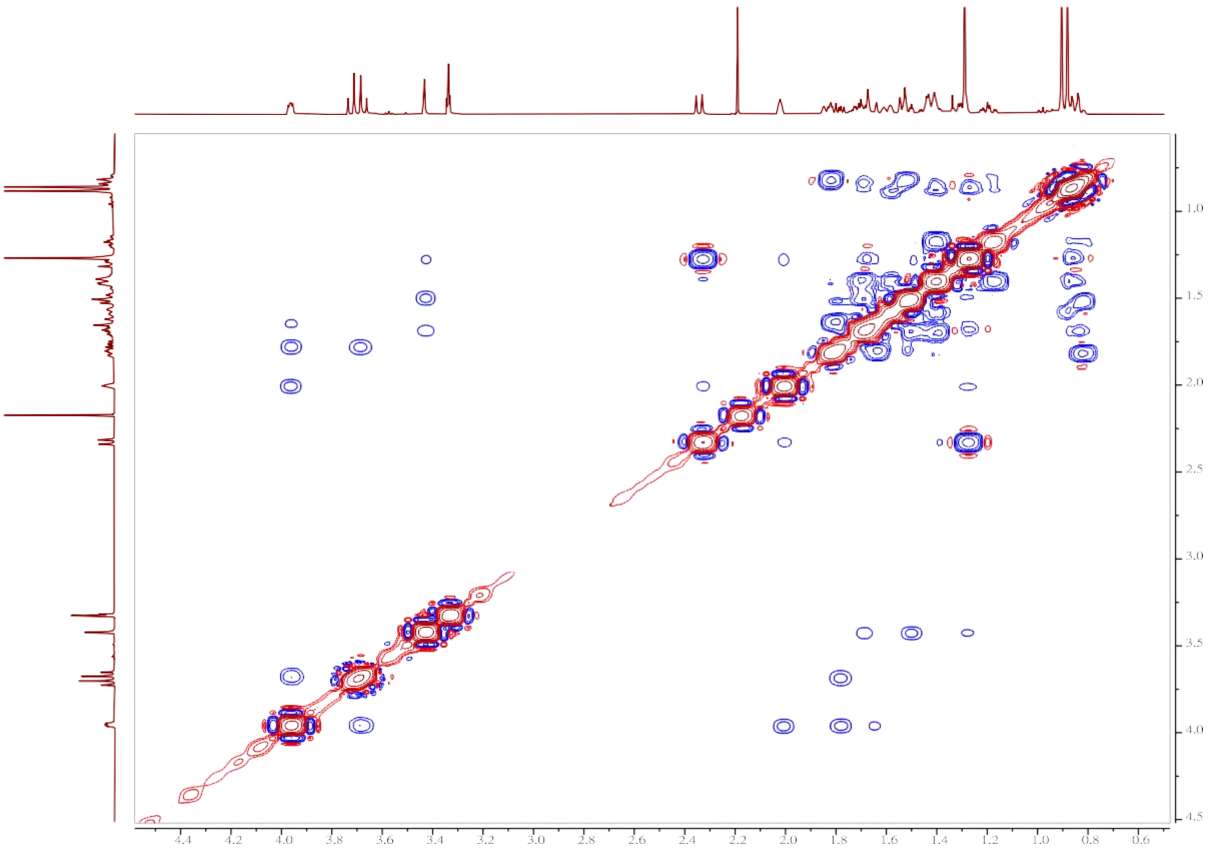

Figure S34. NOESY spectrum of mesonol E (**5**)

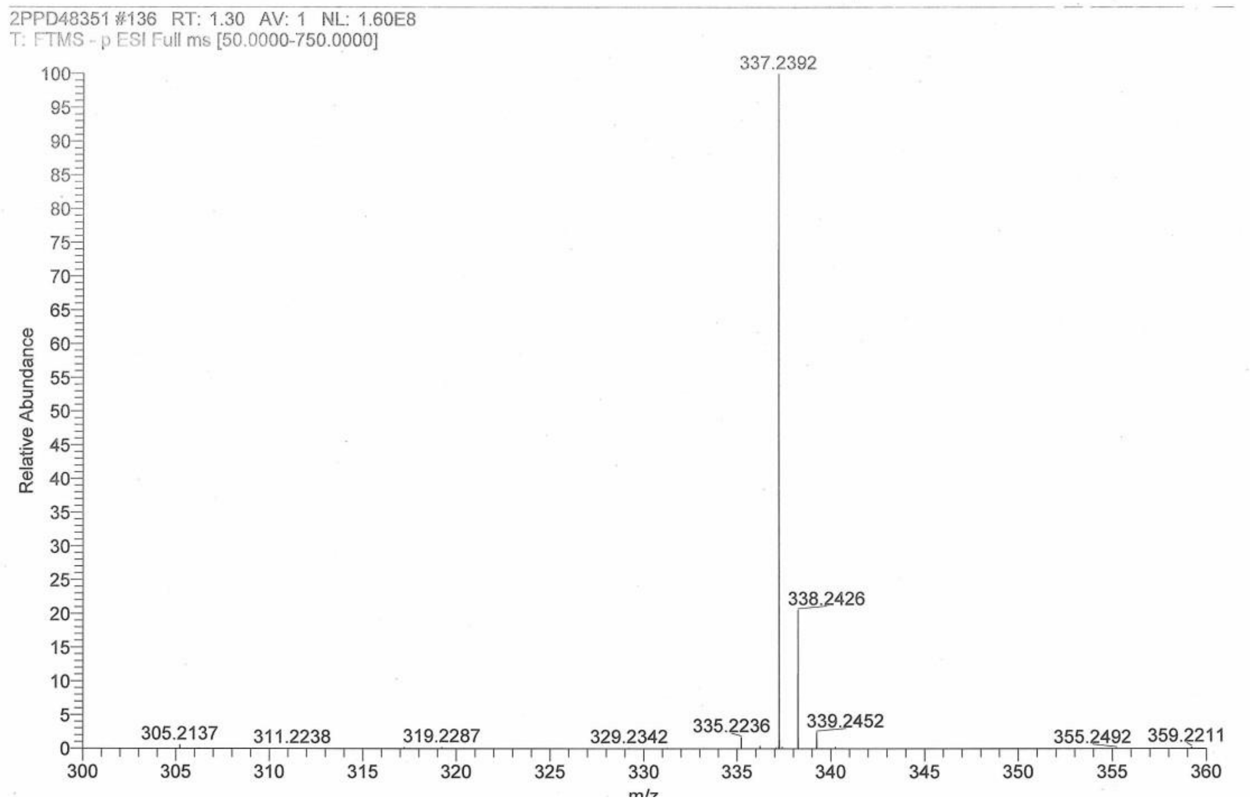

Figure S35. (−)-HRESIMS spectrum of mesonol E (**5**)

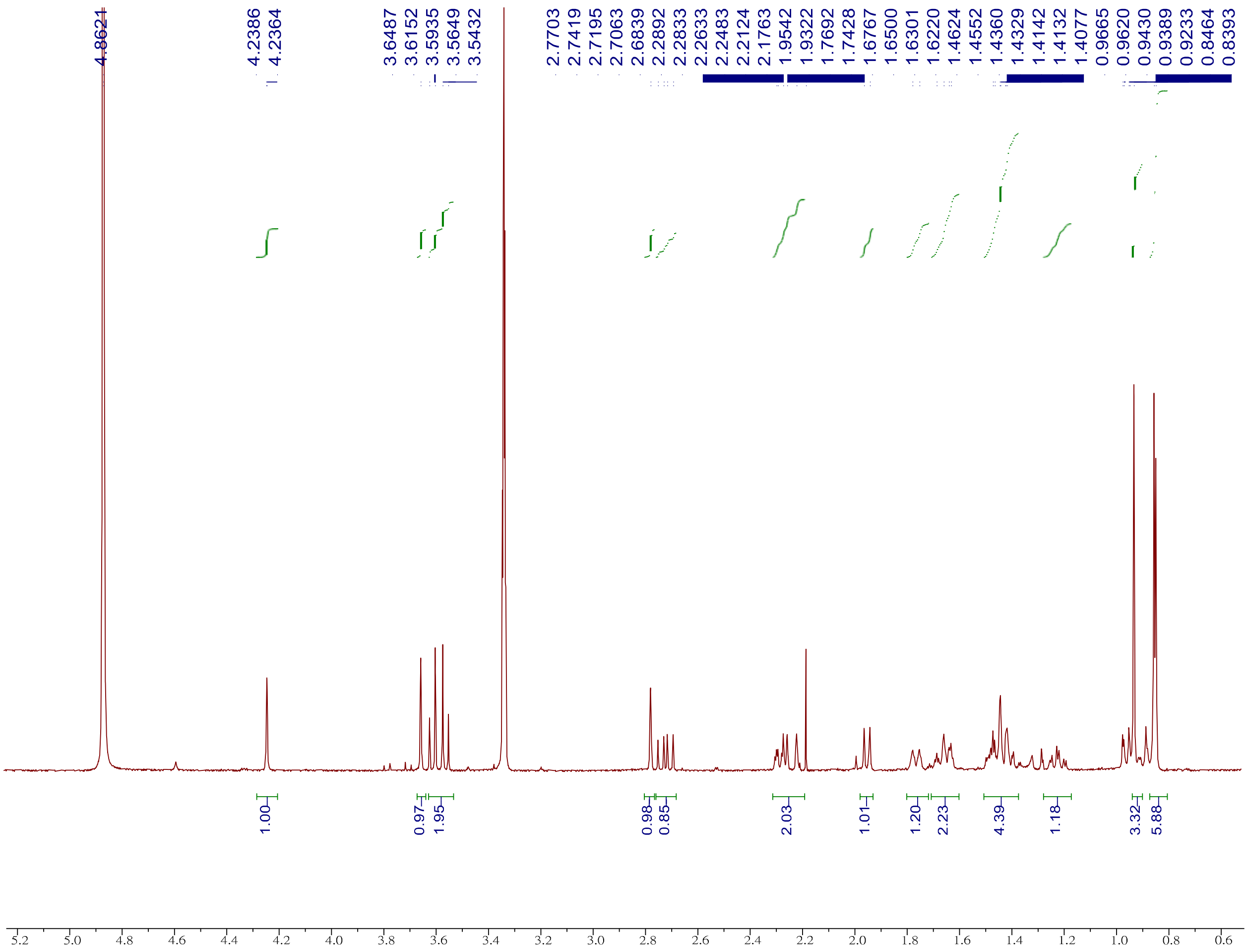

Figure S36. ^{1}H-NMR spectrum of mesonol F (**6**) in MeOD-*d*4 (500 MHz)

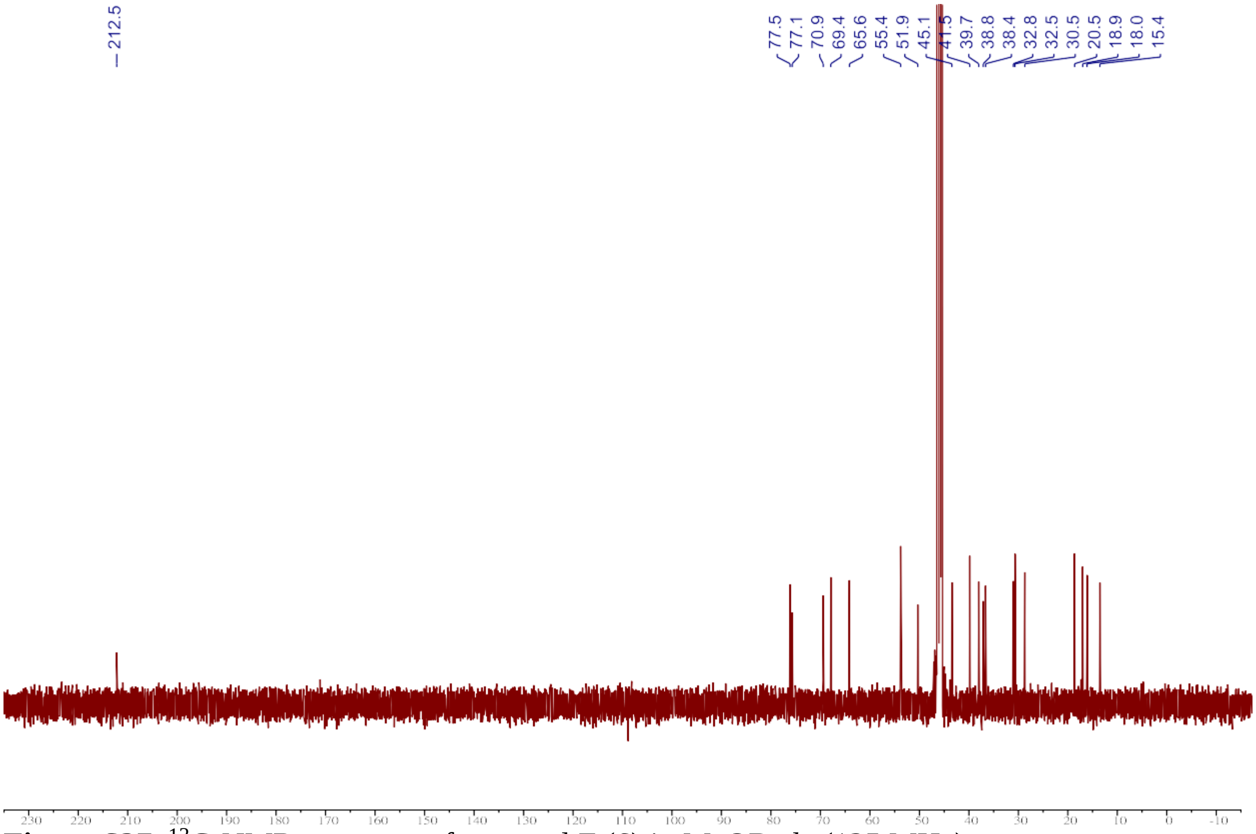

Figure S37. ^{13}C-NMR spectrum of mesonol F (**6**) in MeOD-*d*4 (125 MHz)

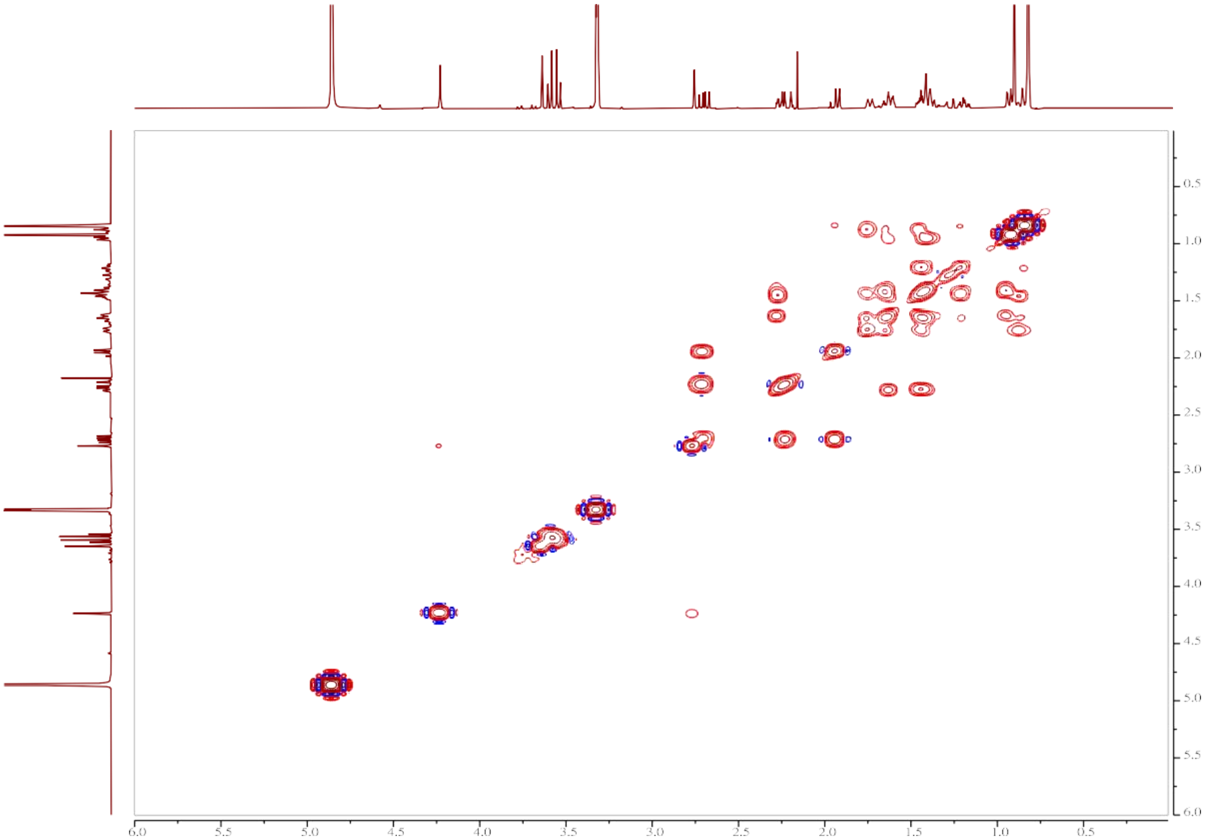

Figure S38. COSY spectrum of mesonol F (**6**)

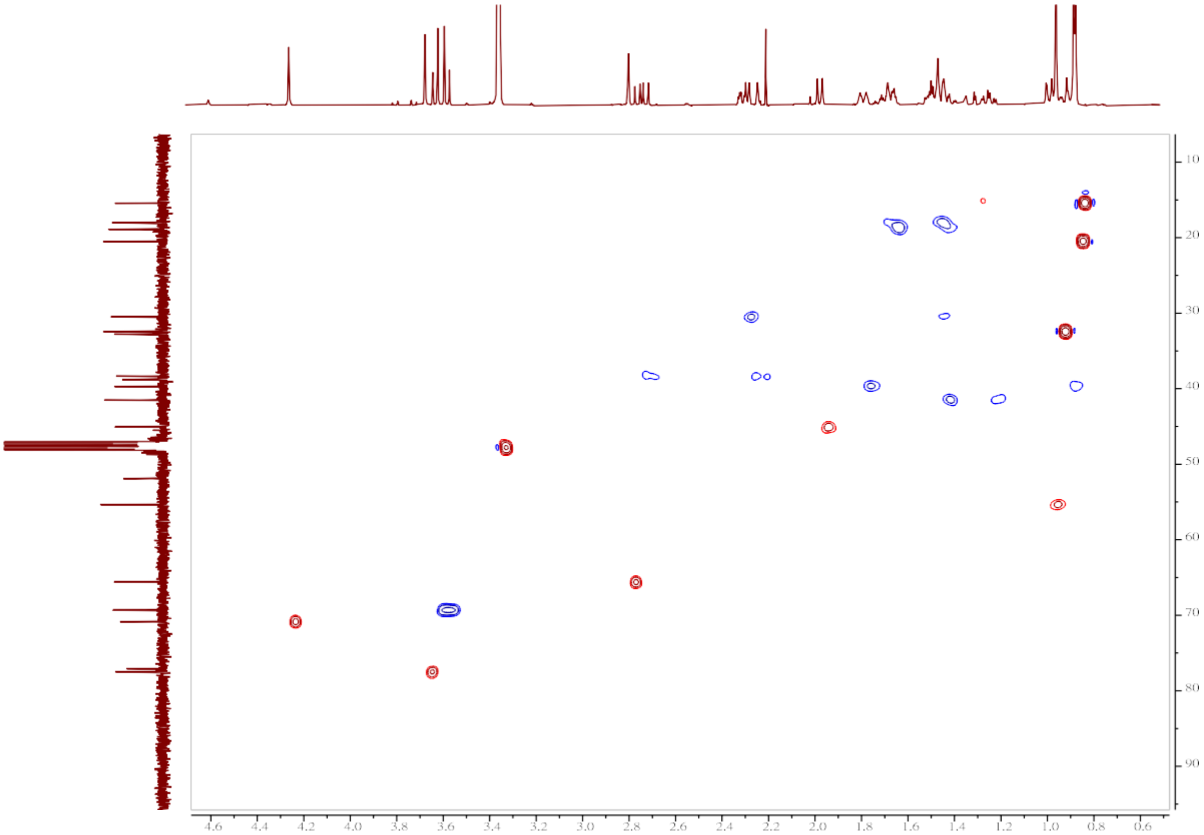

Figure S39. HSQC spectrum of mesonol F (**6**)

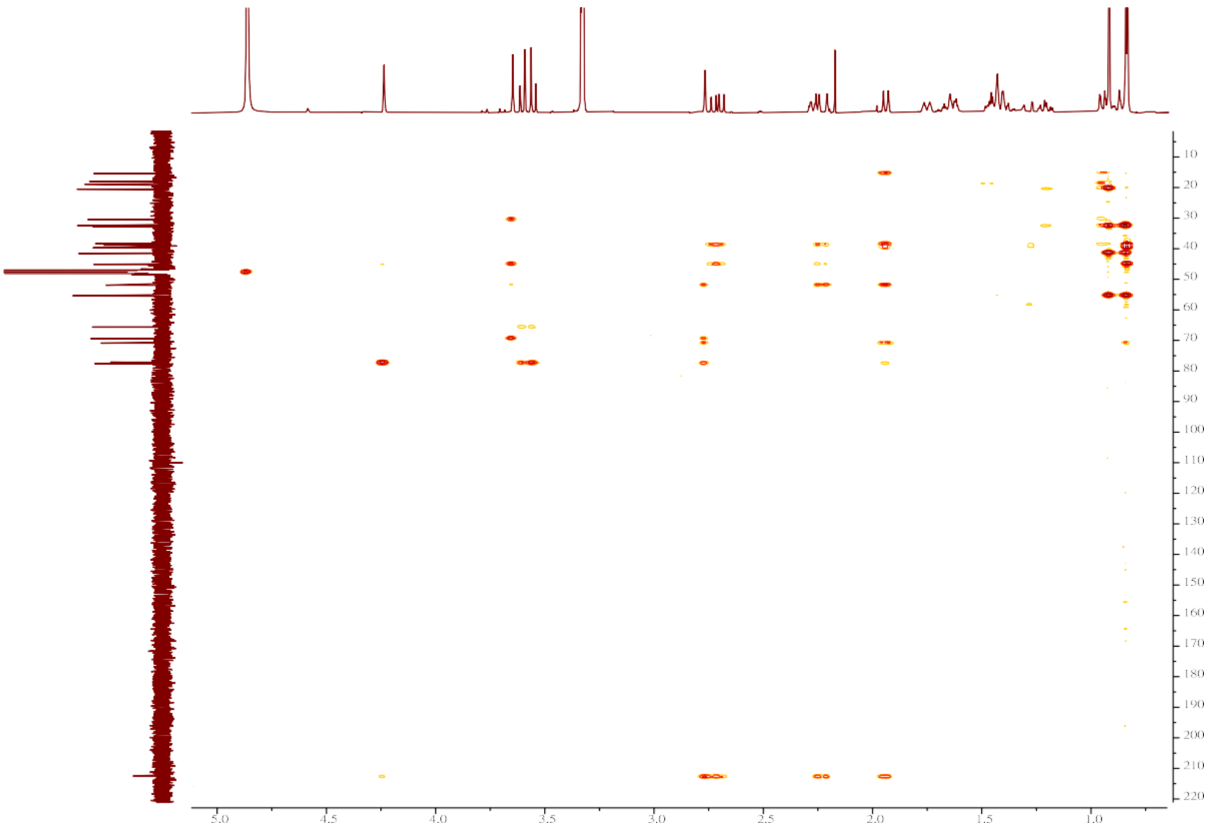

Figure S40. HMBC spectrum of mesonol F (**6**)

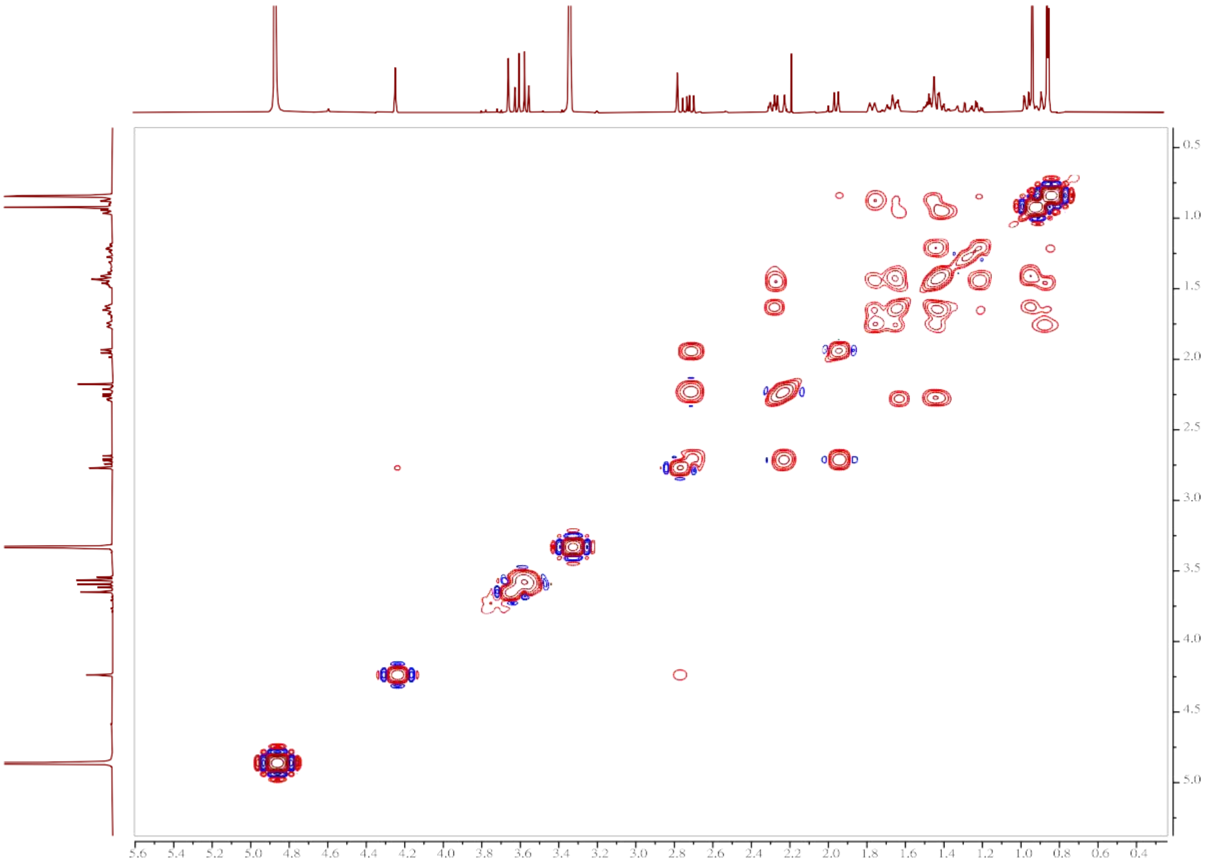

Figure S41. NOESY spectrum of mesonol F (**6**)

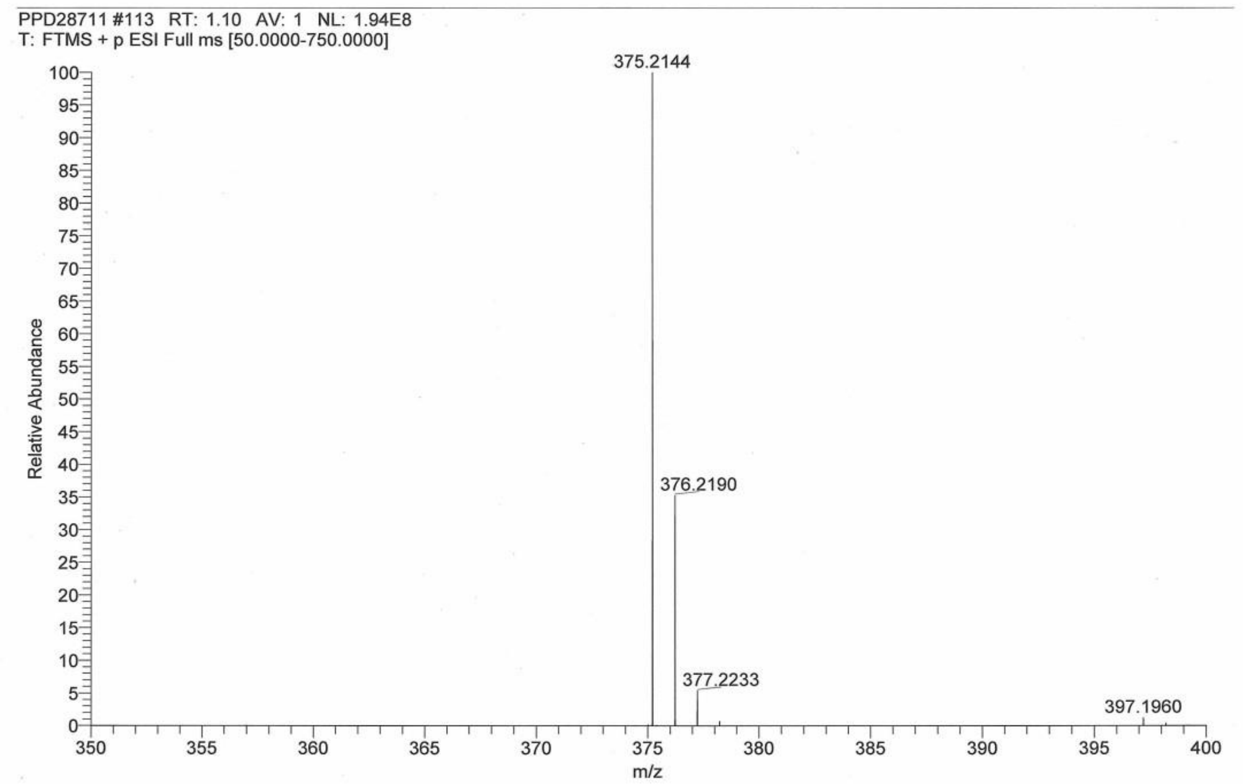

Figure S42. (+)-HRESIMS spectrum of mesonol F (**6**)

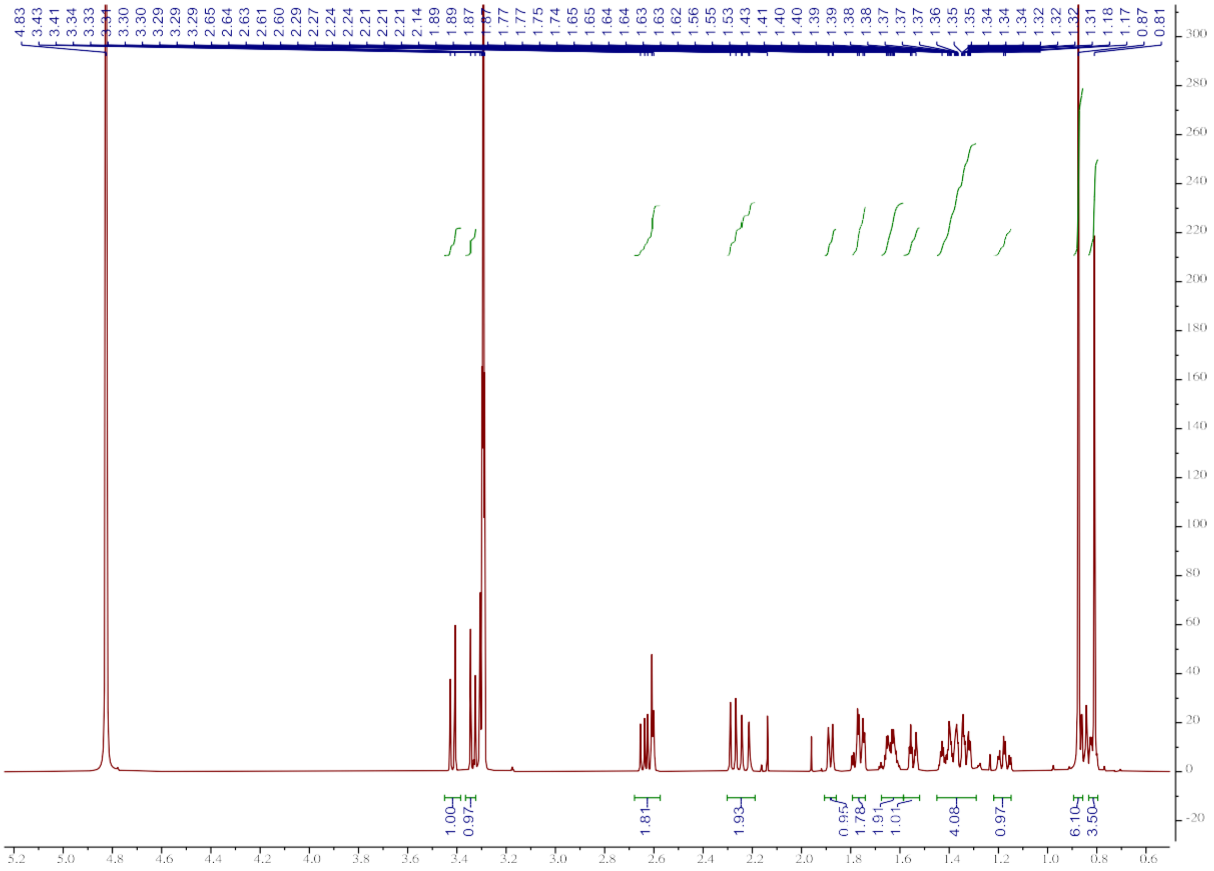

Figure S43. ^{1}H-NMR spectrum of mesonol G (**7**) in MeOD-*d*4 (600 MHz)

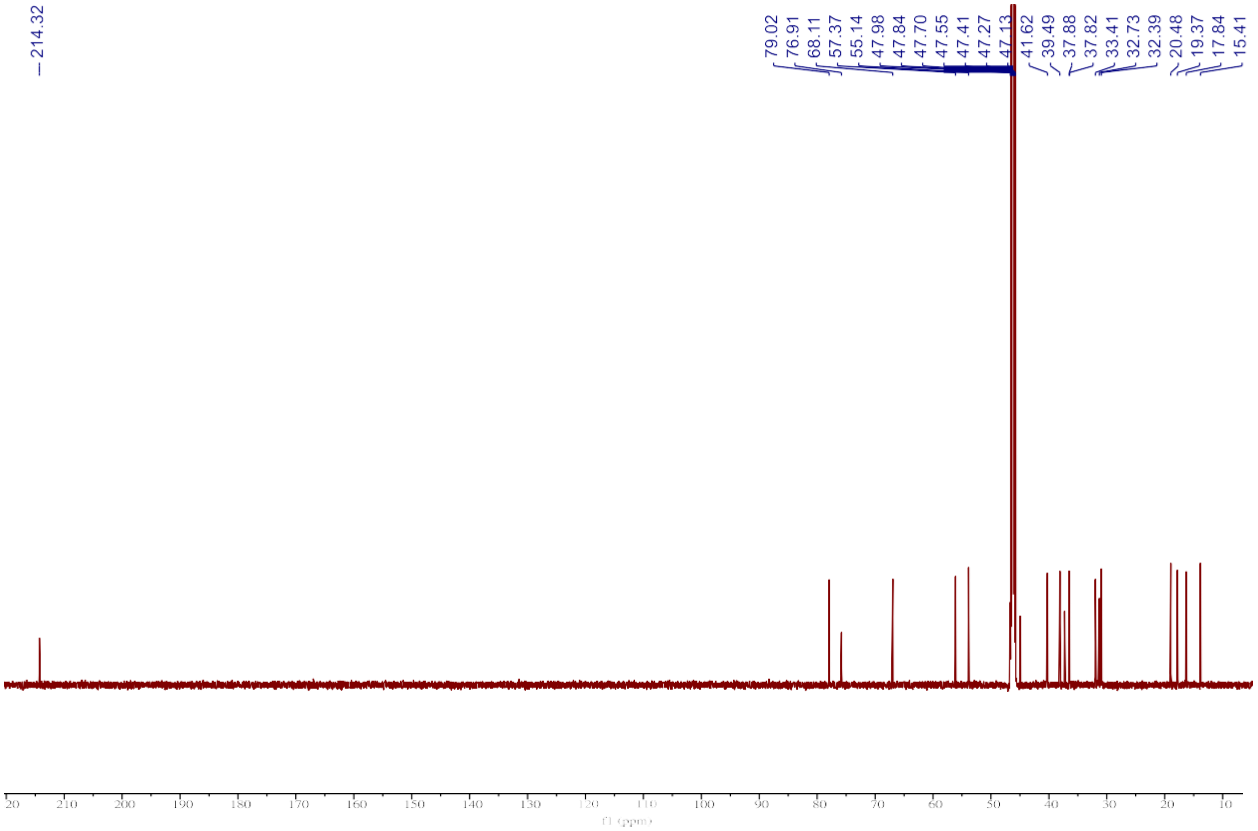

Figure S44. ^{13}C-NMR spectrum of mesonol G (**7**) in MeOD-*d*4 (150MHz)

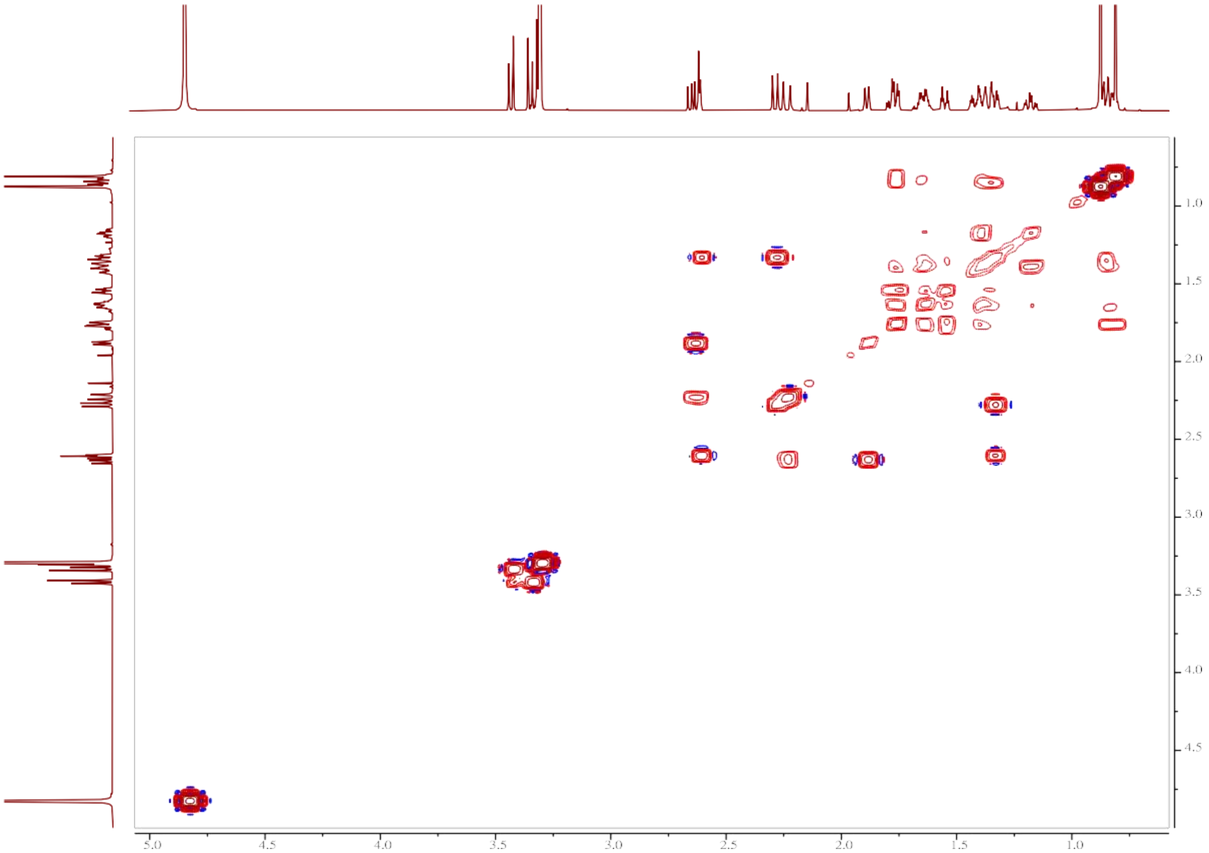

Figure S45. COSY spectrum of mesonol G (**7**)

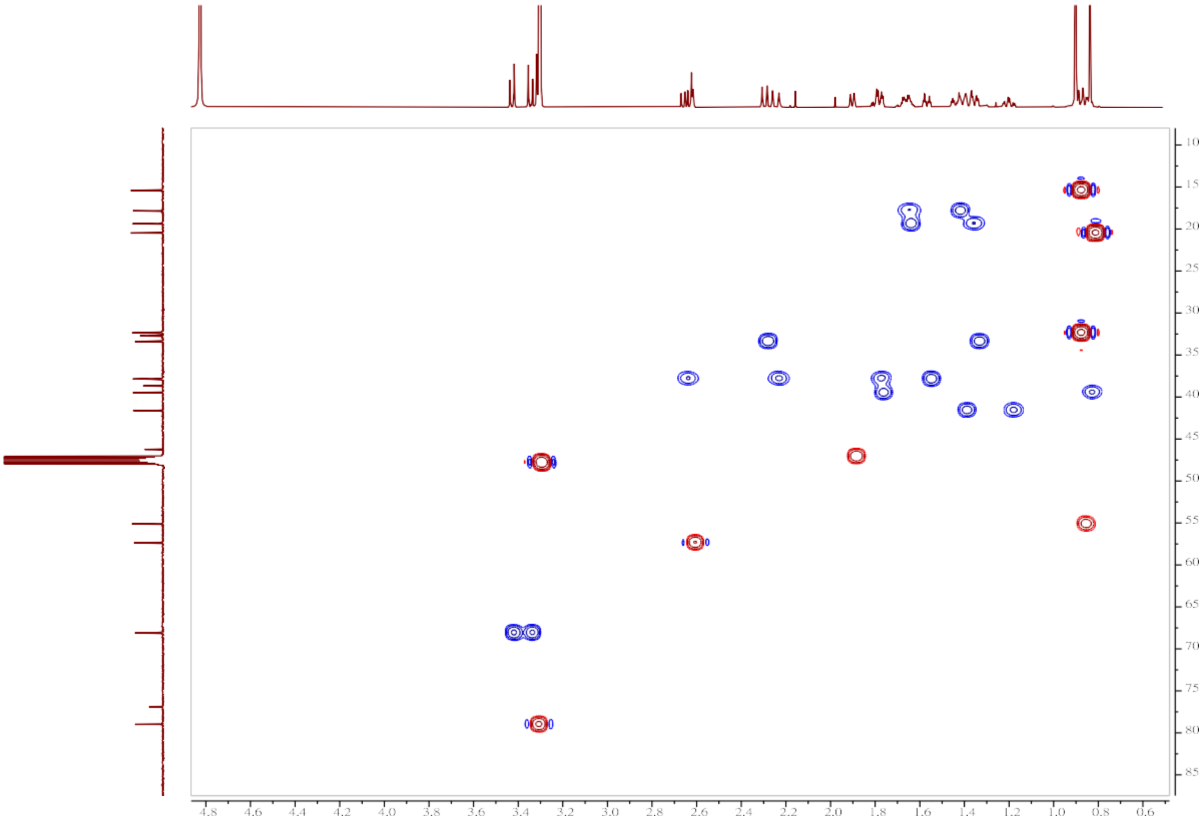

Figure S46. HSQC spectrum of mesonol G (**7**)

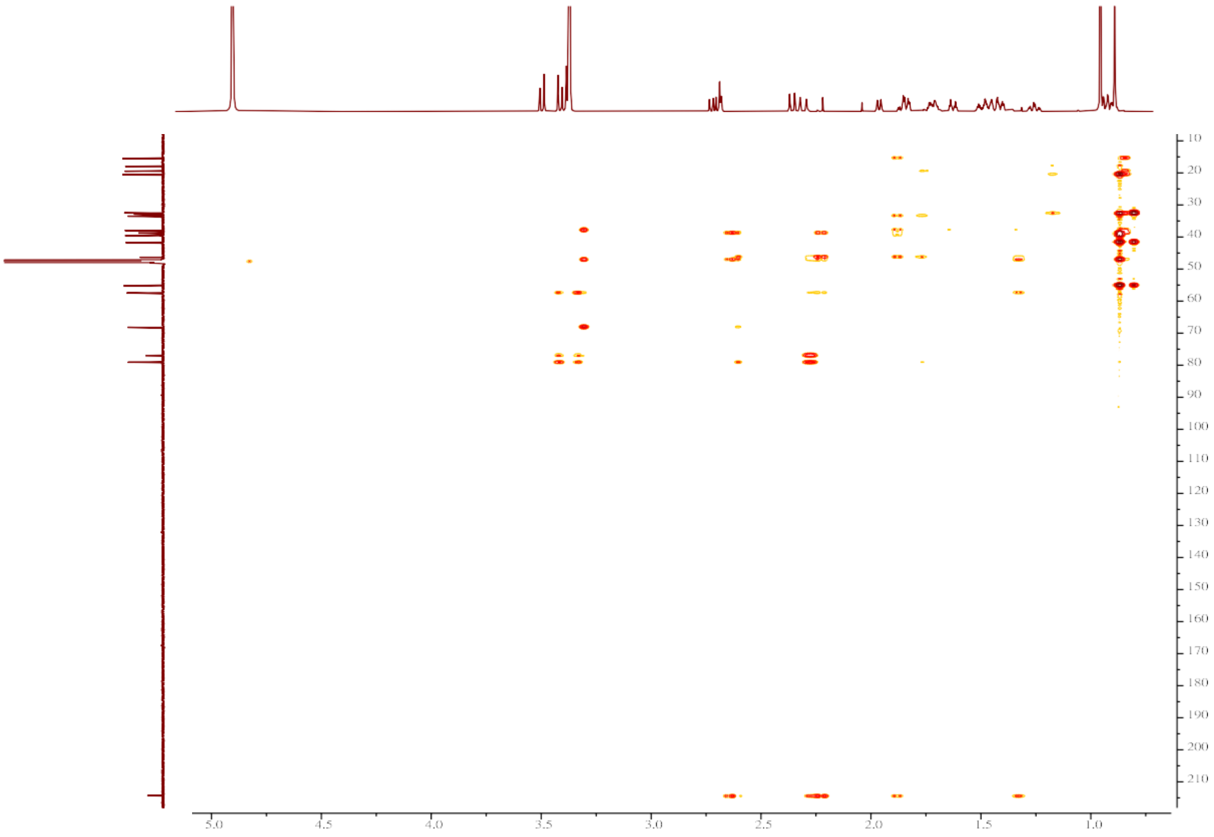

Figure S47. HMBC spectrum of mesonol G (**7**)

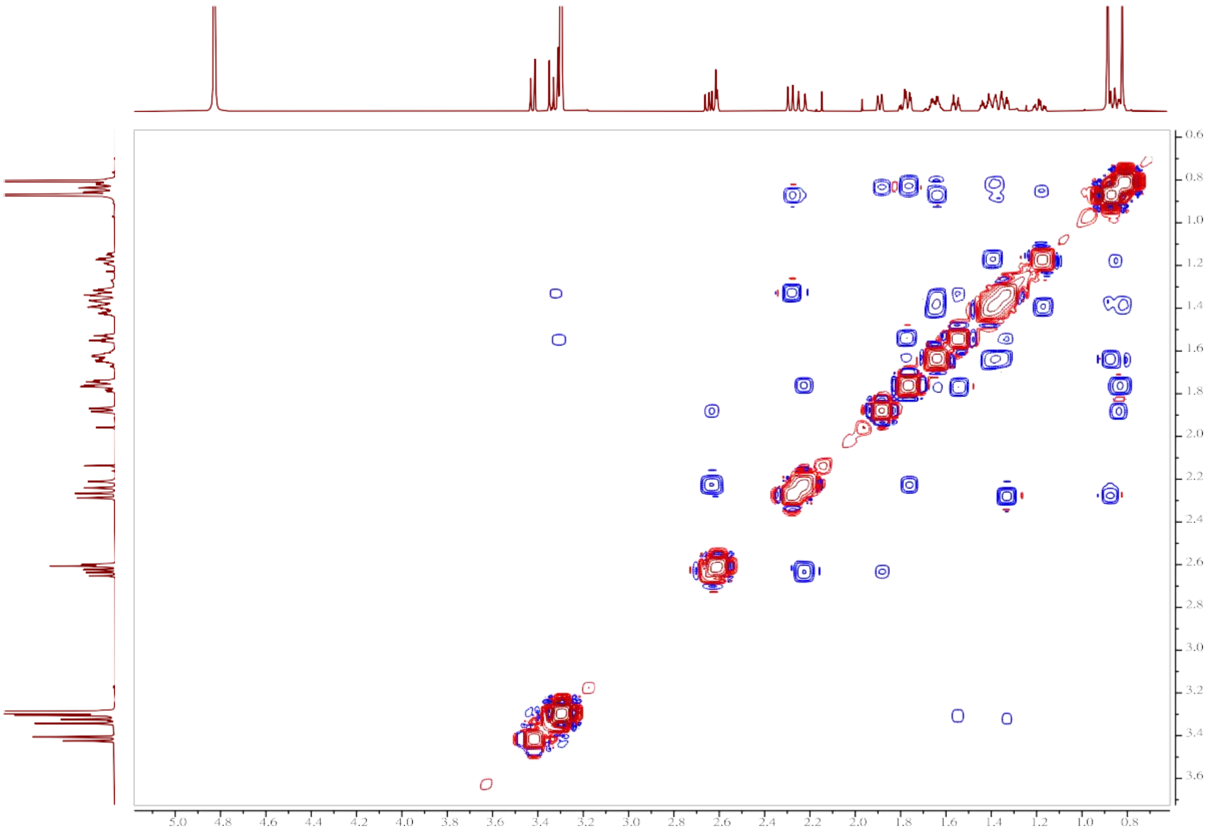

Figure S48. NOESY spectrum of mesonol G (**7**)

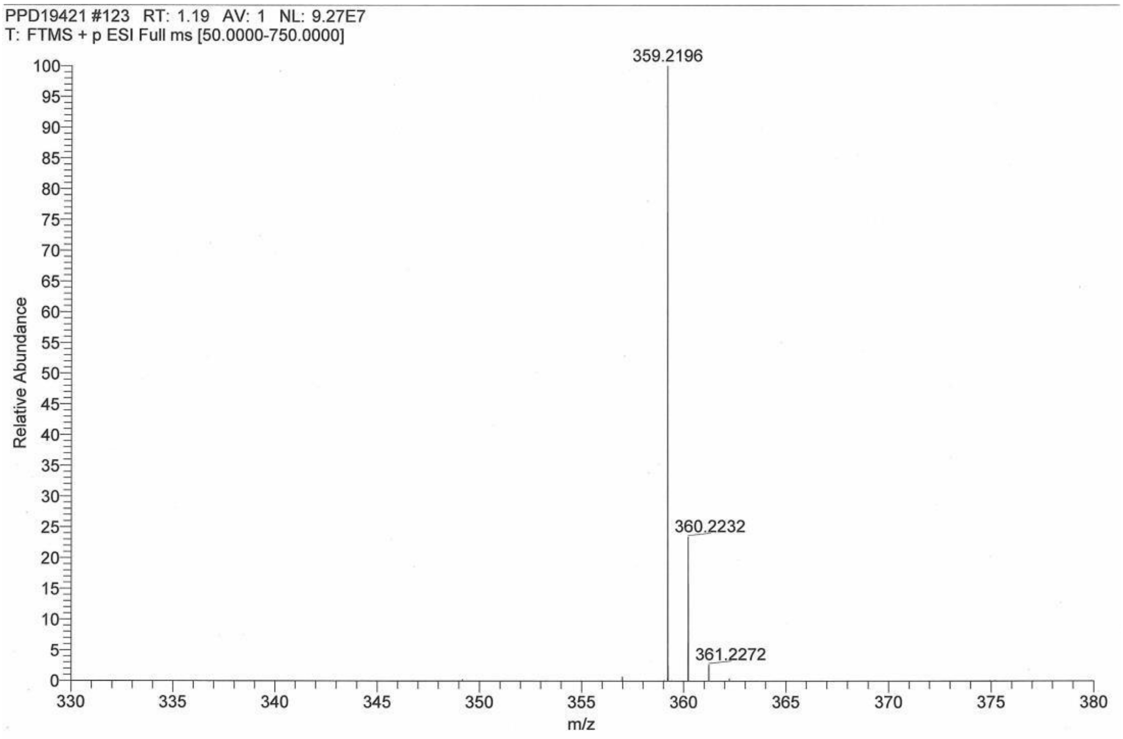

Figure S49. (+)-HRESIMS spectrum of mesonol G (**7**)

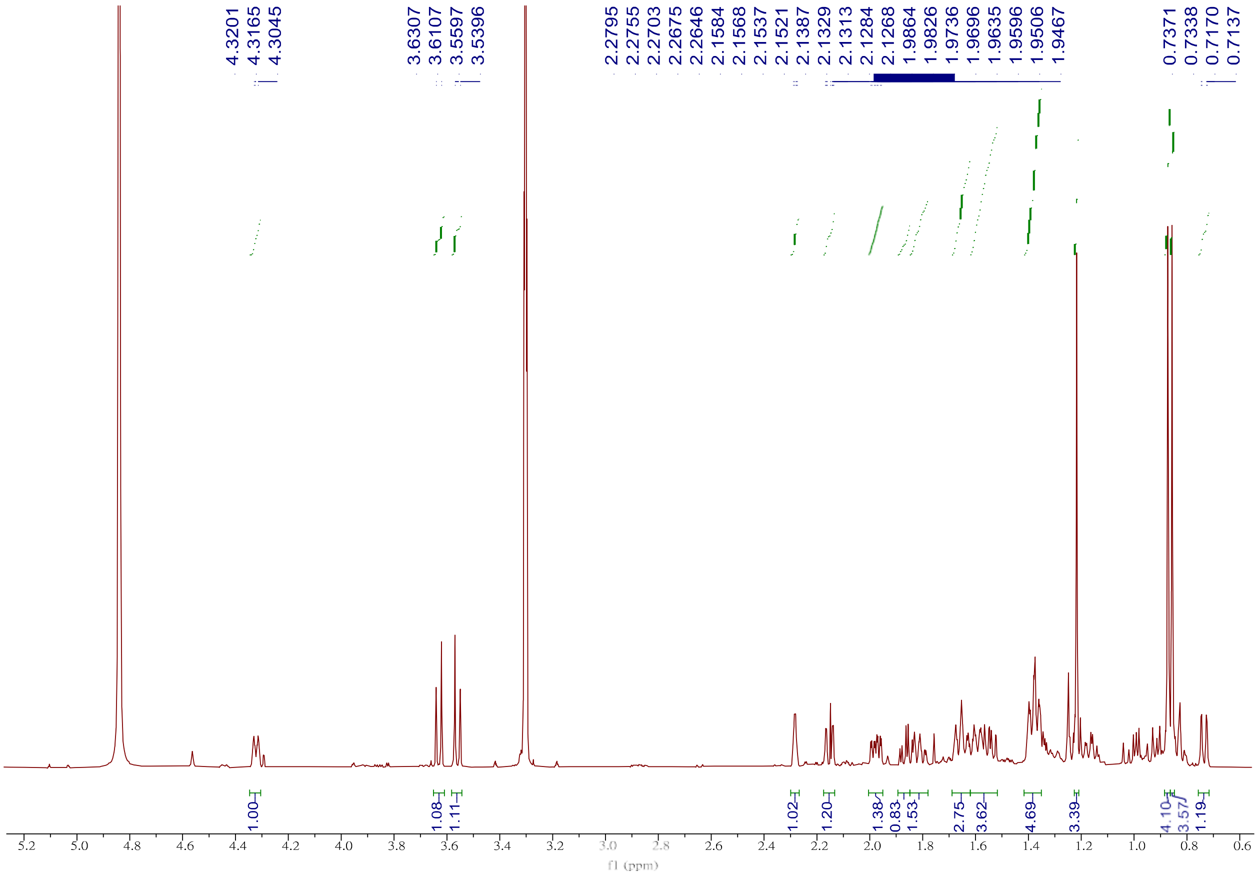

Figure S50. ^{1}H-NMR spectrum of mesonol H (**8**) in MeOD-*d*4 (600 MHz)

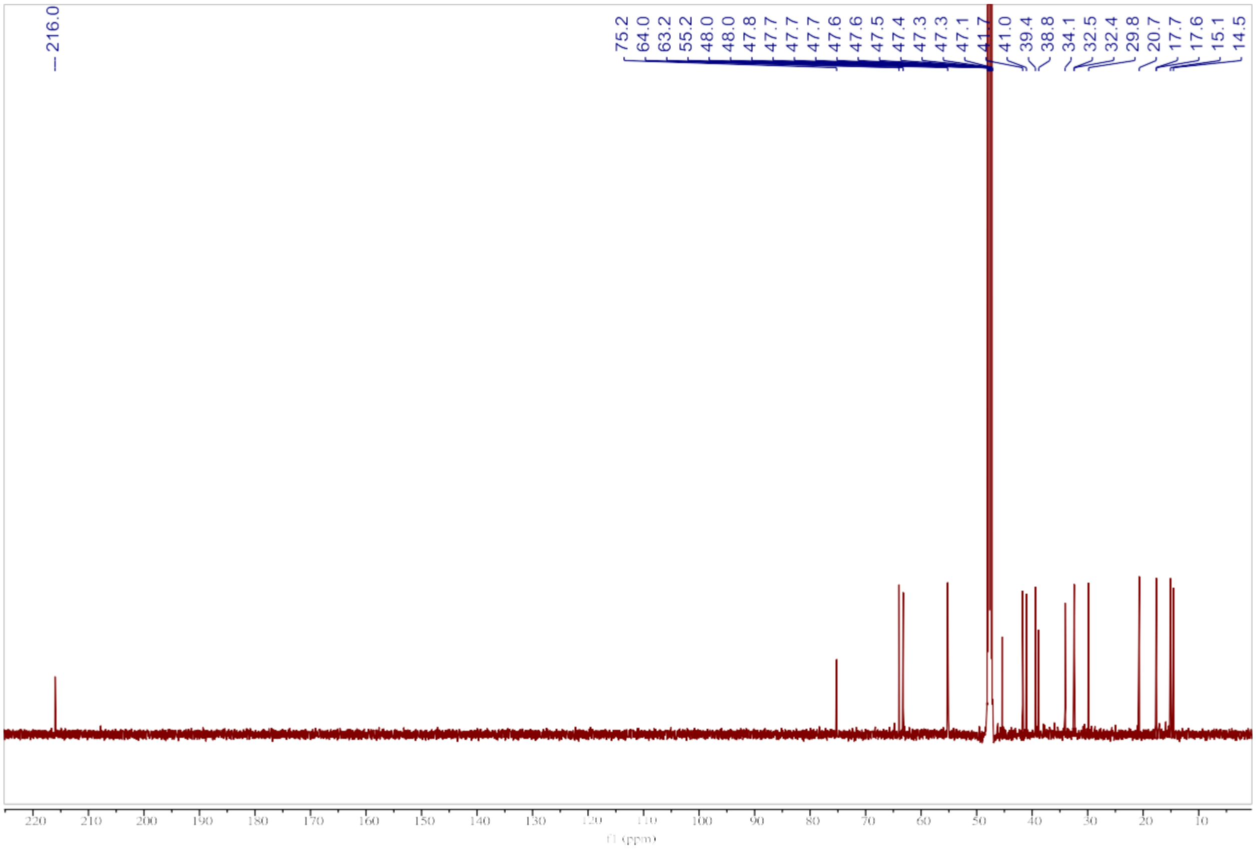

Figure S51. ^{13}C-NMR spectrum of mesonol H (**8**) in MeOD-*d*4 (150 MHz)

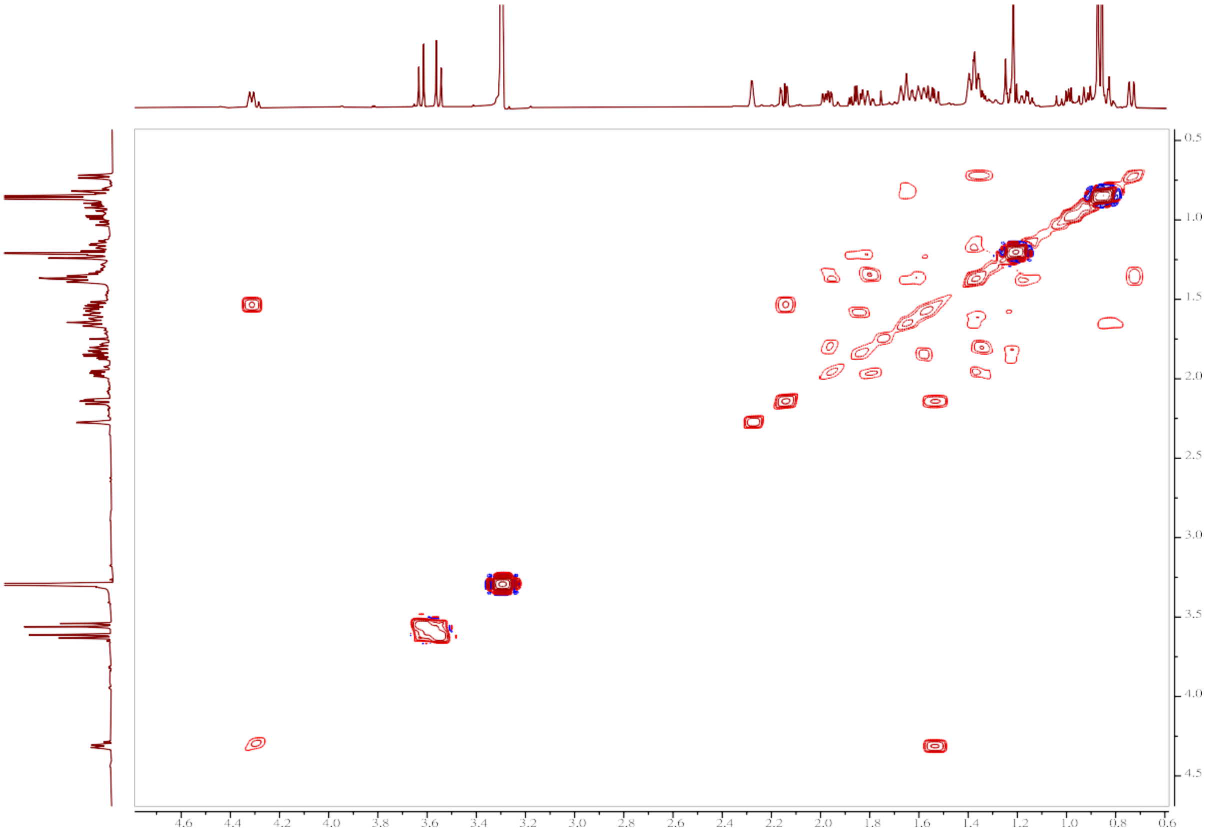

Figure S52. COSY spectrum of mesonol H (**8**)

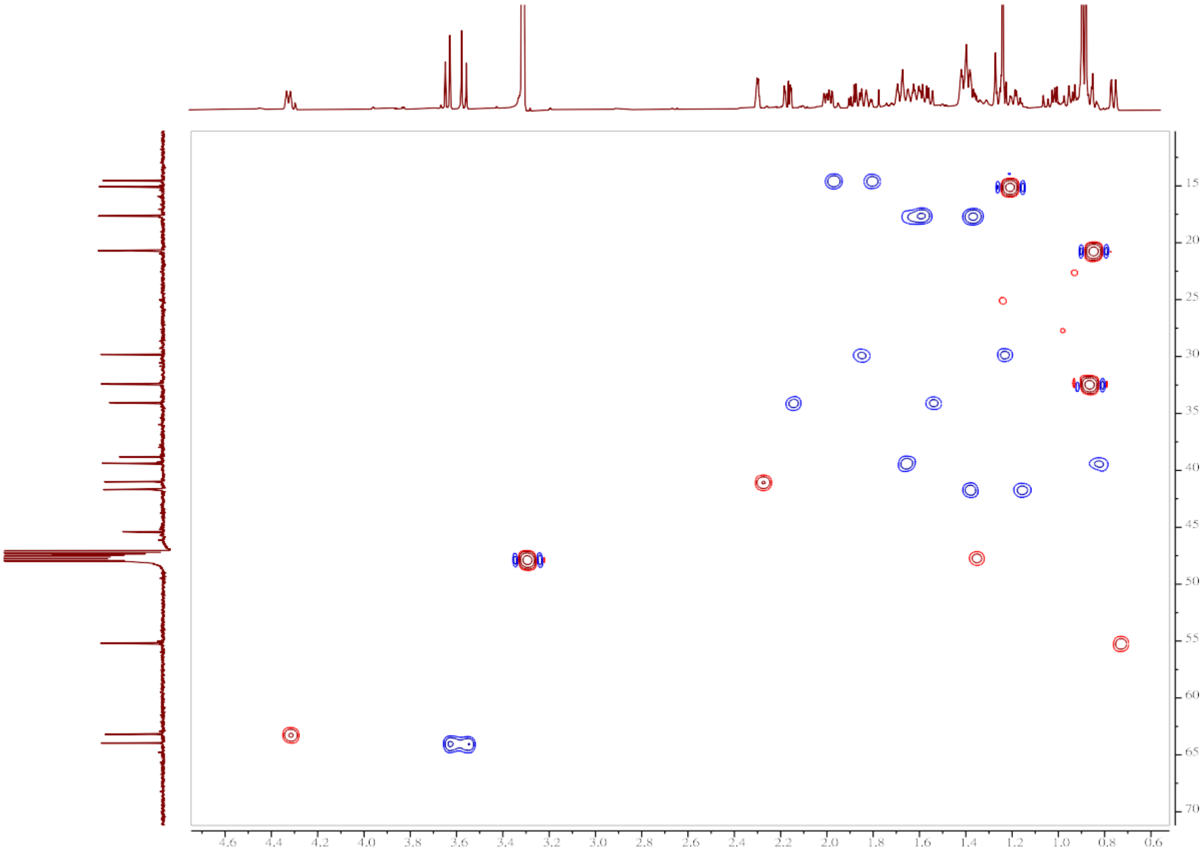

Figure S53. HSQC spectrum of mesonol H (**8**)

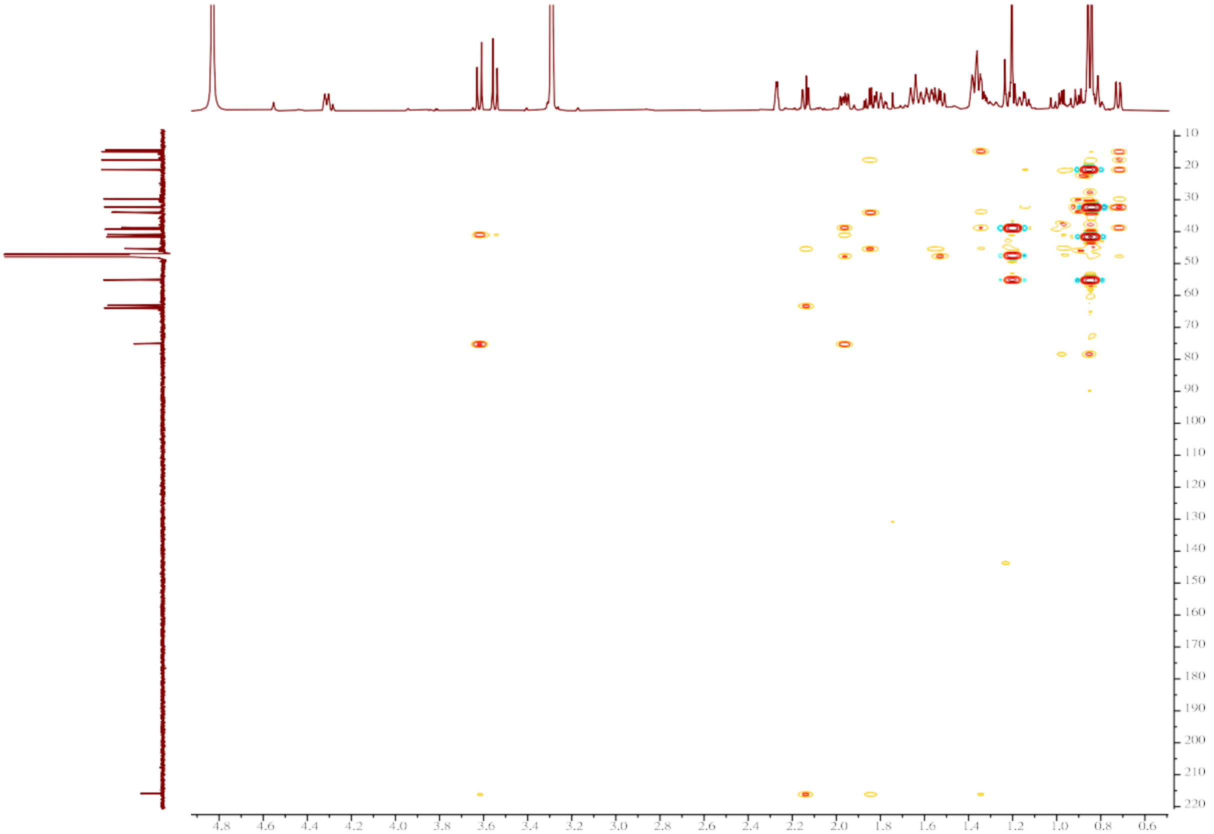

Figure S54. HMBC spectrum of mesonol H (**8**)

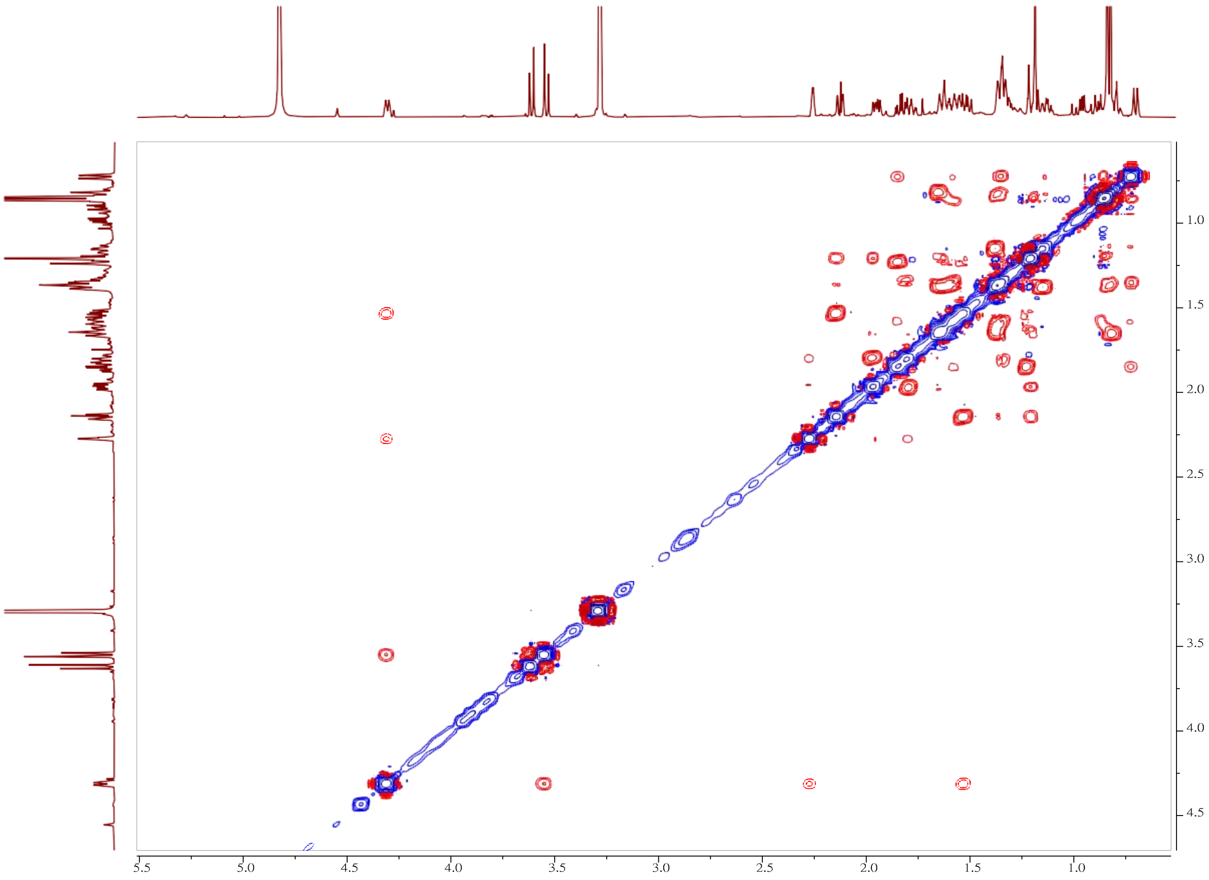

Figure S55. NOESY spectrum of mesonol H (**8**)

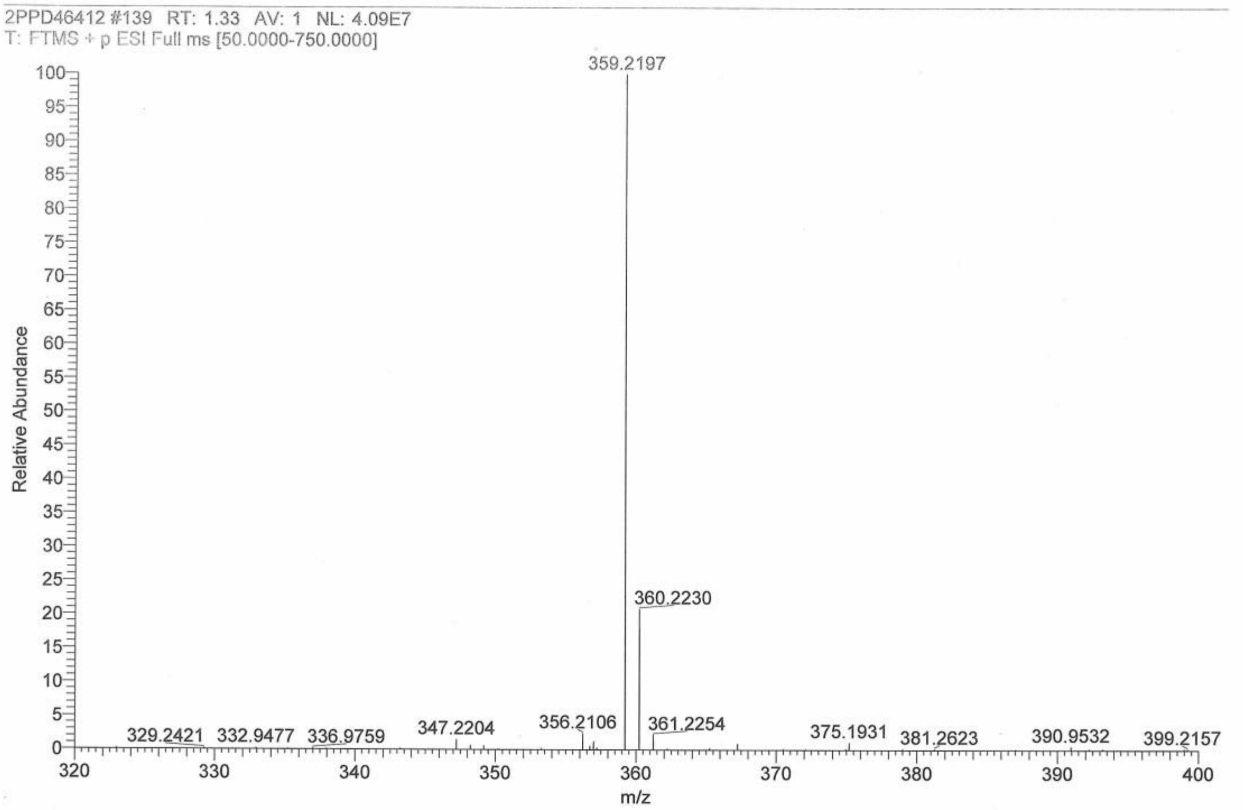

Figure S56. (+)-HRESIMS spectrum of mesonol H (**8**)

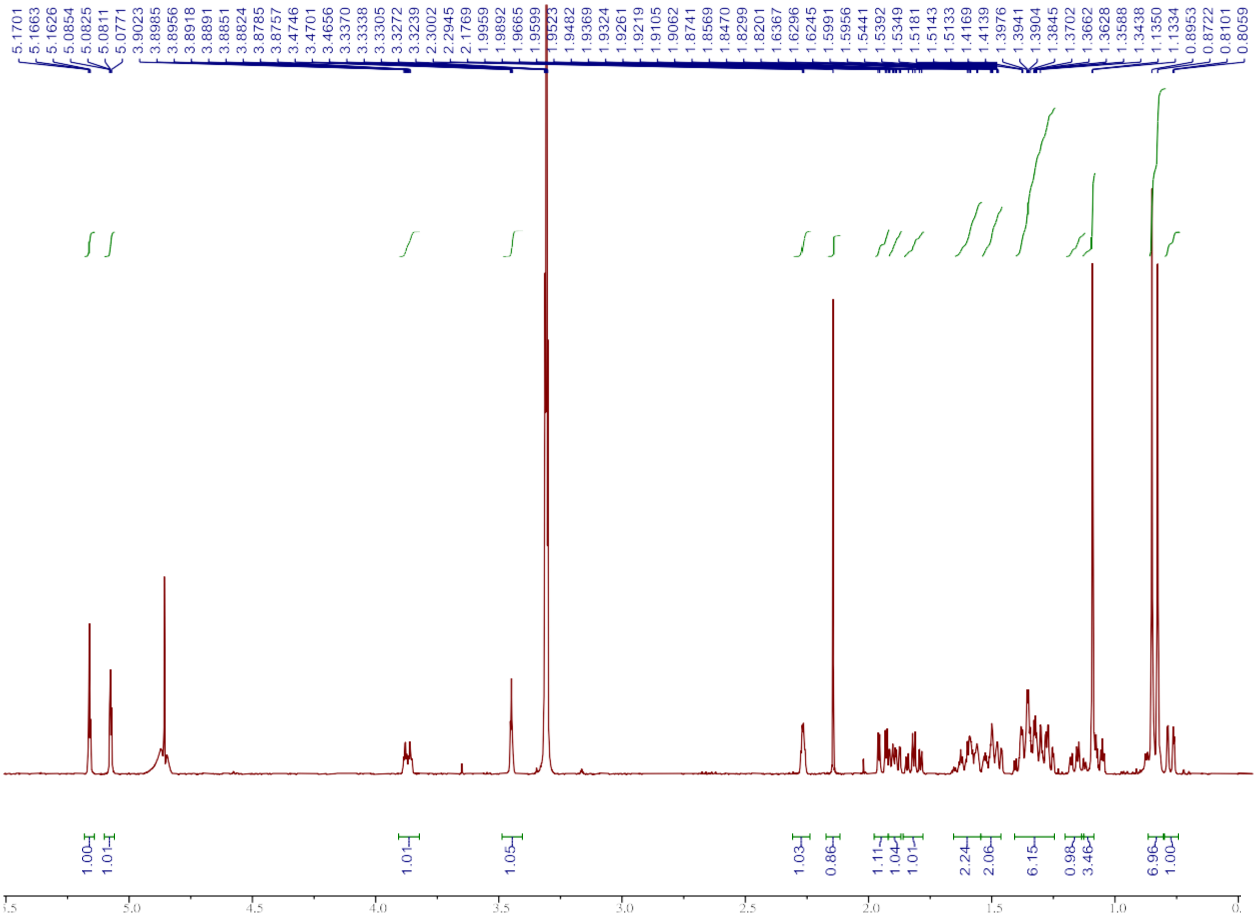

Figure S57. ^{1}H-NMR spectrum of mesonol I (**9**) in MeOD-*d*4 (500 MHz)

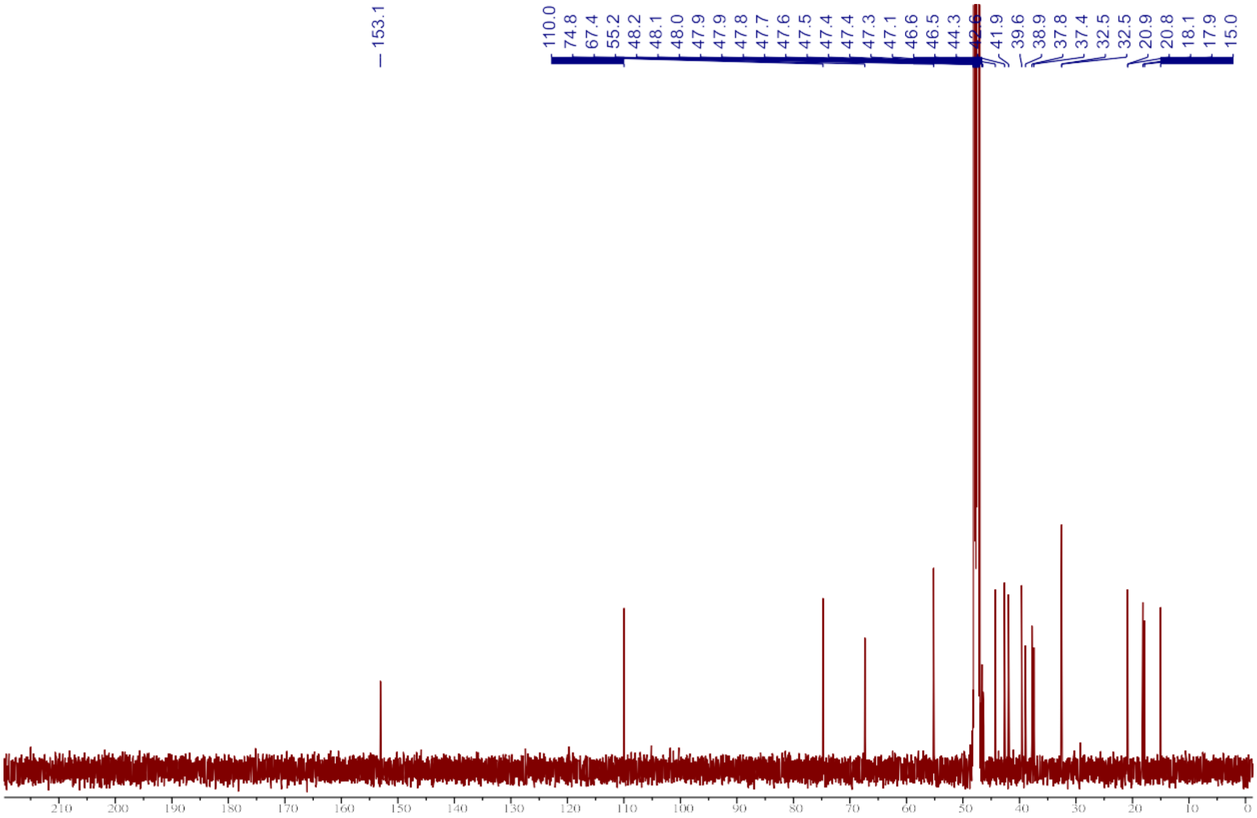

Figure S58. ^{13}C-NMR spectrum of mesonol I (**9**) in MeOD-*d*4 (125 MHz)

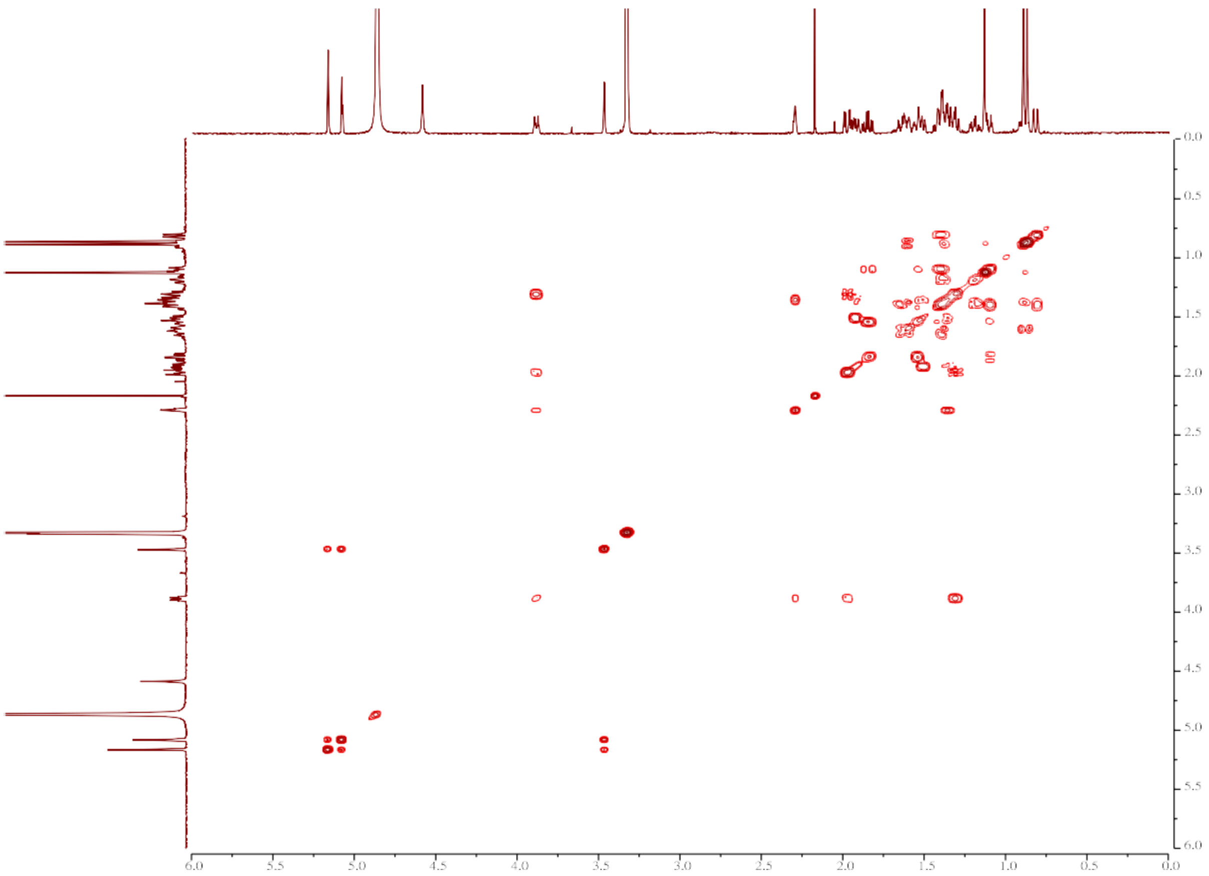

Figure S59. COSY spectrum of mesonol I (**9**)

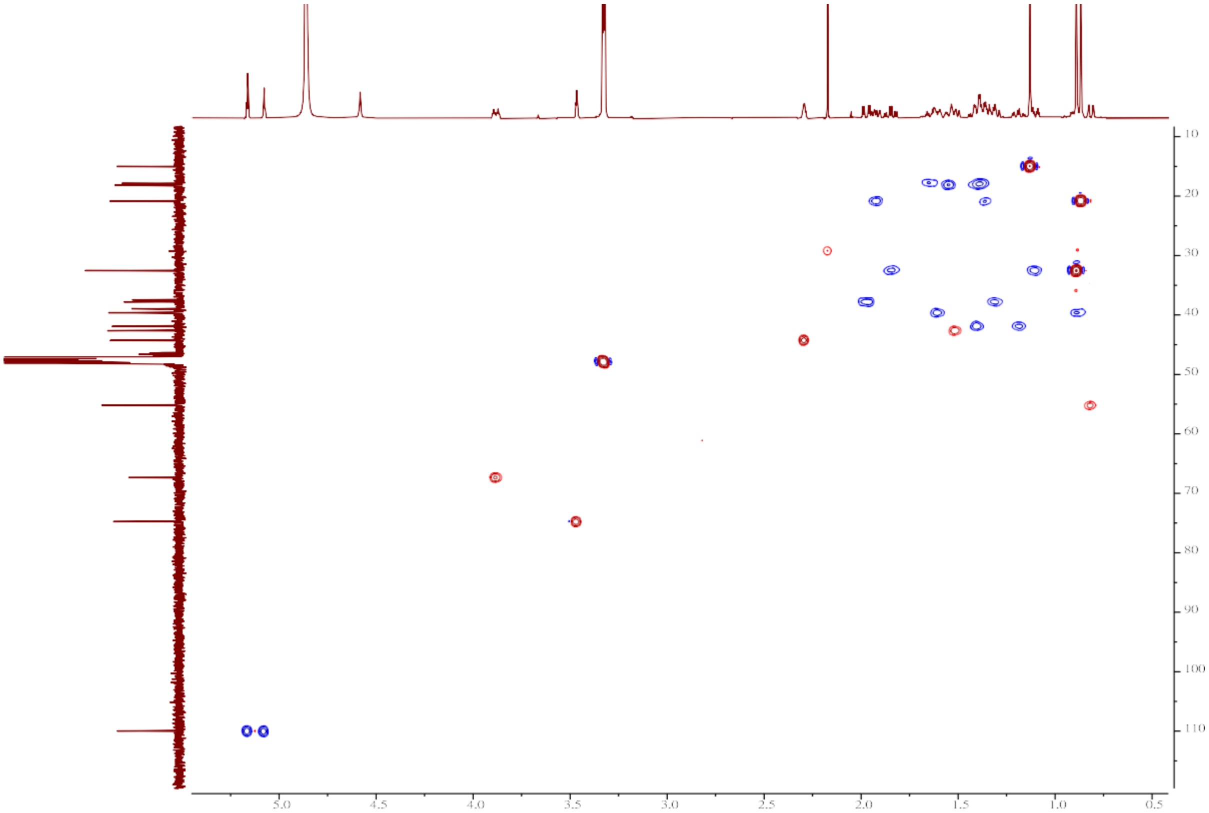

Figure S60. HSQC spectrum of mesonol I (**9**)

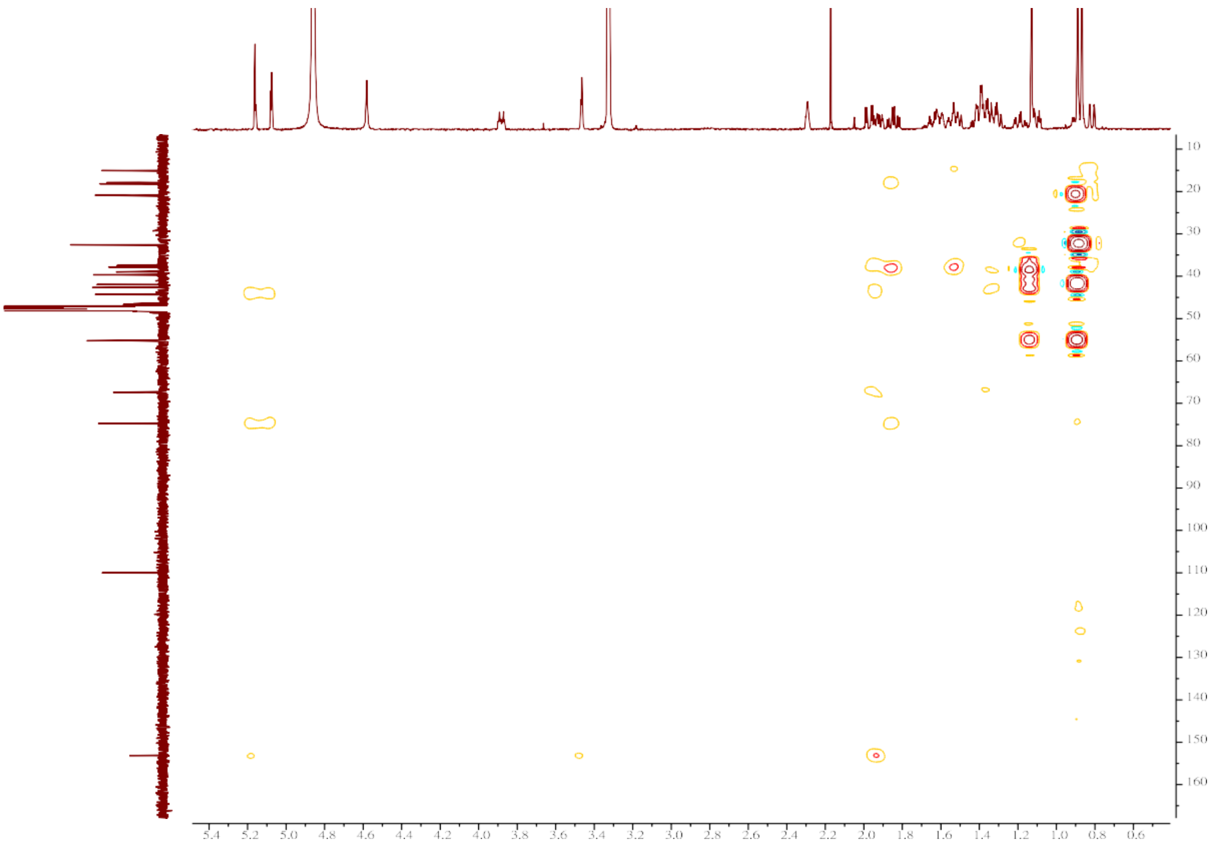

Figure S61. HMBC spectrum of mesonol I (**9**)

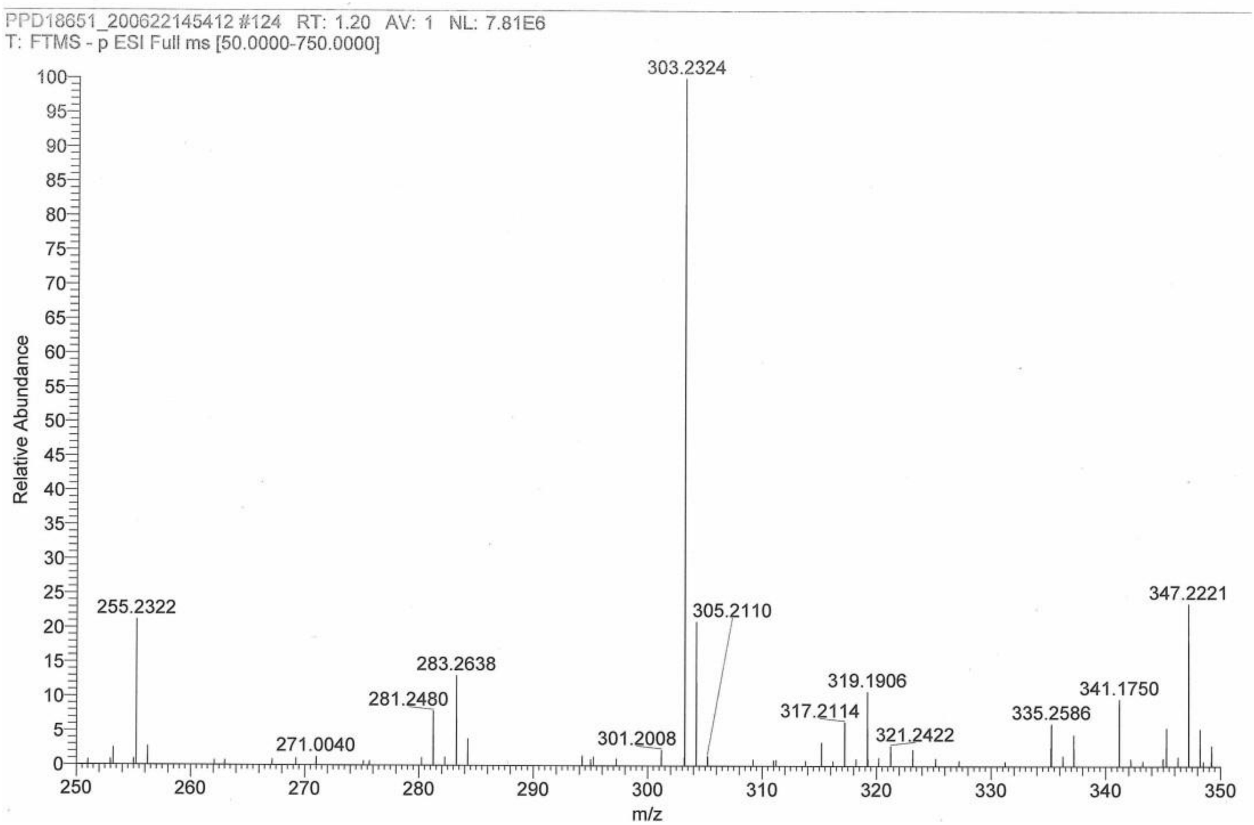

Figure S62. (−)-HRESIMS spectrum of mesonol I (**9**)

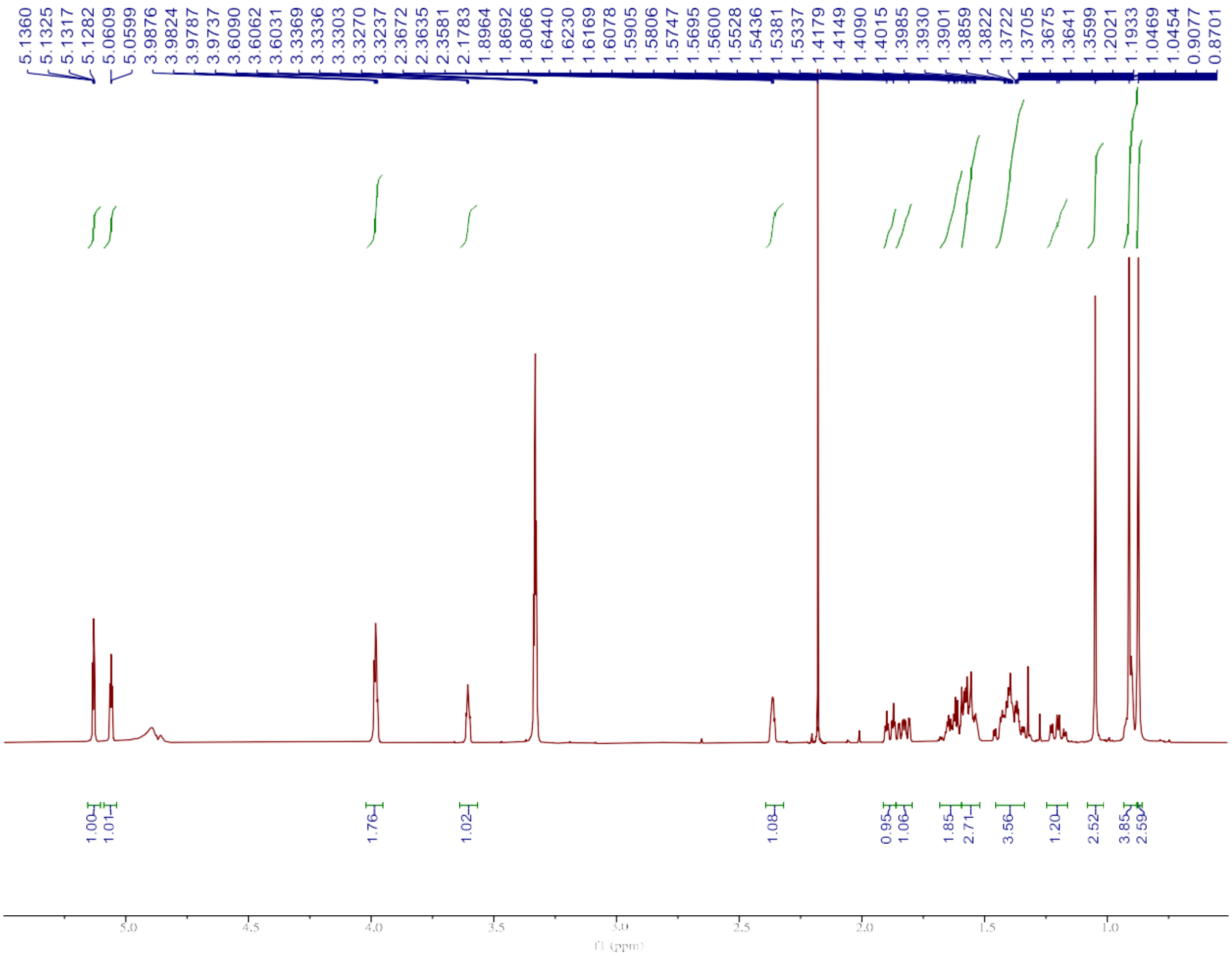

Figure S63. ^{1}H-NMR spectrum of mesonol J (**10**) in MeOD-*d*4 (500 MHz)

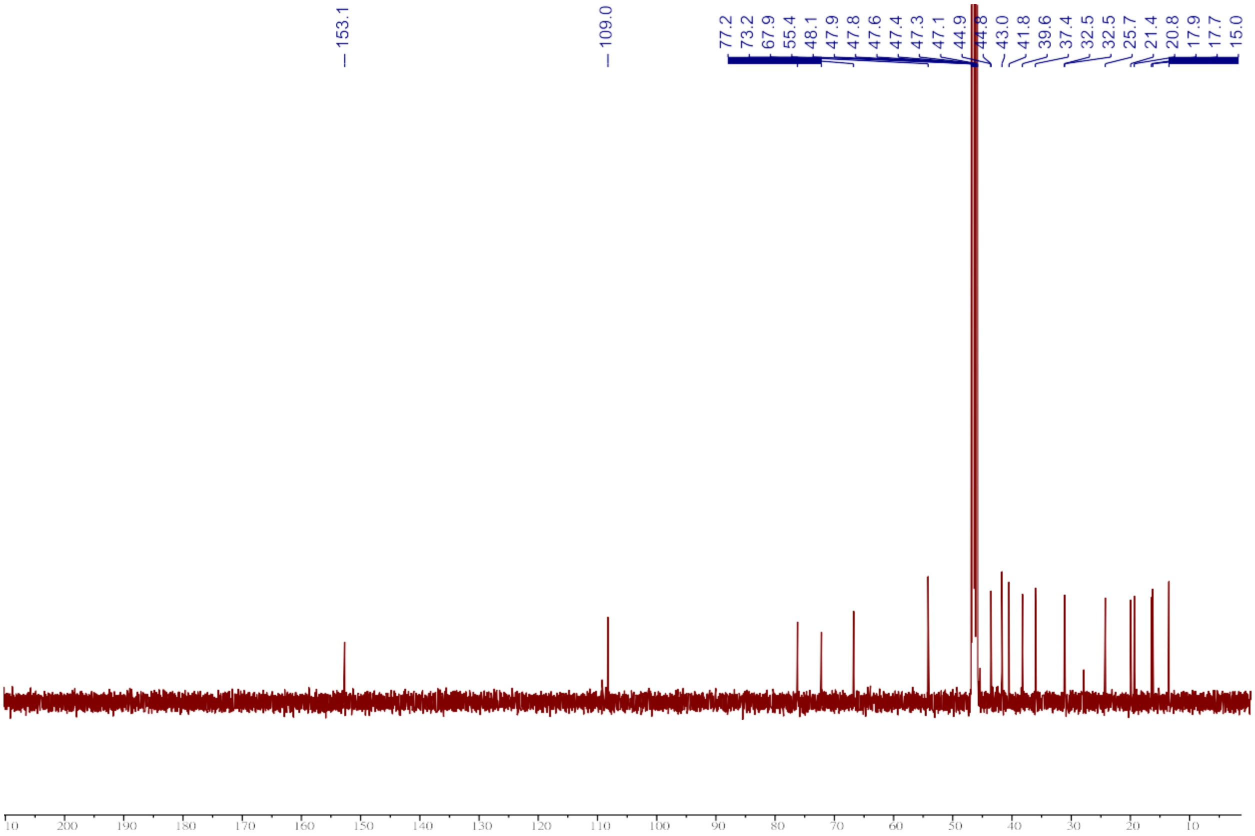

Figure S64. ^{13}C-NMR spectrum of mesonol J (**10**) in MeOD-*d*4 (125 MHz)

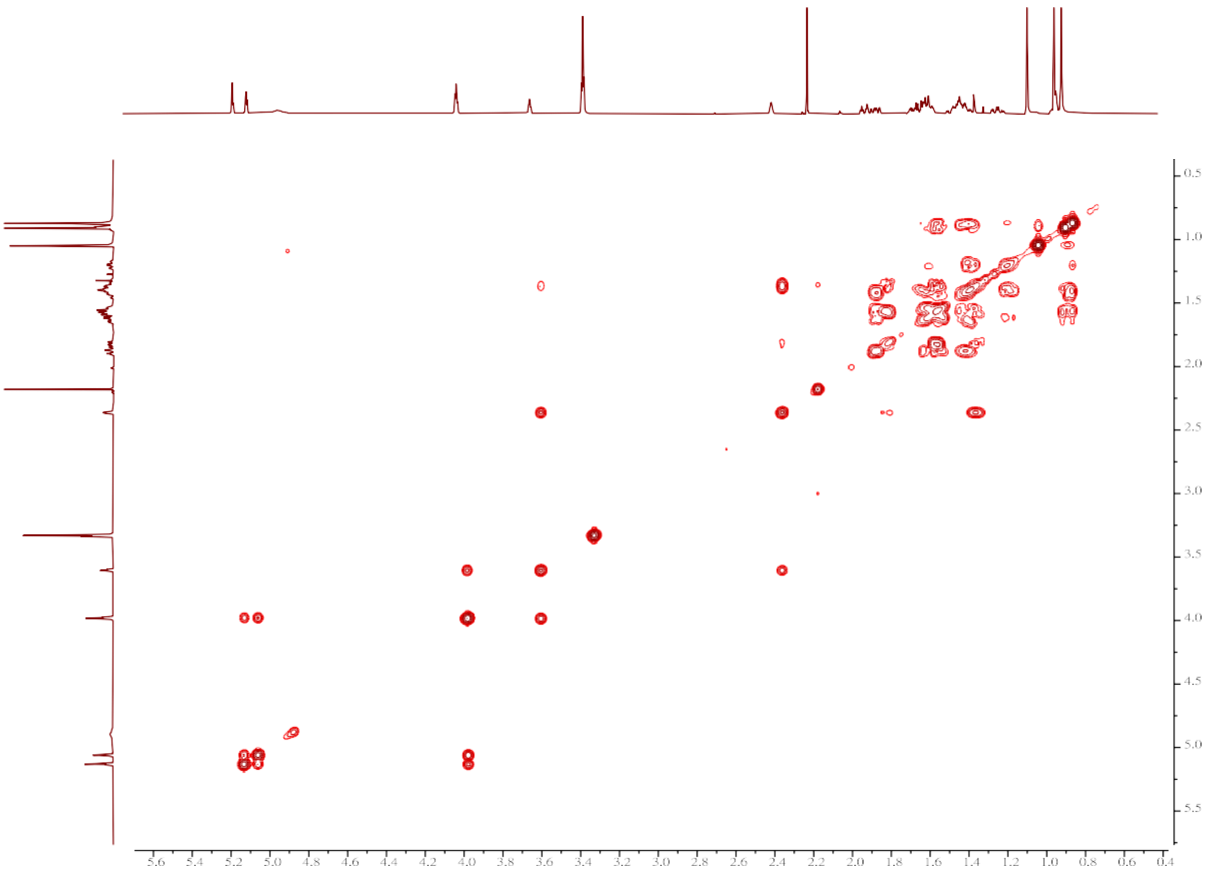

Figure S65. COSY spectrum of mesonol J (**10**)

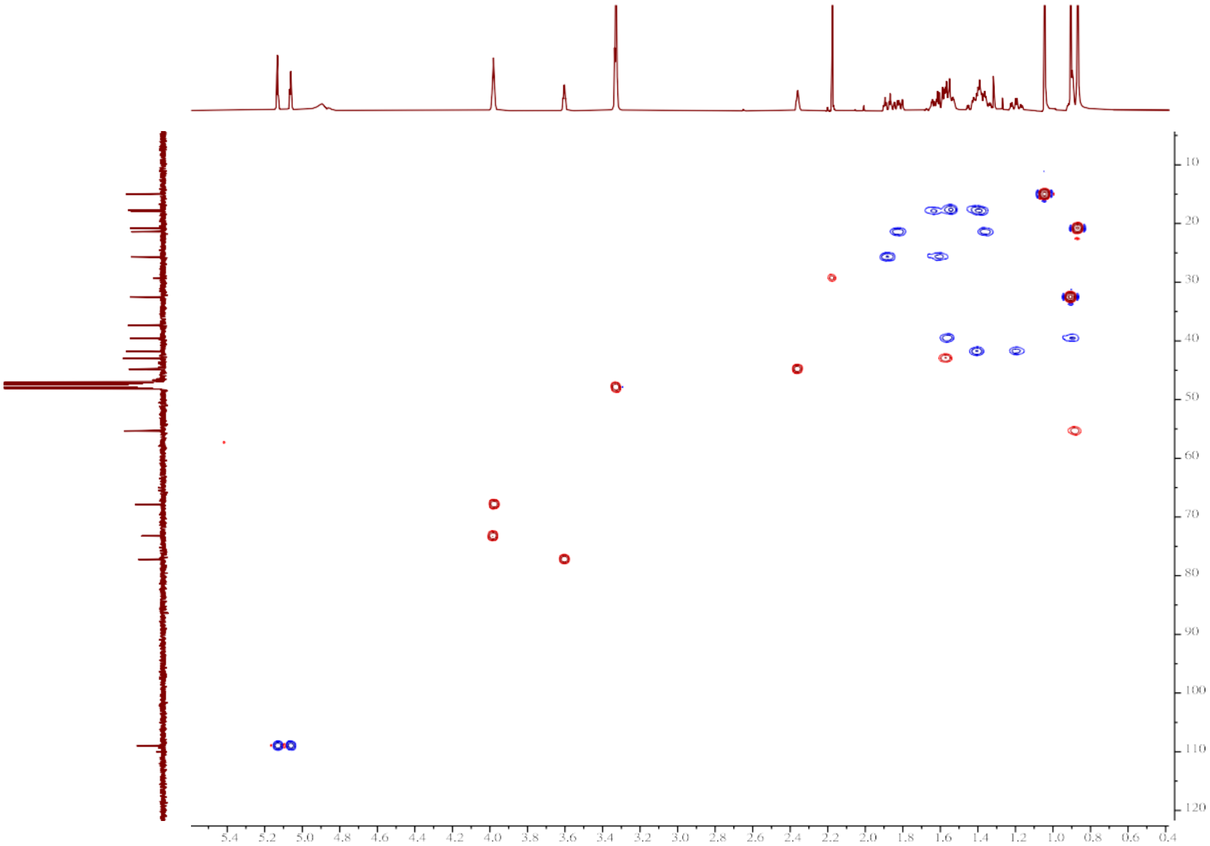

Figure S66. HSQC spectrum of mesonol J (**10**)

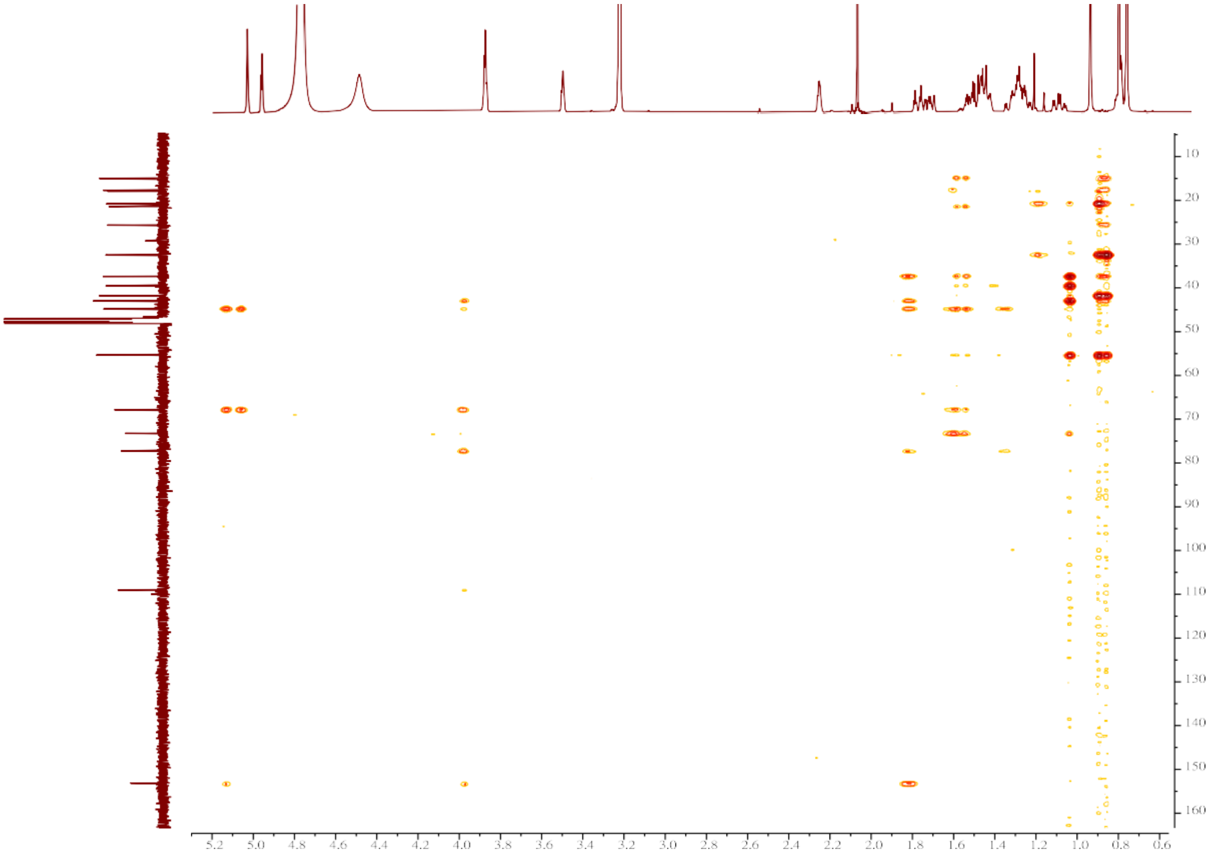

Figure S67. HMBC spectrum of mesonol J (**10**)

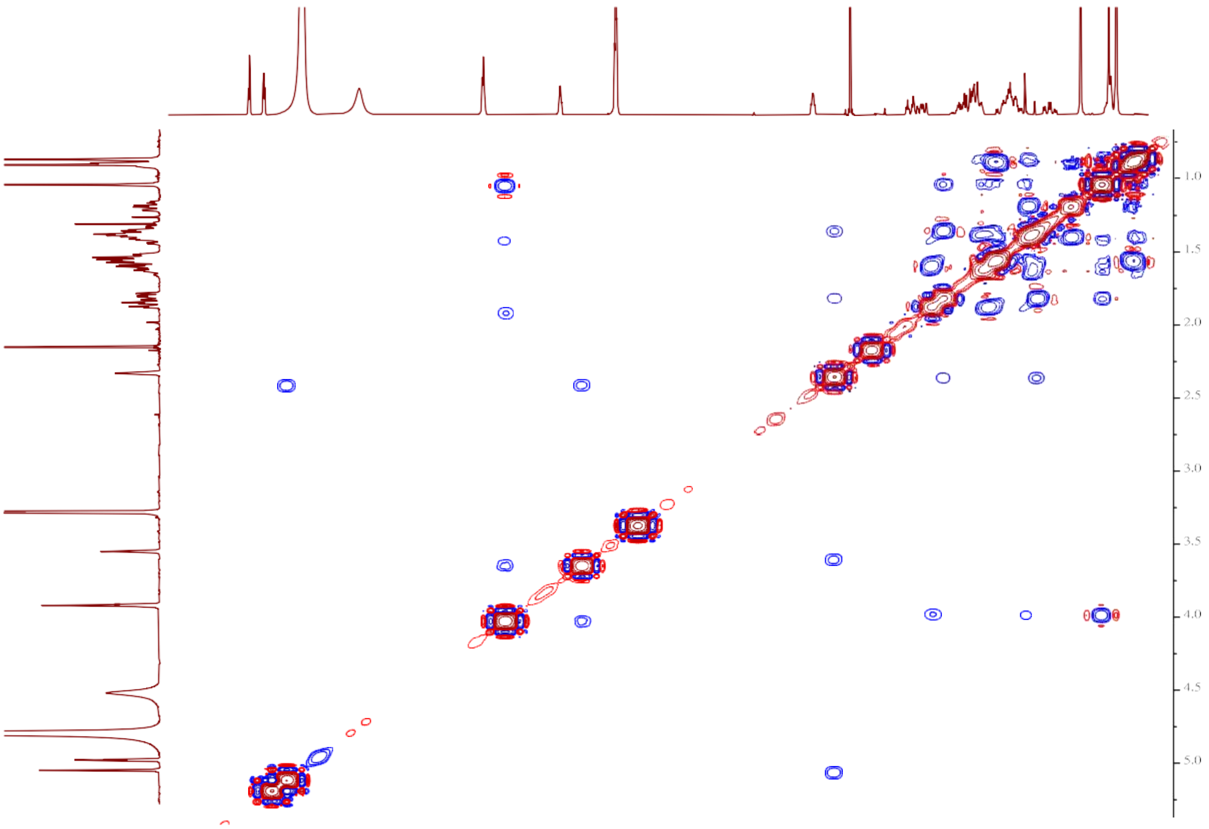

Figure S68. NOESY spectrum of mesonol J (**10**)

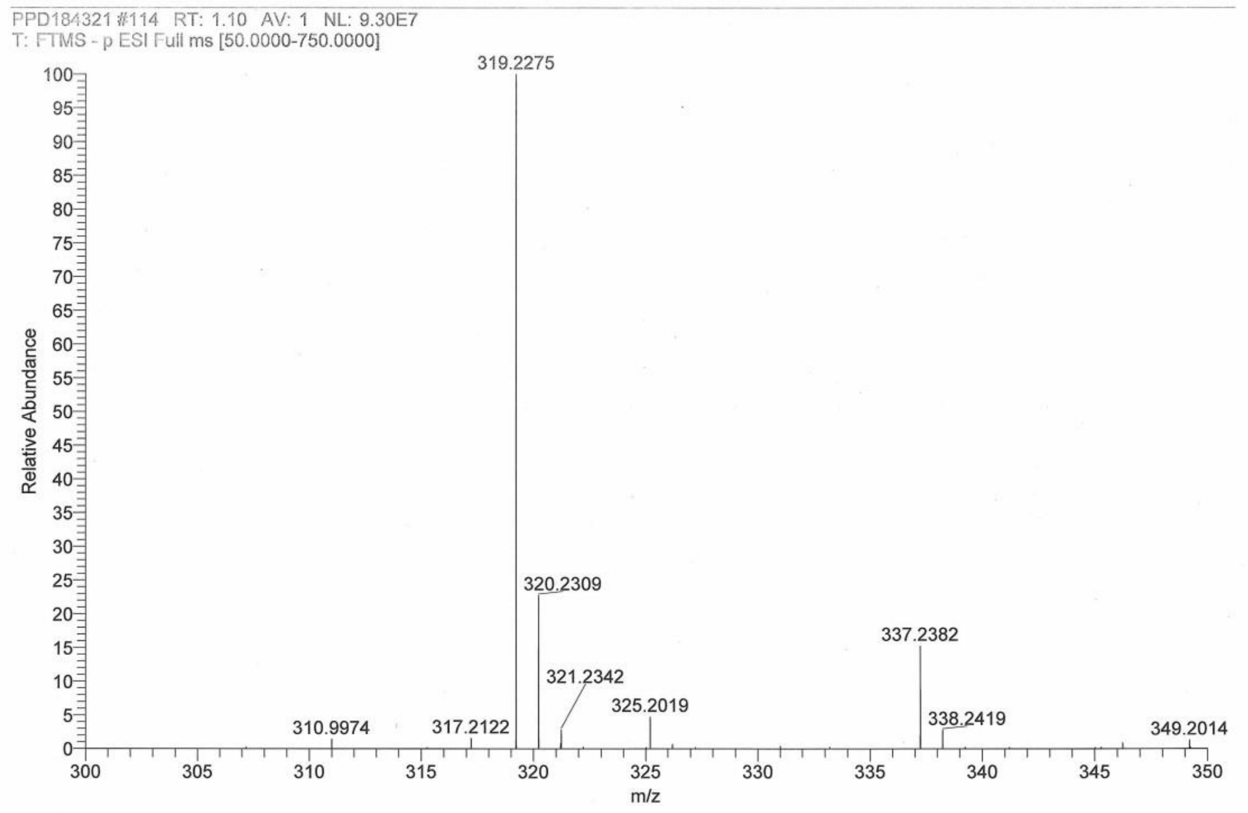

Figure S69. (−)-HRESIMS spectrum of mesonol J (**10**)

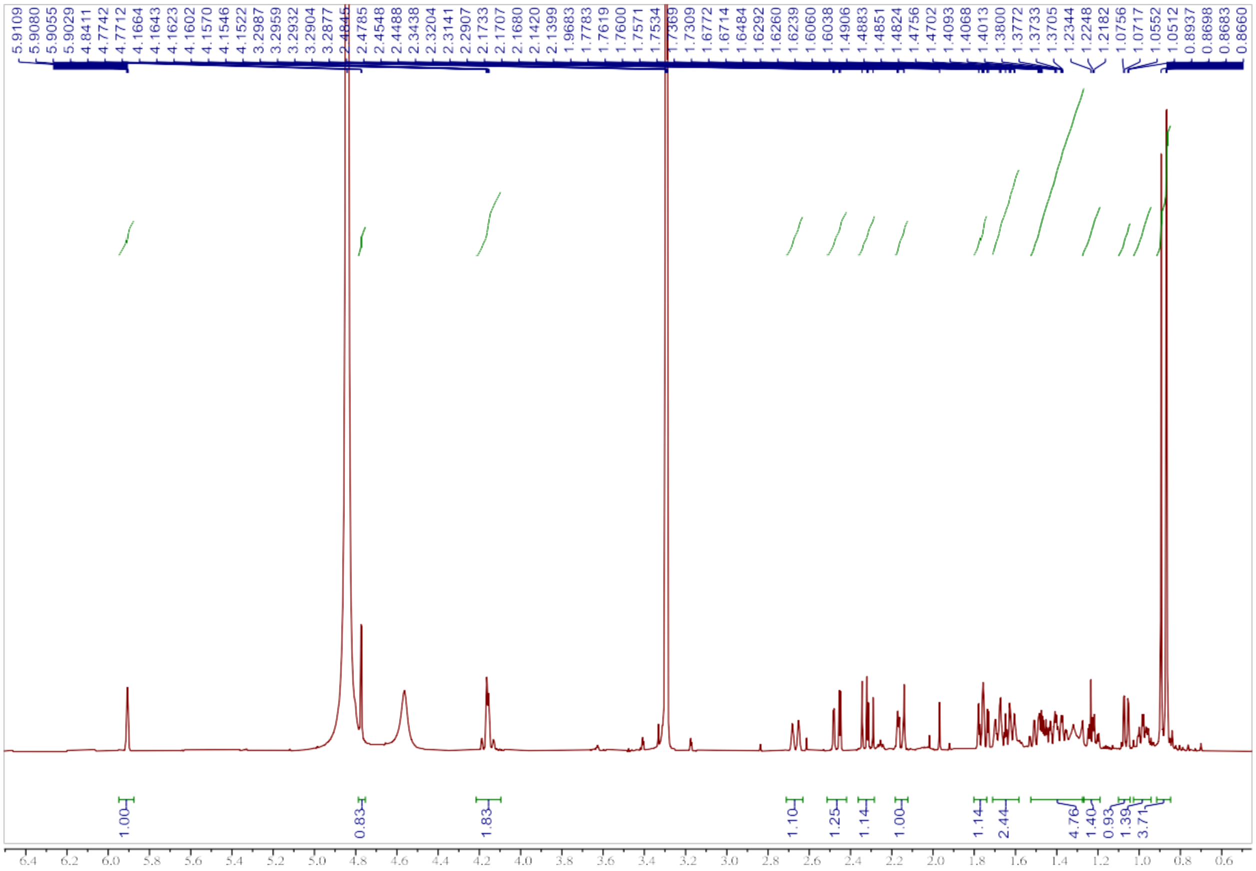

Figure S70. ^{1}H-NMR spectrum of mesonol K (**11**) in MeOD-*d*4 (600 MHz)

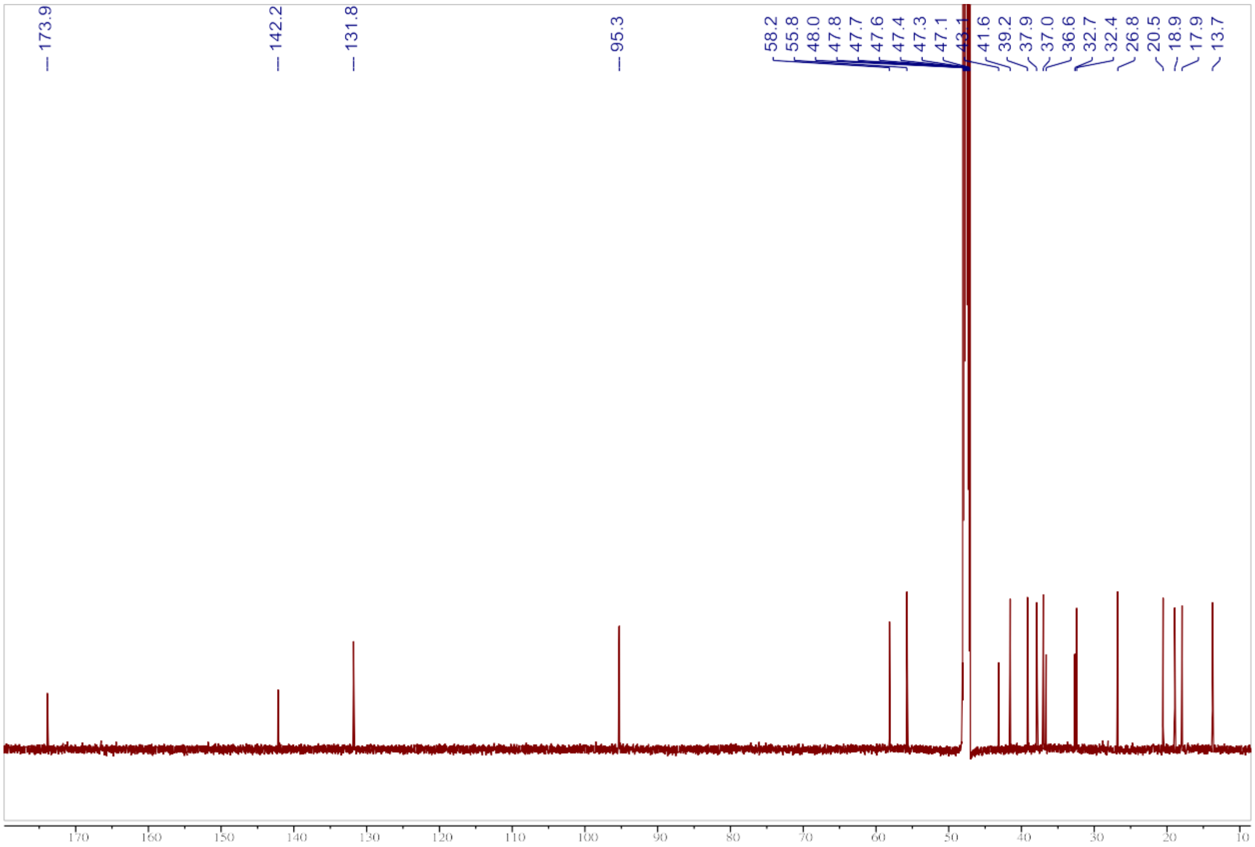

Figure S71. ^{13}C-NMR spectrum of mesonol K (**11**) in MeOD-*d*4 (150 MHz)

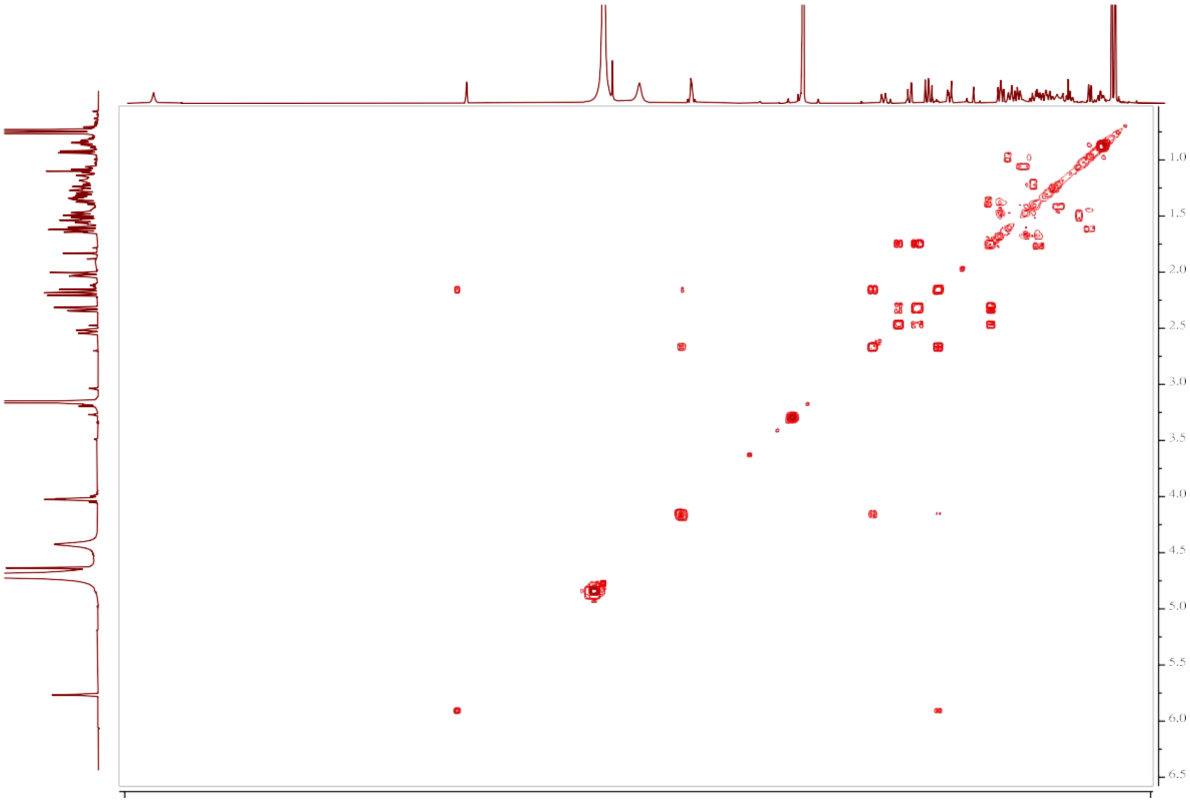

Figure S72. COSY spectrum of mesonol K (**11**)

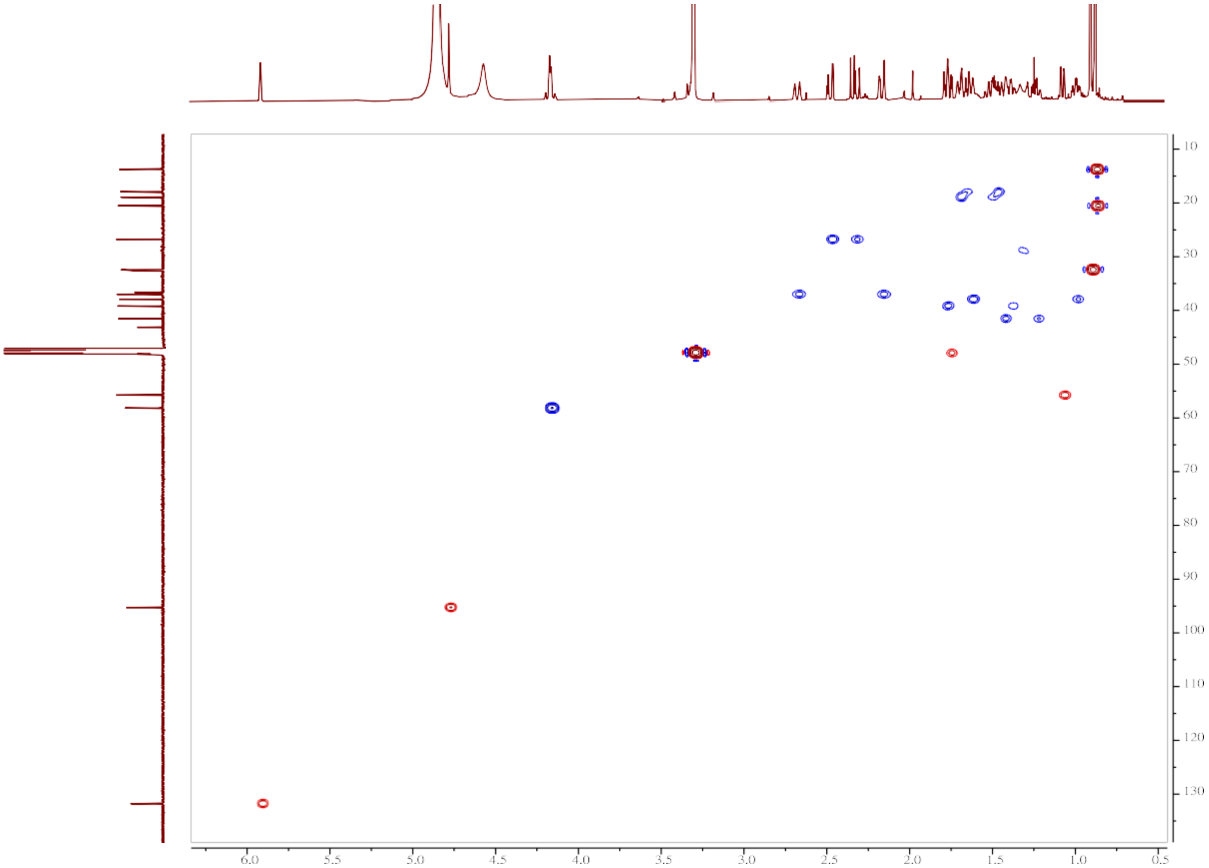

Figure S73. HSQC spectrum of mesonol K (**11**)

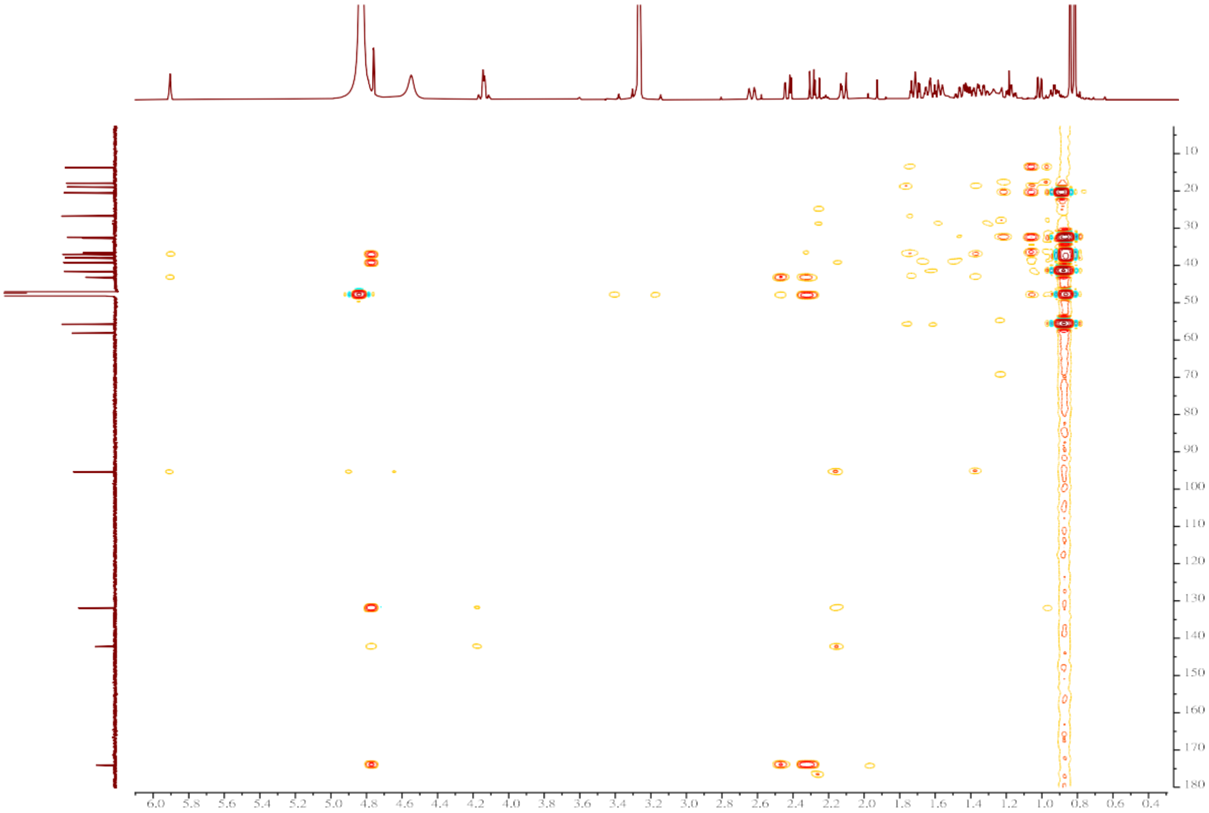

Figure S74. HMBC spectrum of mesonol K (**11**)

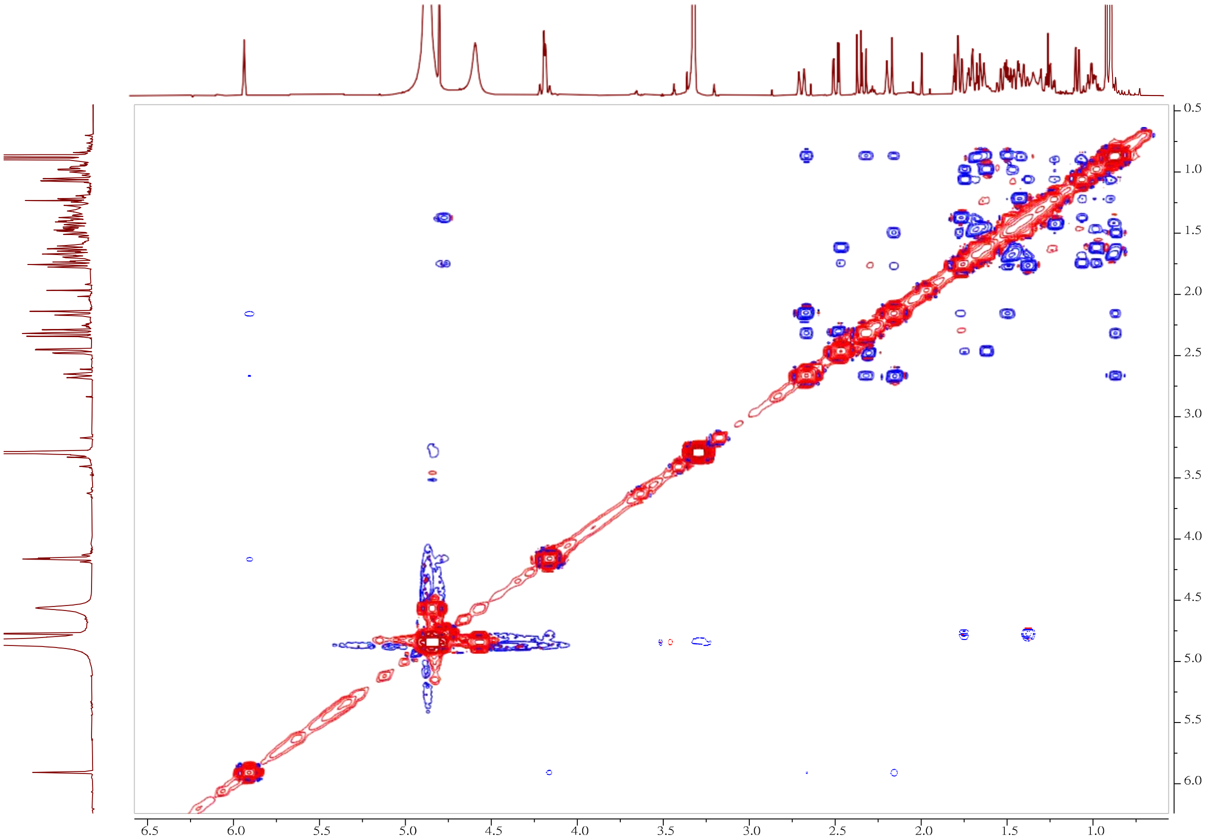

Figure S75. NOESY spectrum of mesonol K (**11**)

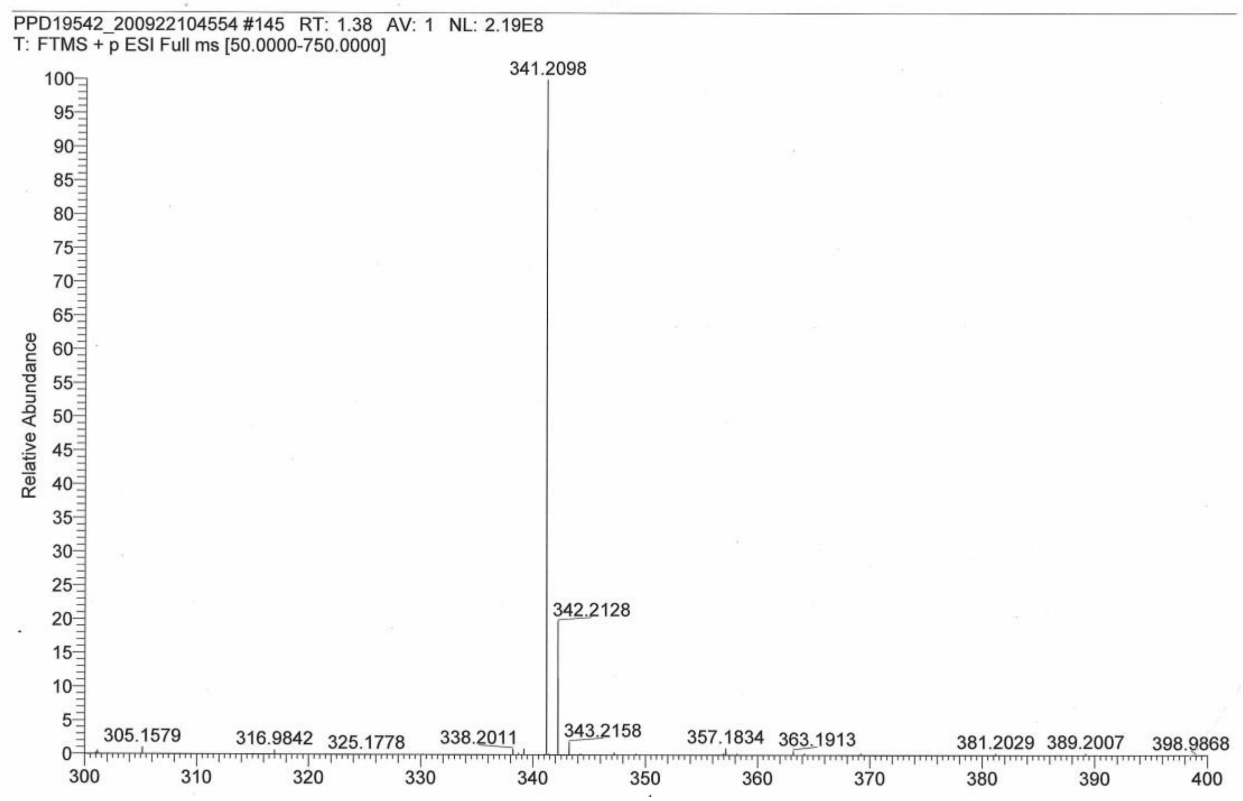

Figure S76. (+)-HRESIMS spectrum of mesonol K (**11**)

Supplementary tables

Table S1. Crystal data and structure refinement for **1** (d23335a, CCDC number: 2088866).

Identification code	d23335a	
Empirical formula	C20 H32 O4	
Formula weight	336.46	
Temperature	200(2) K	
Wavelength	1.54178 Å	
Crystal system	Monoclinic	
Space group	C 2	
Unit cell dimensions	a = 12.9593(6) Å	α= 90°.
	b = 6.5034(3) Å	β= 90.718(2)°.
	c = 21.1991(10) Å	γ = 90°.
Volume	1786.51(14) Å^3	
Z	4	
Density (calculated)	1.251 Mg/m^3	
Absorption coefficient	0.680 mm^{-1}	
F(000)	736	
Crystal size	0.38 x 0.24 x 0.02 mm^3	
Theta range for data collection	4.17 to 66.71°.	
Index ranges	-15<=h<=15, -7<=k<=7, -25<=l<=24	
Reflections collected	13838	
Independent reflections	3092 [R(int) = 0.0680]	
Completeness to theta = 66.71°	98.9 %	
Absorption correction	multiscan	
Max. and min. transmission	0.9865 and 0.7823	
Refinement method	Full-matrix least-squares on F^2	
Data/restraints/parameters	3092/4/215	
Goodness-of-fit on F^2	1.075	
Final R indices [I>2sigma(I)]	R1 = 0.0822, wR2 = 0.2003	
R indices (all data)	R1 = 0.0882, wR2 = 0.2127	
Absolute structure parameter	0.1(4)	
Largest diff. peak and hole	0.386 and -0.432 e.Å^{-3}	

Table S2. Crystal data and structure refinement for **2** (d22559, CCDC number: 2088867).

Identification code	d22559	
Empirical formula	C20 H32 O3	
Formula weight	320.46	
Temperature	200(2) K	
Wavelength	0.71073 Å	
Crystal system	Monoclinic	
Space group	C 2	
Unit cell dimensions	a = 42.054(9) Å	α= 90°.
	b = 6.9024(13) Å	β= 105.372(5)°.
	c = 12.722(3) Å	γ = 90°.
Volume	3560.7(12) $Å^3$	
Z	8	
Density (calculated)	1.196 Mg/m^3	
Absorption coefficient	0.078 mm^{-1}	
F(000)	1408	
Crystal size	0.70 x 0.02 x 0.01 mm^3	
Theta range for data collection	2.24 to 25.04°.	
Index ranges	-50<=h<=50, -8<=k<=8, -15<=l<=15	
Reflections collected	34474	
Independent reflections	6269 [R(int) = 0.1615]	
Completeness to theta = 25.04°	99.6 %	
Absorption correction	multiscan	
Max. and min. transmission	0.9992 and 0.9474	
Refinement method	Full-matrix least-squares on F^2	
Data/restraints/parameters	6269/1/415	
Goodness-of-fit on F^2	1.050	
Final R indices [I>2sigma(I)]	R1 = 0.0635, wR2 = 0.1160	
R indices (all data)	R1 = 0.1863, wR2 = 0.1599	
Absolute structure parameter	-1.6(19)	
Largest diff. peak and hole	0.241 and -0.265 e.$Å^{-3}$	

Table S3. Crystal data and structure refinement for **3** (d22583, CCDC number: 2088868).

Identification code	d22583	
Empirical formula	C20 H34 O4	
Formula weight	338.47	
Temperature	200(2) K	
Wavelength	0.71073 Å	
Crystal system	Monoclinic	
Space group	C 2	
Unit cell dimensions	a = 11.599(11) Å	α= 90°.
	b = 7.391(7) Å	β= 101.71(3)°.
	c = 21.59(2) Å	γ = 90°.
Volume	1812(3) $Å^3$	
Z	4	
Density (calculated)	1.241 Mg/m^3	
Absorption coefficient	0.084 mm^{-1}	
F(000)	744	
Crystal size	0.11 x 0.10 x 0.01 mm^3	
Theta range for data collection	2.89 to 25.05°.	
Index ranges	-12<=h<=13, -8<=k<=8, -25<=l<=25	
Reflections collected	10913	
Independent reflections	3171 [R(int) = 0.0599]	
Completeness to theta = 25.05°	99.0 % Absorption correction	multiscan
Max. and min. transmission	0.9992 and 0.9908	
Refinement method	Full-matrix least-squares on F^2	
Data/restraints/parameters	3171/3/218	
Goodness-of-fit on F^2	1.073	
Final R indices [I>2sigma(I)]	R1 = 0.0975, wR2 = 0.2424	
R indices (all data)	R1 = 0.1334, wR2 = 0.2670	
Absolute structure parameter	-1(4)	
Largest diff. peak and hole	0.570 and -0.384 e.$Å^{-3}$	

Table S4 Crystal data and structure refinement for **7** (d22561, CCDC number: 2088869).

Identification code	d22561	
Empirical formula	C40 H67 O9	
Formula weight	691.94	
Temperature	200(2) K	
Wavelength	0.71073 Å	
Crystal system	Monoclinic	
Space group	P 21	
Unit cell dimensions	a = 7.3577(8) Å	α= 90°.
	b = 11.1296(9) Å	β= 95.745(3)°.
	c = 22.919(2) Å	γ = 90°.
Volume	1867.4(3) Å^3	
Z	2	
Density (calculated)	1.231 Mg/m^3	
Absorption coefficient	0.085 mm^{-1}	
F(000)	758	
Crystal size	0.74 x 0.39 x 0.02 mm^3	
Theta range for data collection	2.04 to 25.08°.	
Index ranges	-8<=h<=8, -12<=k<=13, -24<=l<=27	
Reflections collected	27861	
Independent reflections	6563 [R(int) = 0.0968]	
Completeness to theta = 25.08°	99.7 %	
Absorption correction	multiscan	
Max. and min. transmission	0.9983 and 0.9397	
Refinement method	Full-matrix least-squares on F^2	
Data/restraints/parameters	6563/1/452	
Goodness-of-fit on F^2	1.025	
Final R indices [I>2sigma(I)]	R1 = 0.0732, wR2 = 0.1893	
R indices (all data)	R1 = 0.0922, wR2 = 0.2076	
Absolute structure parameter	0.1(13)	
Largest diff. peak and hole	0.502 and -0.389 e.Å^{-3}	
